# Five-stage ordering to a topological-defect-mediated ground state in a buckyball artificial spin ice

**DOI:** 10.1038/s42005-026-02663-y

**Published:** 2026-05-26

**Authors:** Gavin M. Macauley, Luca Berchialla, Peter M. Derlet, Laura J. Heyderman

**Affiliations:** 1 Laboratory for Mesoscopic Systems, Department of Materials, Zurich, Switzerland; 2 PSI Center for Neutron and Muon Sciences, Villigen, PSI Switzerland; 3 PSI Center for Scientific Computing, Theory and Data, Villigen, PSI Switzerland; 4 Present Address: Department of Physics, Princeton University, Princeton, NJ USA

**Keywords:** Phase transitions and critical phenomena, Magnetic properties and materials, Ferromagnetism

## Abstract

Artificial spin ices are arrays of coupled nanomagnets, which exhibit a variety of fascinating collective behaviour including emergent magnetic monopoles and novel phase transitions. However, they have mainly been confined to two dimensions due to the challenges inherent to their fabrication and characterisation in three dimensions. Exploiting the third dimension offers new degrees of freedom that can lead to curvature-induced topological effects. Here, using numerical simulations, we investigate the magnetic behaviour of a finite three-dimensional spin lattice: the buckyball artificial spin ice, where the spins are located on the edges of a regular buckyball. This frustrated system has a non-trivial topology that results in a rich spectrum of thermal magnetic behaviour, beginning with a crossover from a Paramagnetic sector to a Spin-Ice sector, followed by the formation of an imperfect charge crystal, before spin order is established in three separate steps. The ground state configuration is described by a pair of robust topological magnetic defects that arise because of the finite curved nature of the lattice. Our work reveals the complex thermodynamics of the buckyball artificial spin ice and paves the way to designing unusual magnetic textures in other curved three-dimensional nanomagnetic systems, exploiting the interplay between topology and the dipolar interaction.

## Abstract (web summary)

Artificial spin ices offer a way to explore emergent magnetic phenomena but are typically confined to flat geometries. Using Monte Carlo simulations, the authors determine the temperature-dependent behaviour of a 3D artificial spin ice based on the buckyball and show that it undergoes five crossovers on cooling, culminating in a ground state with a pair of topological defects at its poles.

## Introduction

The paradigm that complex emergent behaviour can arise from combining simple building blocks^1^ is neatly epitomised by artificial spin ices. These arrays of coupled nanomagnets^2^ can host complex collective behaviour including emergent magnetic monopoles^3,4^, vertex frustration^5^ and entropy-driven phase transitions^6^. Originally envisaged as an experimentally tractable mesoscopic analogue to the rare-earth pyrochlores^7^, artificial spin ices now offer a route to realising a plethora of model systems that address several important questions in statistical physics, and make it possible to explore a host of lattices, even going beyond those found in nature^8–11^. By carefully tailoring the design of the lattice, and making use of modern lithographic techniques, different symmetries and spin degrees of freedom can be realised in artificial spin ices^12–14^, and their magnetic order can then be inspected in real space to observe thermal relaxation^15^ and phase transitions^16^.

Until now, artificial spin ices have mainly been created in two dimensions (2D), since it is challenging to manufacture and characterise three-dimensional (3D) nanostructures. In order to observe their phase transitions, such 2D systems are made large enough to approach the bulk thermodynamic limit, avoiding finite size effects that smear out the appearance of critical phenomena^17^. Here we take a different approach and explicitly target the fascinating magnetic textures that can be hosted by a finite spin lattice with curvature, namely one based on the buckyball. The buckyball is a truncated icosahedron, a solid with both hexagonal and pentagonal faces, whose most widely-known realisation in condensed matter is the C_60_ Buckminsterfullerene allotrope of carbon^18^.

We construct our lattice by placing a point magnetic dipole on the midpoint of each edge of a regular buckyball, and term the resulting system the buckyball artificial spin ice. In our framework, the spins have an Ising degree of freedom and so are constrained to point in one of the two directions parallel to the orientation of their associated edge, i.e. into one vertex and out from the other. Previously, the magnetic-field-induced behaviour and the microwave response of such a connected lattice have been simulated using finite element techniques^19,20^, showing that high-energy excitations can be seeded by an appropriately applied magnetic field.

Using Monte Carlo simulations, we reveal the thermodynamics of the buckyball artificial spin ice, which exhibits five crossovers as a function of temperature. These crossovers separate a wide hinterland of magnetic orders based on cooperative spin structures occurring at different length scales. At high temperatures, the buckyball crosses over from a Paramagnetic sector to a Spin-Ice sector, which is characterised by short-range correlations and an ice rule minimising the net number of spins that point into the vertices. On cooling the buckyball further, the net magnetic charges at all of the vertices order across the lattice. However, perfect charge order cannot be accommodated because of the topology of buckyball, resulting in an imperfect charge crystal where the spins continue to fluctuate. Spin order is then established in three separate stages, with the ground state described by a pair of robust topological defects, whose location can be controlled with an applied magnetic field.

The richness of the behaviour arises because the spins form head-to-tail loops, which perceive the entire extent of the finite lattice. What then makes these non-local loop structures—a hallmark of a system with spin ice physics^21^—special is that they are embedded in a lattice with a non-trivial topology, which is effectively wrapped in on itself. Here, long-range interactions between spins on opposite sides of the buckyball, mediated by these loops of head-to-tail spins, promote the unusual low-temperature crossovers. Indeed, due to the curvature of the lattice, further-out neighbouring spins are closer than in a comparable, planar system.

The buckyball artificial spin ice is highly frustrated and many of the crossovers occur at temperature scales well below the nearest-neighbour interaction. This frustration arises through competing interactions on different length scales. Firstly, there is a short-range frustration at the level of individual vertices, which exhibit a preference for an ice-rule-obeying vertex configuration featuring one unsatisfied interaction. Then, frustration arises at an intermediate length scale due to the impossibility of achieving a perfect arrangement of magnetic charges on the surface of the buckyball. It is energetically favorable for every positive vertex charge to be surrounded by nearby vertices with a negative charge and vice versa. However, perfect charge order cannot be accommodated everywhere, leading to a frustration associated with their placement. Lastly, frustration arises on a larger length scale through the creation of topological defects. These defects can seed in various locations, giving rise to a configurational entropy.

This work highlights that finite 3D lattices can host unusual magnetic textures that have no obvious counterpart in naturally occurring microscopic materials. While we have limited ourselves to a computational investigation, we foresee that such 3D lattices based on Platonic and Archimedean solids could be routinely realised using existing lithographic techniques^22–28^. However, magnetic imaging remains challenging, requiring further developments to capture the thermodynamics^29^.

Such difficulties notwithstanding, the buckyball artificial spin ice is a particularly promising platform for advancing our understanding of 3D artificial spin ices. Its close relation to the well-studied 2D artificial kagome spin ice suggests that insights gained from 2D systems could provide a valuable foundation for interpreting the magnetic behaviour of buckyball and other similar finite 3D structures. Indeed, now that magnetic buckyball lattices have already been fabricated^22,23,30–33^, imaging will be the crucial next step towards confirming our theoretical predictions about the different types of magnetic order that can emerge.

The buckyball artificial spin ice can be viewed as a mesoscale analogue of a molecular magnet^34,35^. In molecular magnets, free magnetic moments are introduced at specific sites within molecules through careful synthesis. Interactions between these moments can then give rise to unusual phases such as quantum spin liquids^36^. Likewise, in the buckyball artificial spin ice, we find that exotic magnetic order emerges from the combined local and global constraints imposed by the molecular framework.

## Results

### Thermal properties of the buckyball artificial spin ice

The buckyball is one of the 13 Archimedean solids, which are solids characterised by having faces that are two or more types of regular polygons. The buckyball can be constructed from an icosahedron by truncating each of its vertices one third of the way along each edge. For this reason, the geometric name of the buckyball is the truncated icosahedron. The truncation results in both pentagonal and hexagonal faces of equal side lengths. The buckyball has 60 vertices, 90 edges, and 32 faces, of which 12 are pentagons and 20 are hexagons. Three edges meet at any vertex, and the buckyball possesses vertex transitivity, so that all vertices are alike in terms of their local surroundings. The edges of a buckyball can be sorted into two types: those that connect two hexagons, and those that connect a hexagon to a pentagon.

**Fig. 1:**
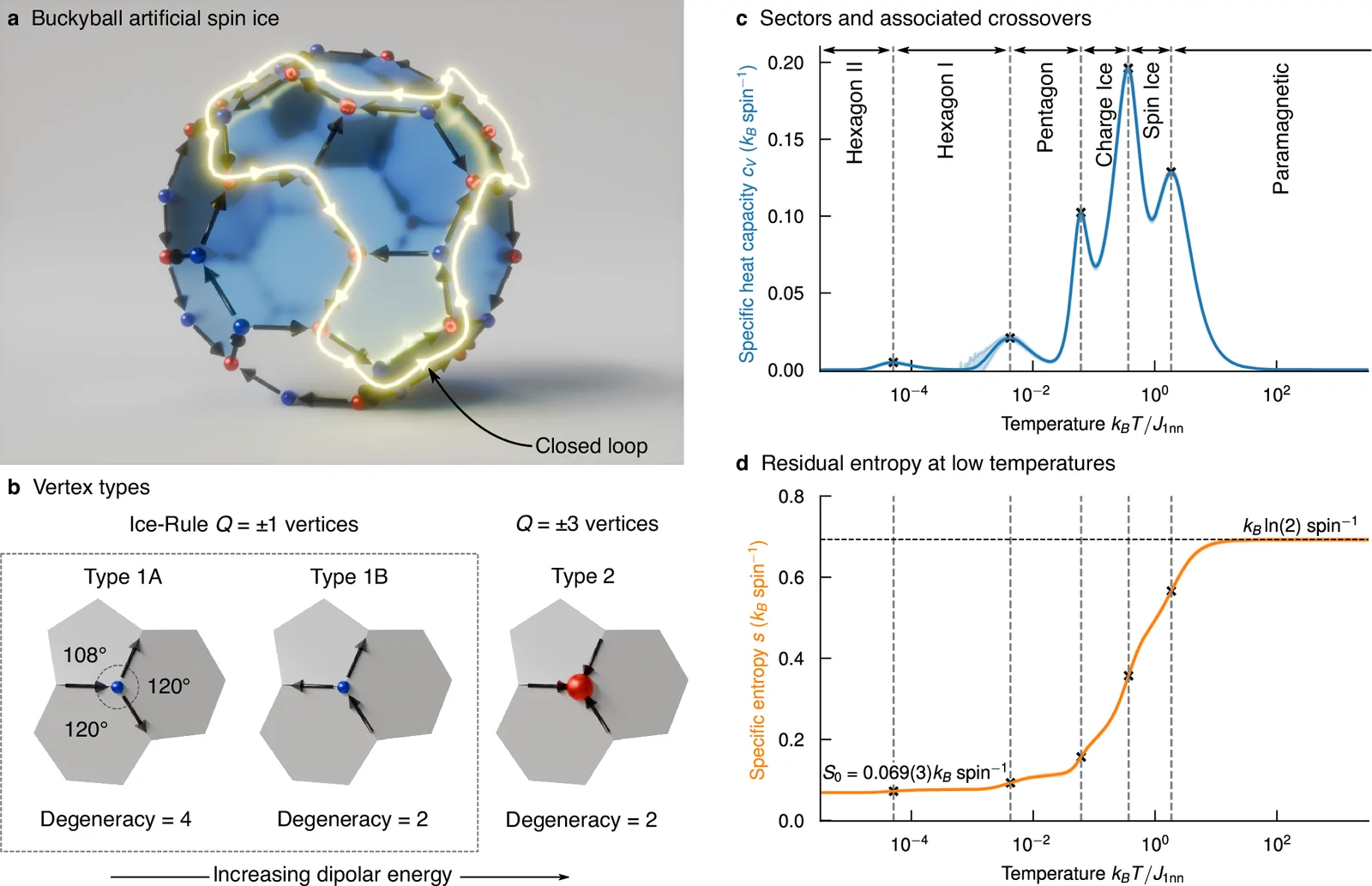
Thermal properties of the buckyball artificial spin ice. **a** The buckyball artificial spin ice is constructed by placing point magnetic dipoles, i.e., spins, on the midpoints of the edges of a regular buckyball. These spins have an Ising degree of freedom and are constrained to point in one of two directions along an edge. A closed loop of head-to-tail spins is highlighted. **b** Example spin and vertex charge configurations for the three vertex types, with the point dipolar energy increasing from left to right. Ice-rule-obeying *Q* = ± 1 vertex configurations have three spins that are either in a two-in/one-out or two-out/one-in configuration, but differ in energy depending on whether the two spins associated with a pentagonal face are in a favourable head-to-tail alignment (Type 1A configuration) or unfavourable alignment (Type 1B configuration). In a high-energy *Q* = ± 3 vertex configuration, all three spins point either in or out, and we refer to these as Type 2 vertex configurations. The degeneracy of each vertex configuration is indicated. **c**, **d** Temperature dependence of (**c**) the specific heat capacity *c*_V_ of the buckyball artificial spin ice, which exhibits five peaks, and (**d**) the corresponding entropy *s*. The five peaks in the specific heat capacity are indicated with crosses. The corresponding temperatures at which these peaks occur are marked by dashed vertical lines in both (**c**, **d**) and coincide with points of inflection in the entropy, also indicated with crosses. On cooling, each of the peaks is associated with a crossover to a new type of magnetic order, and the names we give to each of the sectors are labelled explicitly in (**c)**. The buckyball artificial spin ice still possesses a residual entropy at *T* = 0 of *S*_0_ = 0.069(3) *k*_*B*_ per spin as indicated. Since each spin has an Ising degree of freedom, the high-temperature limit of the specific entropy is ${k}_{B}\ln(2)$ per spin. In (**c**, **d**) shaded regions demarcate  ± 1*σ* around the mean of 5000 independent simulation runs.

The buckyball artificial spin ice is constructed by placing a point magnetic dipole at the midpoint of each edge of a regular buckyball, as depicted in Fig. 1a. We further constrain each point dipole to point in one of two directions along its associated edge. In effect, each dipole represents the macrospin of an isolated single-domain nanomagnet, which is the building block common to many experimental artificial spin ices^2^.

The conventional 2D artificial spin ice that most closely resembles our 3D buckyball version is the artificial kagome spin ice. Indeed, the buckyball lattice bears similarities to a kagome lattice that has been wrapped around on itself to leave no boundaries. However, since such a wrapping cannot be seamless, the buckyball includes pentagonal as well as hexagonal faces. In this sense, we can view the buckyball lattice as a kagome lattice in which an ordered arrangement of pentagonal defects has been introduced to accommodate the curvature^37^. The phase diagram of the 2D artificial kagome spin ice^38–40^ features one crossover from a high-temperature paramagnetic phase to a spin-ice phase. On decreasing the temperature further, this is followed by two phase transitions, first from the spin-ice phase to a charge-ordered phase and then finally to a long-range spin-ordered state. The ground state of the 2D artificial kagome spin ice is six-fold degenerate, and is characterised by the head-to-tail alignment of spins associated with two out of every three hexagonal plaquettes—a configuration referred to as the ‘loop’^38^ or ‘vortex’^41^ configuration. In general, head-to-tail arrangements of magnetic moments minimise the stray magnetic field, so that we would expect the low-energy states of the buckyball artificial spin ice to feature them. Indeed, one such closed loop is highlighted in Fig. 1a.

At each vertex in our buckyball lattice, we define a net effective magnetic charge *Q*, which is reflected by the number of spins that point into a vertex less the number that point out. Low-energy vertices are those that obey an ice-rule: *Q* = ± 1 with either a two-in/one-out or two-out/one-in spin configuration. Unlike the artificial kagome spin ice, where all *Q* = ± 1 vertices have the same energy, ice-rule-obeying vertex configurations in the buckyball artificial spin ice can be split into two categories (see Fig. 1b). This arises because the internal angles of a regular pentagon of 108^∘^ are smaller than the internal angles of a regular hexagon of 120^∘^. As a consequence, at each vertex, the two spins that are associated with a pentagonal face are slightly closer together than either of them is to the remaining spin that bridges two hexagonal faces. Of the six possible ice-rule-obeying vertex configurations for a single vertex, four feature a favourable head-to-tail alignment of these pentagonal spins. These Type 1A vertices are the lowest energy configurations for a single vertex. The remaining two ice-rule-obeying vertex configurations, which we denote Type 1B, feature an unfavourable alignment of these pentagonal spins and are slightly higher in energy than the Type 1A vertices. The highest energy vertices, Type 2, have charge *Q* = ± 3 with all three spins pointing in or out, with one of these instances shown in Fig. 1b.

To simulate the thermodynamic behaviour of the buckyball artificial spin ice, we perform extensive Monte Carlo simulations using the Metropolis-Hastings algorithm^42,43^ incorporating both single spin flip dynamics and loop moves^44–46^ on a point-dipolar Hamiltonian. This Hamiltonian is

1 $${{{\mathscr{H}}}}=\frac{D}{2} \sum_i^N{\sum}_{j\ne i}^{N}\left[\frac{{\hat{{{{\bf{s}}}}}}_{i}\cdot {\hat{{{{\bf{s}}}}}}_{j}}{{r}_{ij}^{3}}-\frac{3({\hat{{{{\bf{s}}}}}}_{i}\cdot {{{{\bf{r}}}}}_{ij})({\hat{{{{\bf{s}}}}}}_{j}\cdot {{{{\bf{r}}}}}_{ij})}{{r}_{ij}^{5}}\right],$$

such that spin *i*, located at position **r**_*i*_, has a normalised moment ${\widehat{{{{\bf{s}}}}}}_{i}$. The vector **r**_*ij*_ ≡ **r**_*j*_ − **r**_*i*_ points from spin *i* to spin *j*, and *N* is the number of spins. The energy scale of the interaction is set through the dipolar constant, $D={\mu }_{0}{({M}_{S}V)}^{2}/(4\pi {a}^{3})$, where *M*_*S*_ and *V* are the saturation magnetisation and volume of each nanomagnet, respectively. The symbol *μ*_0_ denotes the magnetic permeability of free space. The parameter *a*, which is the lattice constant, controls the scale of the distances between spins. Here, we set *a* = 1 since the spin and vertex positions have been calculated for a buckyball with unit edge length. Further details concerning the Monte Carlo simulations are given in the Methods section and in Supplementary Notes 1–3.

In Fig. 1c, d, we show the behaviour of the specific heat capacity *c*_V_ (in blue) and the specific entropy *s* (in orange) as a function of the reduced temperature *k*_*B*_*T*/*J*_1nn_, where *J*_1nn_ is the first-nearest-neighbour interaction strength and *k*_*B*_ is the Boltzmann constant. Here, and in all figures where we show a quantity as a function of temperature, the temperature axis is a logarithmic scale. The first-nearest-neighbour interaction strength *J*_1nn_ is the magnitude of the dipolar interaction between the two vertex spins that each lie along the side of a pentagonal face. Surprisingly, the specific heat exhibits five peaks, with three at very low temperatures well below the temperature scale associated with the nearest-neighbour interaction. The highest-temperature peak is relatively broad and occurs at *k*_*B*_*T*/*J*_1nn_ = 1.85, which is of the order of the first-nearest-neighbour interaction. Subsequent peaks, at *k*_*B*_*T*/*J*_1nn_ ≈ 0.360, 0.061, 0.004 and 0.00006, become progressively sharper. The origin of these peaks is not immediately obvious, since artificial spin ices to date have exhibited at most three peaks that are often associated with the formation of an ice-rule-obeying sector, a charge-ordered phase and a long-range spin-ordered state. Moreover, it might be expected that finite-size effects would smear out the sharper peaks. The appearance of so many peaks for the buckyball artificial spin ice suggests the formation of different types of magnetic order, which are promoted by the topology of the lattice.

Each one of the peaks is associated with a point of inflection in the specific entropy *s* as new configurations suddenly become accessible at that temperature *T*. Despite the inequivalent interactions at vertices, the buckyball artificial spin ice remains a frustrated system as a whole, with a non-zero residual entropy, *S*_0_ = 0.069(3) *k*_*B*_ per spin. Here, and in the Supplementary Information, we use the notation that the number in parentheses indicates the 1*σ* uncertainty in the last digit of the averaged quantity, where *σ* is the standard deviation. By definition, the residual entropy is related to the natural logarithm of the number of ground states Ω through ${S}_{0}=({k}_{B}/N)\ln(\Omega )$, where *N* = 90 is the number of spins. This suggests a ground state degeneracy of around 500 magnetic configurations.

If we do not incorporate loop moves in our simulations, only four peaks are obtained in the specific heat capacity. In this case, two of the low-temperature peaks are replaced by a single broad one at *k*_*B*_*T*/*J*_1nn_ ≈ 10^−2^ and the residual entropy is higher (Supplementary Note 1), which is consistent with the notion that loops moves are necessary to access topological sectors beyond the reach of single spin flip dynamics^21^. This effect is common to many spin ice models where the acceptance rate for single spin flips tends to zero before the onset of long-range order, and for which non-local dynamics must be explicitly introduced to maintain thermal equilibrium^47^. We also find that the most important loop moves involve the spins arranged around the individual pentagonal faces of the buckyball artificial spin ice (Supplementary Note 1).

In bulk systems, sharp peaks in the specific heat reflect non-analytical behaviour in the free energy, and thus phase transitions. In our finite system, the observed peaks indicate crossovers to different regimes of magnetic order, which will be here referred to as sectors. In the next section, we describe the spatial structure of the individual sectors that are labelled in Fig. 1c.

### Real-space spin and charge configurations

**Fig. 2:**
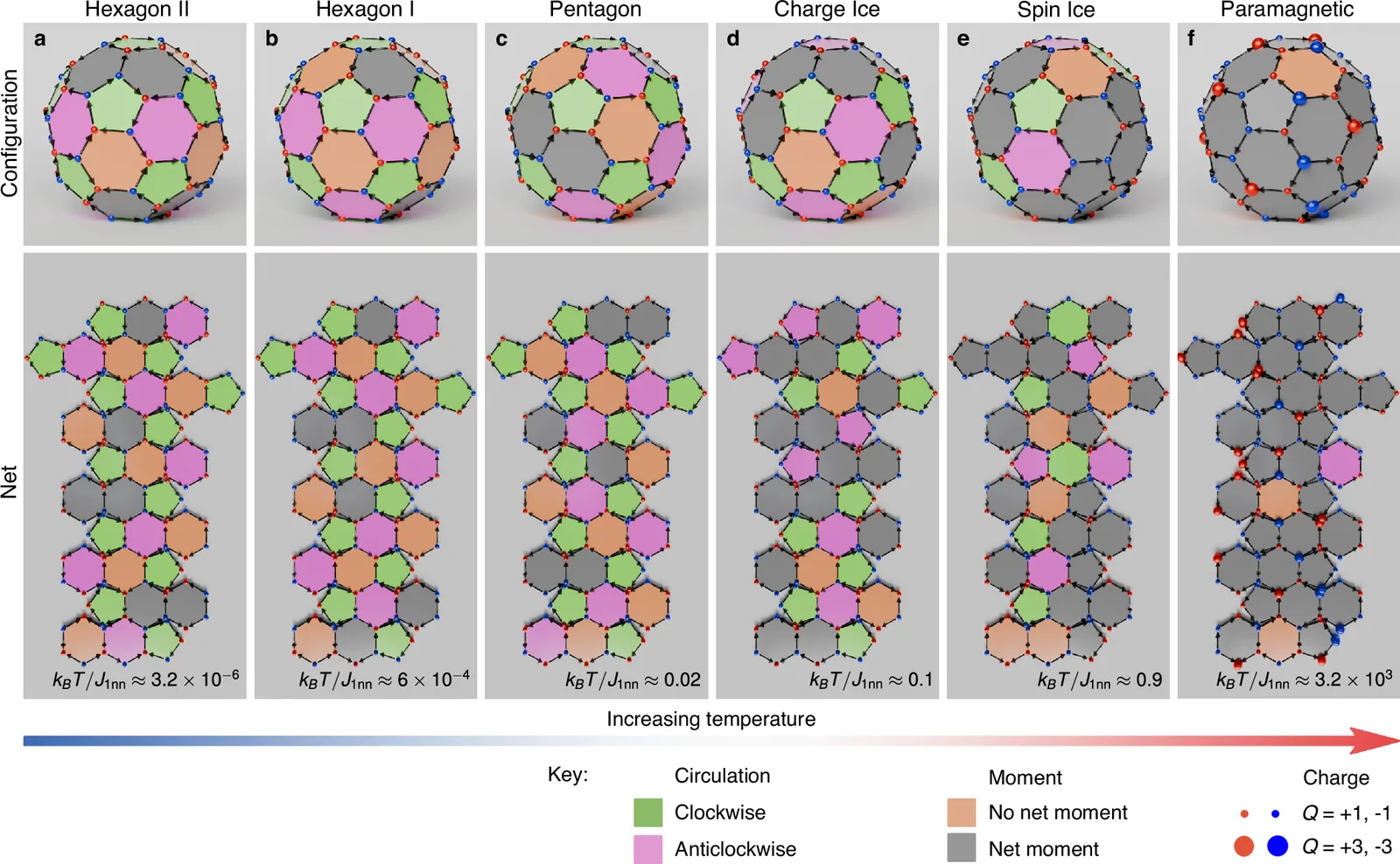
Example magnetic configurations taken from the six different sectors of the buckyball artificial spin ice. From low to high temperature, (**a**–**f**) are the Hexagon II, Hexagon I, Pentagon, Charge-Ice, Spin-Ice, and Paramagnetic sectors, respectively. Top sub-panels: the 3D lattice, showing the direction of the spins (black arrows) and the charge at each vertex. Positive (negative) charges are shown in red (blue) with the sizes indicating whether the charges are *Q* = ± 1 (small) or *Q* = ± 3 (large). Bottom sub-panels: net representations of the magnetic configuration. For both the 3D lattices and the net representations, the polygonal faces are coloured according to the configuration of their associated spins: green (pink) for a clockwise (anticlockwise) loop of spins, grey if the face has a net moment, and orange if the face has neither a net moment nor a closed loop. The sense of circulation is defined as that on viewing the buckyball from the outside along the normal to the given face. The reduced temperature is given in the bottom right of each net representation and increases from left to right. Supplementary Movies 1–6, corresponding to panels (**a**–**f**), respectively, depict the buckyball lattice rotating about the z-axis to reveal its full three-dimensional structure.

In Fig. 2, we show typical spin and charge configurations taken from each of the six different sectors of the buckyball artificial spin ice, with the temperature increasing from left to right as indicated. In addition to the 3D representation, we reveal the full configuration with a net representation, where the buckyball is unfolded into a single planar structure^48^ (see also Supplementary Movies 1–6). It should be noted that, while the number of faces in the net is the same as the number of faces in the solid, several vertices in the net may correspond to the same vertex in the solid, and similarly for edges. Each configuration is taken from the middle of the temperature range associated with that particular sector in order to avoid fluctuations in the energy (and, hence, the spin configuration) that are more common at the crossovers.

At high temperatures (Fig. 2f), the buckyball artificial spin ice is in a paramagnetic state, displaying neither spin nor charge order with both ice-rule-obeying *Q* = ± 1 and high-energy *Q* = ± 3 vertices present. Most faces—whether hexagonal or pentagonal—have a net moment, as expected for a state with randomly-oriented spins. As the buckyball artificial spin ice is cooled below *k*_*B*_*T*/*J*_1nn_ ≈ 1.85, it enters a Spin-Ice sector (Fig. 2e), where only *Q* = ± 1 vertices are present. At *k*_*B*_*T*/*J*_1nn_ ≈ 0.360, the charges on the vertices of the buckyball begin to order and each *Q* = + 1 vertex tries to surround itself with three *Q* = − 1 vertices, and vice versa. We therefore refer to this as the Charge-Ice sector. On closer inspection of the configuration in Fig. 2d, we see that the spins associated with each of the pentagons have aligned head-to-tail in a loop, although not all loops are necessarily in the same sense with the clockwise (anticlockwise) pentagons shown in green (pink). For the naming of these first three sectors, we have drawn a deliberate parallel with the phases of the artificial kagome spin ice^38,39,49^, although we will later make clear that there are significant differences. As the temperature continues to decrease, we see that, at *k*_*B*_*T*/*J*_1nn_ = 0.061, there is the first emergence of a type of spin order that spans the entire lattice: the spins around the pentagonal faces now have the same sense of circulation and, accordingly, they are all coloured green in Fig. 2c. We therefore label this the Pentagon sector. Some of the hexagons also have closed loops of spins but the ordering in terms of their sense of circulation is not correlated. Since such closed loops are naturally the arrangement that minimises the stray field, we will refer to them as flux-closed faces. The remaining two configurations (Fig. 2a, b) are taken from the two low-temperature sectors. These two sectors differ in how the hexagonal faces with a net moment are distributed across the surface of the solid, and we have thus named them Hexagon I and Hexagon II. In both of these sectors, six of the hexagonal faces have a net moment. We colour these faces grey according to the key in Fig. 2. We unravel what these two crossovers signify in greater detail later but, suffice to say, they stem from different groups of spins freezing at different temperatures in the buckyball artificial spin ice, leading to the formation of different low-energy motifs that together tile the lattice.

### Role of long-range interactions in promoting the low-temperature crossovers

To provide an explanation for the existence of the different magnetic sectors, and the crossovers that delineate them, we consider the pairwise spin and charge correlations in the buckyball artificial spin ice. This is particularly important for the low-temperature crossovers because, from the combined spin and vertex charge configurations alone, it is hard to say what defines exactly the Pentagon, Hexagon I, and Hexagon II sectors. Similarly, the three low-temperature crossovers are also not associated with any change in the loop statistics of the buckyball artificial spin ice, including the total number of loops, the maximum loop length, and the average loop length (Supplementary Note 5). Because the number of loops remains the same in the three low-temperature sectors, it follows that the number of flux-closed faces is identical in all of the three low-temperature sectors (Supplementary Note 1). While the spatial structure of the Spin-Ice sector and the Charge-Ice sector can be grasped from the real-space configurations, their formation in a finite lattice is still surprising. It is only by considering the spin and charge correlations that we gain a complete understanding of which interactions play a determining role in establishing the different magnetic sectors.

**Fig. 3:**
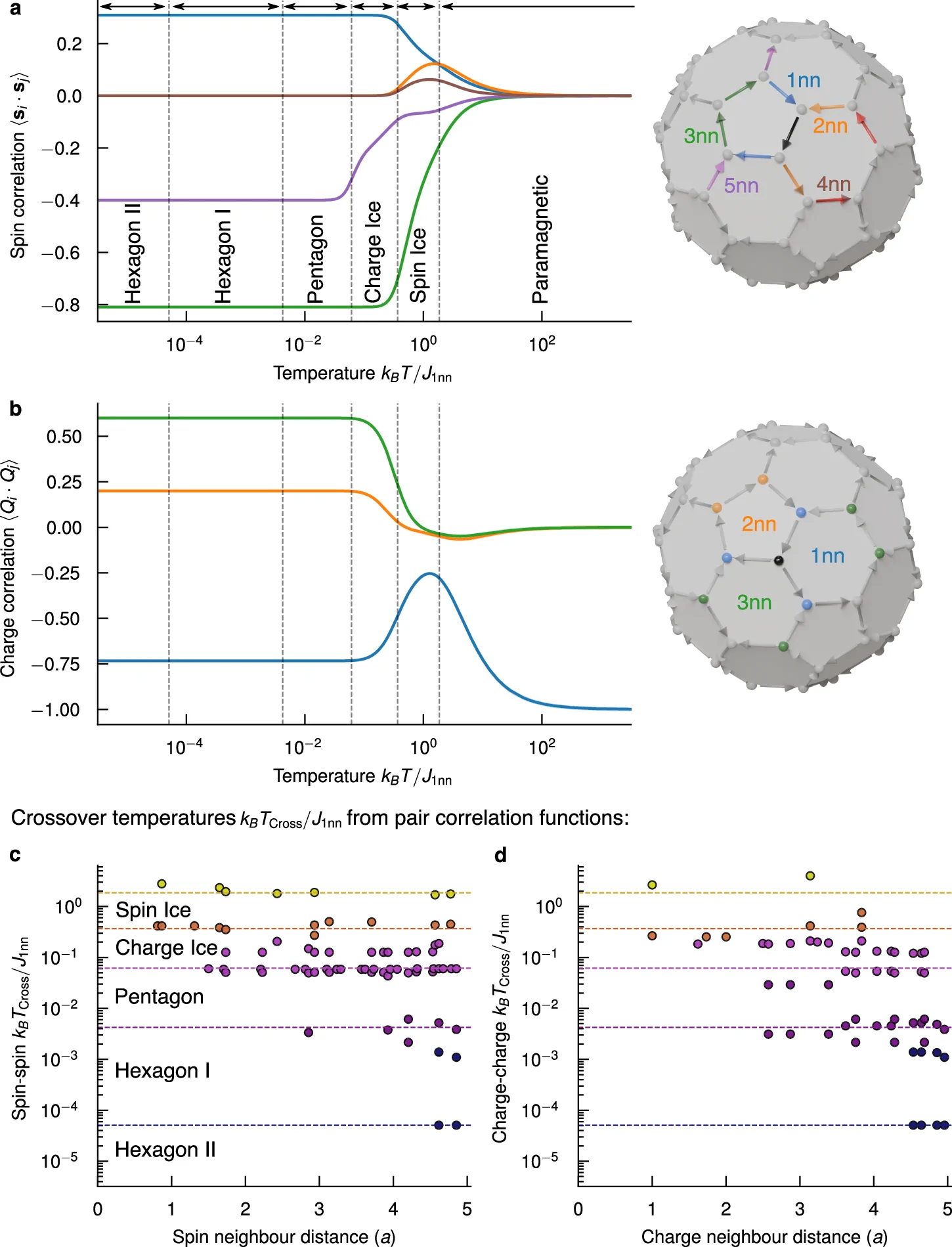
Spin and charge correlations in the buckyball artificial spin ice. **a**, **b** Temperature dependence of the first five nearest-neighbour spin correlations and first three nearest-neighbour charge correlations. In each case, the definitions of the spin or charge neighbours, with respect to the central black spin or vertex respectively, are given to the right. The vertical dashed lines indicate the locations of the five crossover temperatures for the buckyball artificial spin ice. In terms of the spin correlations, first- and second-nearest-neighbour interactions together enforce the existence of an ice rule at each vertex. As a result of the topology of the buckyball, perfect charge order cannot be accommodated, so that the value for ${\langle {Q}_{i}{Q}_{j}\rangle }_{{{{\rm{1nn}}}}}$ pairs (blue curve, **b**) reaches a low temperature value of  − 11/15, as opposed to  − 1 for the artificial kagome spin ice^58^. Error bars are smaller than the line width. **c**, **d** A crossover temperature ${T}_{{{{\rm{Cross}}}}}^{(n)}$ can be assigned to each spin or charge pair by finding the temperature at which the corresponding correlation function has a point of inflection (as described in the Methods). These crossover temperatures are shown as a function of the spin and charge neighbour distance in (**c**, **d**), respectively. Each individual crossover temperature is coloured according to the nearest system-wide crossover, matching the colour of the relevant horizontal dashed line. For the most part, the pairwise crossover temperatures lie close to one of the five system-wide crossover temperatures allowing us to determine which interactions are responsible for the emergence of each sector. It is apparent from these plots that long-range interactions are responsible for the low-temperature peaks, since data points above a neighbour separation of 2.5*a* tend to cluster near to system-wide crossovers to the Pentagon, Hexagon I and Hexagon II sectors as the buckyball artificial spin ice is cooled.

The spin correlations 〈**s**_*i*_ ⋅ **s**_*j*_〉, up to the fifth-nearest-neighbour spin pair, and the charge correlations 〈*Q*_*i*_*Q*_*j*_〉, up to the third-nearest-neighbour charge pair, are displayed as function of temperature in Fig. 3a, b, respectively. The definitions of these nearest-neighbour pairs are given by the associated schematics. The dashed vertical lines indicate the locations of the five peaks observed in the specific heat capacity of the buckyball artificial spin ice shown in Fig. 1c.

From the behaviour of these correlations, we note the following three key points:

1. The interactions between first- and second-nearest-neighbour spins, which completely encompass all interactions at a single vertex, are responsible for the emergence of the Spin-Ice sector on cooling. Here, the corresponding spin correlations have maxima or inflection points at, or near to, the crossover to the Spin-Ice sector, as shown in Fig. 3a. Signatures of the crossover to the Spin-Ice sector can also be seen in the behaviour of other spin correlations, in particular those for 4nn and 5nn spin pairs. This reflects the fact that there needs to be some cooperation between spins at neighbouring vertices to enforce an ice-rule everywhere, since setting a two-out/one-in or two-in/one-out configuration on one vertex necessarily constrains the possible ice-rule configurations on adjacent vertices. In fact, the situation is slightly more nuanced due to the nonequivalent nearest-neighbour interactions at a vertex, which lift the degeneracy of ice-rule states. Evidence of this is found in the temperature dependence of the three vertex populationslow energy *Q* = ± 1 Type 1A and Type 1B, and high energy *Q* = ± 3 Type 2which are shown in the Supplementary Note 4. There it can be seen that, in the Spin-Ice sector, high-energy vertices are no longer present and all vertices obey the ice rule. On cooling from the Spin-Ice sector to the Charge-Ice sector, the higher-energy Type 1B vertices also disappear, and only the four degenerate lowest-energy Type 1A vertices are found.
2. As a result of the topology of the buckyball, perfect charge order cannot be accommodated. Beginning in the Charge-Ice sector, and maintained throughout the remaining low-temperature sectors, some *Q* = + 1 vertices exist that are not surrounded by three *Q* = − 1 vertices, and vice versa. As a result, the value for ${\langle {Q}_{i}{Q}_{j}\rangle }_{{{{\rm{1nn}}}}}$ pairs reaches a low-temperature value of only  − 11/15, as opposed to the low-temperature value of  − 1 for the artificial kagome spin ice. This highlights how the buckyball artificial spin ice is an extremely frustrated system; not only is one of the interactions at each ice-rule-obeying vertex configuration unsatisfied, but the topology of the buckyball means that the vertices themselves cannot be split into bipartite sets. There is then a frustration that arises on a larger length scale, namely between vertices, as a consequence of the imperfect arrangement of the charges.
3. For the subset of spin and charge correlations that we choose to display in Fig. 3a,b, there is little change in the correlation curves in the vicinity of the crossovers to the Pentagon, Hexagon I, or Hexagon II sectors. This suggests that correlations between pairs of spins and charges that are further away from each other must be responsible for the existence of these low-temperature peaks in the specific heat capacity (Fig. 1c).

To clarify the third observation, we assign individual crossover temperatures to each of the nearest-neighbour spin and charge correlation pairs, by finding the temperatures at which the corresponding correlation curves have a point of inflection (see Methods for more details). For the case of two spins, which are *n*th-nearest neighbours separated by a distance *r*_*n*_, we can assign a crossover temperature ${T}_{{{{\rm{Cross}}}}}^{(n)}$, which is the temperature at which fluctuations between *n*th-nearest neighbours are at a local extremum. As the temperature is lowered, the correlations change in size until, at their lowest crossover temperature, the spins freeze, resulting in a constant spin correlation between them. At this point, the temperature scale is such that this interaction plays no further role in the ordering. A similar logic applies to a pair of neighbouring charges.

The crossover temperatures for these individual spin and charge correlators, plotted as a function of the neighbour distance, are displayed in Fig. 3c, d, respectively. Here, the horizontal dashed lines indicate the five crossover temperatures of the buckyball artificial spin ice as a whole. The majority of the lowest spin-spin crossover temperatures lie close to the crossover to the Pentagon sector from the Charge-Ice sector, which is the first temperature at which some form of system-spanning spin order—with all pentagons displaying loops of spins in the same sense—is established. However, there are a number of further-neighbour spin-spin crossover temperatures in the vicinity of the two lowest-temperature crossovers. In terms of the charge-charge crossover temperatures, many of these lie near to the transition to the Charge-Ice sector from the Spin-Ice sector, as expected since this is the point at which charge order is established on the buckyball surface. Beyond a separation of 2.5*a*, many charge-charge interactions have a lowest ${T}_{{{{\rm{Cross}}}}}^{(n)}$ close to the Hexagon I or even Hexagon II transition. We have thus established that, rather than the nearest-neighbour interactions, it is the long-range interactions that are responsible for the three low-temperature crossovers. This is not the complete picture because, as we now demonstrate, the low-temperature magnetic structure is additionally constrained by the geometry of our curved finite system, which means that further-out neighbours are closer than they would be in a 2D artificial spin ice.

### Three-stage spin ordering and ground state motifs

We now show precisely how spin order is progressively established in each of the Pentagon, Hexagon I and Hexagon II sectors. For this, in analogy with the total magnetisation of a magnetic solid, we consider the temperature dependence of 〈**s**_*i*_〉, whose absolute value encodes the average length of the spin vector **s**_*i*_ averaged over the number of Monte Carlo steps performed at that temperature *T*. Then, spin *i* can be said to be frozen when 〈**s**_*i*_〉 = ± 1.

**Fig. 4:**
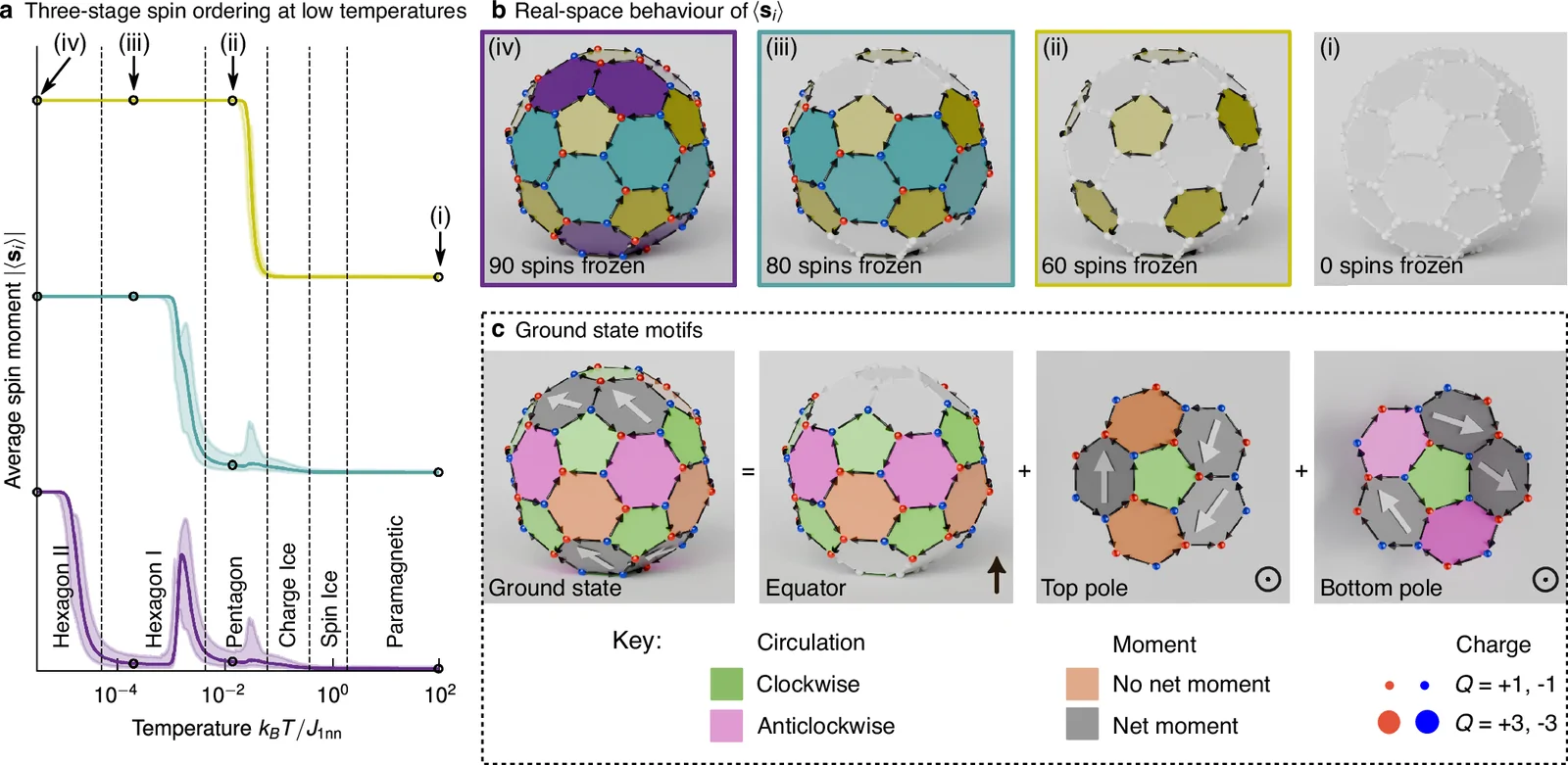
Three-stage spin ordering in the buckyball artificial spin ice. **a** The temperature dependence of the absolute value of the average spin moment 〈**s**_*i*_〉. The 90 spins in the buckyball are sorted into three groups, which order separately in the Pentagon, Hexagon I, and Hexagon II sectors, and are shown by the three curves in gold, turquoise and indigo, with each curve displaced in the *y*-direction. Each curve begins at 0 in the high-temperature limit and reaches 1 by the lowest temperature simulated. For each group, shaded regions indicate the 95% confidence interval around the mean of 5000 independent runs. Uncertainties are calculated separately for data above and below the mean, giving asymmetric error regions, which ensures that the mean and its uncertainty does not go above one. **b** Real-space representations of the average spin 〈**s**_*i*_〉 on each edge of the buckyball, corresponding to the temperatures indicated in panel **a** with labels and circular markers. The faces of the buckyball are coloured according to the temperature at which all their associated spins freeze, matching the colours of panel **a**. The spins appear in black only once they have frozen; otherwise, they appear as white, double-headed arrows to indicate that they continue to fluctuate. The states are: (i) at high temperature, when no spins have ordered; (ii) just below the crossover to the Pentagon sector, when the 60 spins associated with the pentagonal faces have ordered (faces coloured gold); (iii) below the crossover to the Hexagon I sector, when a further 20 spins have ordered so that a belt of spins associated with the hexagons is now frozen (faces coloured turquoise); and (iv) below the crossover to the Hexagon II sector, when the remaining 10 spins have ordered, so that the spins associated with five hexagonal faces either side of the belt freeze (faces coloured indigo). **c** The ground state of the buckyball artificial spin ice can be decomposed into three motifs: an equatorial belt, formed from the maximal number of possible closed-loop pentagons and hexagons; and two poles, top and bottom, which are formed around a central closed-loop pentagon. In this panel, the faces are coloured according to the circulation of their associated spins as given by the key, which matches the colour scheme used in Fig. 2. For those hexagons with a net moment, coloured in grey, a white arrow is included to indicate the net moment direction. In the equator sub-panel, the black arrow points from the bottom pole to the top pole, along the line connecting the polar pentagons. In the pole sub-panels, we adopt a viewpoint looking down on the buckyball artificial spin ice from above the top pole; accordingly, the direction of the black arrow is indicated by a circled dot.

On plotting the absolute value of this quantity for each spin in the buckyball artificial spin ice in Fig. 4a, we observe that the spins fall into three groups (upper, middle and lower curves in gold, turquoise, and indigo, respectively), which freeze at different temperatures near to the low-temperature crossovers. By freezing, we mean that there is usually a sharp increase in ∣〈**s**_*i*_〉∣ from 0 above the freezing temperature to 1 below the freezing temperature. To illustrate better what these groups of spins represent, we show the real-space configurations of 〈**s**_*i*_〉 in Fig. 4b corresponding to the four temperatures indicated with black arrows in panel Fig. 4a. Here, the spins appear in black only when they have frozen; otherwise, they are drawn as white double-headed arrows indicating that they continue to fluctuate. The faces of the buckyball artificial spin ice are coloured accorded to the lowest ordering temperature of their associated spins, in a colour scheme consistent with the three groups of spins in Fig. 4a. The highlighted states are:

(i) at high temperature, when no spins have ordered so that none of the faces are coloured.
(ii) below the crossover to the Pentagon sector, when the 60 spins associated with all 12 pentagons freeze in closed loops with the same sense of circulation (faces coloured gold).
(iii) below the crossover to the Hexagon I sector, when a further 20 spins freeze. These spins are associated with 10 hexagonal faces, shown in turquoise, which form a chain round the equator of the buckyball.
(iv) below the crossover to the Hexagon II sector, when the remaining 10 spins freeze. At this point, the final 10 hexagonal faces—five on either side of the buckyball at the poles—now order, as shaded in indigo.

For comparison, when loop moves are not used during the Monte Carlo simulations, we find that, instead of freezing in three groups as above, the spins freeze in two groups (Supplementary Note 5).

Now we describe the spin motifs that together make up the ground state of the buckyball artificial spin ice (Fig. 4c). This ground state is formed from a belt of spins around the equator, which features the maximal number of flux-closed faces. In this belt, the loops associated with the pentagons and the hexagons have opposite sense; in this example, clockwise and anticlockwise (in green and pink), respectively. Not every hexagon flux-closes on account of the topology of the buckyball. Hence, there are some hexagonal faces that are not flux-closed, which nevertheless have no overall net moment (shaded in orange).

This leaves 10 hexagonal faces, five on either side of the buckyball. Each set of five hexagonal faces surround a central flux-closed pentagon. These two ‘poles’ of the buckyball artificial spin ice involve some hexagonal faces with a net moment. These hexagons are coloured grey and their net moment is indicated with a white arrow. Because of these faces with a net moment, the lattice as a whole retains a small net moment even in the ground state. This net moment of the whole lattice is oriented approximately along the line connecting the centres of the two pentagons associated with each pole, and points towards the top pole. The black arrow in the bottom right of the equator sub-panel of Fig. 4c indicates the axis connecting the bottom and top polar pentagons, directed from bottom to top.

The spin configuration at the poles is locally higher in energy than an arrangement in which the spins associated with the majority of the faces are flux-closed, as occurs around the equator. However, complete flux-closure cannot be accommodated everywhere. These poles are then examples of topological defects in the truest sense: they are constrained by the geometry of the lattice and must form as a consequence of the low-energy flux-closed motif around the equator but, crucially, they cannot be eliminated by a smooth deformation or macrospin flips^50^. These constitute the most intriguing instances of topological defects in an artificial spin ice context, since previous examples have relied on physically distorting the lattice at certain positions to promote their formation^51,52^. Each of the topological defects forms around a flux-closed pentagonal face. The locations of the topological defects are not independent from each other; instead, they are arranged on diametrically-opposite pentagons. There are six possible ways to choose two diametrically-opposite pentagons in the buckyball. In total, it is possible to show that there are 480 permutations of the topological defect pairs, each corresponding to a distinct ground state, which results in a residual entropy of *S*_0_ ≈ 0.0685 *k*_*B*_ per spin. This agrees extremely well with the value obtained through numerical integration of the specific heat capacity, *S*_0_ ≈ 0.069(3) *k*_*B*_ per spin, given in Fig. 1d. Different ground states are connected by changes in the spin configuration at one or both poles. These changes can, in turn, require minor rearrangements of the equatorial spins, swapping flux-closed hexagons with hexagons that have neither a net moment nor spin circulation. In Supplementary Note 7, we discuss in detail how these 480 degenerate ground states are related to each other—particularly with respect to the ground state we have shown in Figs. 2a, 4c.

## Conclusion

Our buckyball artificial spin ice exhibits a diverse spectrum of thermal behaviour, characterised by five crossovers that separate six different magnetic sectors, beginning at higher temperatures with a crossover from a Paramagnetic sector to a Spin-Ice sector, and then to an imperfect charge-ordered crystal. Spin order is established via a three-stage mechanism, which is mediated by dipolar interactions between spins on opposite sides of the buckyball. The ground state itself hosts a pair of robust topological defect ‘poles’ that, although locally high in energy, must be present so that flux-closed motifs can form elsewhere. As with the vertex and charge frustration, these defects carry a certain entropy because of their multiple configurations. In addition, the existence of these topological defects means that complete compensation of the magnetic moment is not possible. As already mentioned, the buckyball artificial spin ice possesses a small net moment even in the ground state, which is orientated approximately along the normal to the pentagonal face at the centre of each pole. In terms of applications, one could then envisage using these 3D finite lattices—or, indeed, assemblies of them—as ultra-sensitive 3D magnetic field sensors, with the location of the topological defects depending on the orientation of the external magnetic field.

We have characterized the magnetic ordering that occurs in one example of an Archimedean solid. We envision that further complex thermal behaviour and new types of magnetic order await to be found in different members of the Platonic, Archimedean, and Catalan solid families, since these lattices all involve polygons with different vertex coordinations and effective local curvature.

In effect, these finite lattices present a new form of artificial molecular magnet, where the moments are situated on the edges rather than the vertices of the solid. Indeed, in a buckyball lattice with the spins located on the vertices, as would be found in molecules, the thermal behaviour is much simpler with only a single crossover (Supplementary Note 8). In contrast, the richness of the physics we observe in our buckyball artificial spin ice stems from the formation of loops of spins linking ice-rule-obeying vertex configurations, which are embedded on a lattice with non-trivial topology.

Now that we have a complete understanding of the buckyball, we can extend this analysis to the broader family of fullerenes, of which the buckyball is one of the simplest examples. This would offer the opportunity to study the physics of mobile topological defects. In the buckyball artificial spin ice, the topological defects are confined: once they are formed, they are not able to move, even in the presence of thermal fluctuations, on account of their large size with respect to the finite size of the solid, since each topological defect involves the spins lining six faces, out of a total of 32 faces. For larger fullerenes, we would still expect topological defects to form initially around the pentagons at the poles to accommodate the curvature, but each individual defect may be less strongly coupled to any of its neighbouring defects. As a result, the defects should be able to depin from the pentagons and move across the lattice at finite temperature. As we continue to increase the size of the fullerenes, we would expect the specific residual entropy to tend to zero, since the limiting case of such lattices is the artificial kagome spin ice lattice, which does not have an extensive residual entropy^38^.

Our findings illuminate the rich landscape of magnetic phenomena that can emerge from the interplay of curvature, constraints and interactions in finite three-dimensional ‘nanomagnetic molecules’. While the fabrication of such lattices using current lithographic techniques is feasible^22,23,53^, probing their thermodynamics remains a significant challenge, with only the frozen states at surfaces imaged with magnetic force microscopy^25,54,55^ and the magnetic configuration of small structures probed with synchrotron x-ray techniques up to now^27^. Overcoming this hurdle will require further development of advanced imaging methodologies^29^.

## Methods

### Monte Carlo simulations

The Monte Carlo simulations were performed using both single spin flips and loop moves. In this context, a loop is any closed head-to-tail chain of spins. When spins in a loop are flipped simultaneously, the charge at each vertex remains the same. This is because every loop must enter and exit a vertex, which ensures local conservation of charge. Once such loop moves are proposed, they are accepted or rejected according to the Metropolis criterion, just as with single spin flips. This criterion dictates that the proposed move is accepted if it lowers the energy of the system outright. Otherwise, it is accepted with a probability proportional to the Boltzmann factor $\exp (-\Delta E/{k}_{B}T)$, where Δ*E* is the energy difference between the initial state and the proposed state. Loop moves help to efficiently sample the low-energy states of a frustrated system^56,57^, when either the waiting time to access the new state is prohibitively long, or where single spin flips are frozen out completely. This often happens when, for example, constraints arising from the topology of the lattice promote the formation of low-energy motifs that incorporate a number of spins. Loop moves then allow the system to move between these different ‘topological sectors’, such as in the Coulomb phase of real spin ice, whose hallmark is algebraic spin-spin correlations^21,56,57^.

In Supplementary Note 1, we show that loop moves are essential to recover all five peaks in the specific heat capacity of the buckyball artificial spin ice. Below the ice-rule crossover, single spin flips that create high-energy *Q* = ± 3 monopoles become energetically unfavourable, causing a slowdown in the dynamics. This is similar to what has been observed in pyrochlore lattices and 2D artificial kagome spin ice, where loop moves are necessary to overcome spin freezing^16,58,59^.

For convenience, we work in reduced units, and re-scale temperatures in terms of the largest dipolar energy, which is the first-nearest-neighbour interaction strength *J*_1nn_ ≈ 3.1424*D*.

In our Monte Carlo simulations, closed loops of spins are constructed through an iterative process. We begin by initialising an empty list and selecting a random spin in the buckyball lattice. The magnetic moment of this spin points toward one vertex and away from another. We move to the vertex it points to and examine the two remaining neighbouring spins at this site. There are three possible scenarios for the two remaining spins:

- If both spins point away from the vertex, one is chosen at random and added to the list.
- If only one spin points away from the vertex, it is selected by default.
- If neither spin points away from the vertex, the process terminates.

This procedure is repeated iteratively for each newly added spin. A closed loop is identified when a spin is revisited within the list. The subset of spins forming the closed loop is then proposed as part of the Monte Carlo loop move. This algorithm, originally introduced by Barkema and Newman for square ice models^45^, appears to satisfy both detailed balance and ergodicity, ensuring accurate sampling of the thermal properties of the system.

The specific heat capacity is calculated by recording the fluctuations in energy about the mean value 〈*E*〉 through

2 $${c}_{{{{\rm{V}}}}}(T)=\frac{1}{N}\frac{\langle {(E-\langle E\rangle )}^{2}\rangle }{{k}_{B}{T}^{2}},$$

where *N* = 90 is the number of spins in the buckyball, which has total energy *E* at temperature *T*. The entropy per spin at temperature *T* is obtained by numerical integration according to

3 $$s(T)={k}_{B} \, {{{\mathrm{ln}}}} \, (2)-{\int }_{T}^{\infty }\frac{{c}_{{{{\rm{V}}}}}}{T^{\prime} }{{{\rm{d}}}}T^{\prime} .$$

The first term on the right hand side, ${k}_{B}\,\ln(2)$, is the appropriate entropy in the high-temperature limit for a spin with an Ising degree of freedom, i.e. with two equally-likely states.

Verification of the Monte Carlo simulations is given in the Supplementary Notes 1–3. This includes: (i) tests on the robustness of the equilibration; (ii) proof of the necessity to include loop moves to recover the full spectrum of thermal behaviour; and (iii) determination of the required number of such moves to the reach the ground state. For the simulation results presented in the main text, the full dipolar sum given in Eq. (1) is implemented. In Supplementary Note 2, we discuss the effect of implementing a cutoff radius beyond which interactions are neglected. We find that truncating the dipolar interaction has the strongest effect on the temperatures, and even the existence, of the three low-temperature crossovers. Indeed, the five peaks in the specific heat capacity are only recovered when all interactions are included because the lattice is 3D and of limited size.

### Assigning crossover temperatures

In Section “Role of long-range interactions in promoting the low-temperature crossovers”, we discussed the allocation of crossover temperatures to individual spin and charge correlations. This approach is analogous to the way in which phase transitions correspond to changes in the curvature of the internal energy *U* that, in turn, can appear as local maxima in *C*_V_ = d*U*/d*T*. For the spin correlations between *n*th-nearest neighbours, ${\langle {{{{\bf{s}}}}}_{i}\cdot {{{{\bf{s}}}}}_{j}\rangle }^{(n)}$, we define the crossover temperature ${T}_{{{{\rm{Cross}}}}}^{(n)}$ as the temperature for which the quantity,

4 $$\frac{{{{\rm{d}}}}}{{{{\rm{d}}}}T}{\langle {{{{\bf{s}}}}}_{i}\cdot {{{{\bf{s}}}}}_{j}\rangle }^{(n)},$$

has a local maximum or minimum. In practise, we do this through numerically solving

5 $$\frac{{{{{\rm{d}}}}}^{2}}{{{{\rm{d}}}}{T}^{2}}{\langle {{{{\bf{s}}}}}_{i}\cdot {{{{\bf{s}}}}}_{j}\rangle }^{(n)}=0$$

for *T*, and checking that the second derivative changes sign around that temperature. In general, a given correlation curve may have more than one such point of inflection.

## Supplementary information

Transparent Peer Review file

Supplemental Material

Description of Additional Supplementary Files

Supplementary Movie 1

Supplementary Movie 2

Supplementary Movie 3

Supplementary Movie 4

Supplementary Movie 5

Supplementary Movie 6

## Data Availability

The data underpinning the results in this paper are available at the Zenodo repository: https://doi.org/10.5281/zenodo.18319213. Supplementary Movies 1–6 are included separately and illustrate the configurations in each of the six sectors shown in Fig. 2.

## Code availability

The Monte Carlo code, written in Julia, is available from an online repository: https://github.com/gmacauley/PolyhedralASI.jl. Further information can be requested from gm3291@princeton.edu.
