# Supplementary material for: Five-stage ordering to a topological-defect-mediated ground state in a buckyball artificial spin ice
Source: Commun Phys. 2026 May 26;9(1):288. doi: 10.1038/s42005-026-02663-y (PMC13549942; doi:10.1038/s42005-026-02663-y)
Supplement: Supplementary file 1 [file 42005_2026_2663_MOESM1_ESM.pdf]

nature portfolio

Peer Review File

Five-stage ordering to a topological-defect-mediated ground state in a buckyball artificial spin ice

Corresponding Author: Dr Gavin Macauley

This file contains all reviewer reports in order by version, followed by all author rebuttals in order by version.

Version 0:

Reviewer comments:

Reviewer #1

(Remarks to the Author)
The authors present a numerical study of magnetic nanowires arranged in a buckyball structure, and reveal a series of distinct phases characterised by flux closure loops and charge ordering. The work is timely considering recent publications on 3D artificial spin ice in this journal, and other high-impact articles on these structures published elsewhere. With the supplementary information, the manuscript is thorough and convincing, leaving only a few minor points for the author's consideration to improve clarity before publication. .

Figure 4 appears to indicate that at the 'hexagon-I' phase, each pole may have three to five faces with some net moment. If my interpretation is correct, clarity could be improved by showing a configuration with either four or five grey faces at the poles to distinguish from the configuration currently shown in figure 2a, which is the same as the one shown in figure 2a but rotated by ~145 degrees.

Presenting 3D systems on a 2D medium is always a challenge, and the figures generally excel at overcoming this challenge. The bottom row of figure 2 shows a net representation of the buckyball, perhaps exploring either a polyhedral graph representation or an azimuthal equidistant ion may improve clarity.

There is some discussion around the use of loop moves in the MC simulation. It would be beneficial if the authors highlight the method used for identifying loops. Presumably, the authors used the Barkema-Newman algorithms as the relevant article appears in the references. However, there is no mention of this in the article or supplementary information.

Reviewer #2

(Remarks to the Author)
The authors studied the Ising model defined on edges of a regular buckyball, where spins interact with each other through the dipolar interactions.
They consider this system as a model of future three-dimensional artificial spin ice.
The authors used Monte Carlo simulation supplemented by a global loop update to obtain the heat capacity and the entropy of the system.
These thermodynamic quantities clearly show the presence of six different sectors of the spin states, depending on the temperature.
In addition to the spin ice and the charge ice sectors, which have analogues in the standard two-dimensional artificial spin ice, they also found three further-constrained states, which they named Pentagon, Hexagon I and Hexagon II.
They characterized these new states with circulation of spins around polygons, inflection points of the spin/charge correlations and the relaxation of spins.
In particular, they identified the lowest-temperature sector as the ground state, which is unique up to symmetry operation, and its degeneracy is consistent with the residual entropy obtained by the Monte Carlo simulation.

Artificial spin ice (ASI) is one of the active areas of spintronics, where lots of crucial scientific notions have been discovered and examined.
While most systems have two-dimensional architecture, an extension to three dimension may be realized with further

development of fabrication technique.
The value of this work solely depends on whether three-dimensional ASI is actually realized in an architecture where the insight from this buckyball buckyball system can be useful.
The authors should supply a convincing reason that the study of this system is likely to be beneficial in this light.

The manuscript is very well written. The authors clarify all aspects of their analyses and give reasonable interpretation of the results.
For this reason, I basically recommend the publication of this article in Communications Physics.
However, before publication, I recommend addressing a few points listed below.

[1] Typicality of the model
three dimensional ASI is a quite promising field, but its standard architecture is not fixed.
If the present system is actually realized as a three-dimensional ASI, this calculation will serve as a pioneering work, otherwise the present study will become just one special case study.
The authors should give enough justification why this buckyball system will supply useful knowledge in this area, once 3D ASI is actually realized.

[2] Role of loop update
The authors insist on the importance of loop update on the equilibration below the temperature of charge ice.
In the case of pyrochlore lattice Ising model, loop update accelerates the dynamics, since the single spin flip creates high-energy monopoles.
In the present system, the origin of energy barrier is less clear.
For example, is the collective spin flip around a single hexagon enough to equilibrate the system ?
Or, is it necessary to consider many different types of the loop ?

[3] Characterization by dynamics
Compared with other analyses presented in this paper, the evaluation of spin freezing in Section 5 seems to have ambiguity.
The expectation value of single spin depends on the number of Monte Carlo steps and more than that, the type of dynamics.
How are the spin freezing patterns in Figure 4 changed by turning on/off the loop update, for example ?

Reviewer #3

(Remarks to the Author)
The manuscript by Macauley et al studies the possible phases in a buckyball artificial spin ice, where Ising degrees of freedom on the edges of the buckyball interact with each other through a dipolar Hamiltonian. Using extensive Monte Carlo simulations, the authors identify six distinct phases (sectors), which are separated by five thermal crossovers as seen in the heat capacity and the entropy, ranging from the high-temperature paramagnet to an ordered ground state with a pair of topological defects. Detailed descriptions of each sector are given in terms of the spins on the edges, the charges on the vertices, the circulations and the net moments around the faces of the buckyball. Potential experimental realization and technological application are also discussed. The subject under study is novel and nontrivial, and the results, backed by rigorous numerical calculations and careful analyses of data, are scientifically sound. The presentation is clear, logical and well organized, which makes the manuscript not only accessible but also pleasant to read. I especially appreciate the efforts spent on creating the graphics, which greatly aid the readers in visualizing and understanding the physics of the buckyball artificial spin ice. I strongly believe the manuscript is of interest to physicists working on artificial spin ices as well as the larger frustrated magnetism community, and it will motivate future studies on similar models defined on finite systems beyond the planar geometry. I thus recommend the publication of the manuscript in Communication Physics, after the authors address several minor issues listed below.

1. When I read the main text, I was expecting to see the Hamiltonian somewhere in the first few sections. It only appears as Eq. (1) under 'Methods', which is after 'Conclusion' in Sec. 6. For this paper, I think there is no problem for not showing the Hamiltonian in the main text, but perhaps the authors can explicitly refer to it e.g. in Sec. 2? A good place to do this is line 109: "The Hamiltonian and further details concerning the Monte Carlo simulations are given in the Methods section and in the Supplementary Information." This will be useful for non-experts who are not familiar with artificial spin ice.

2. Throughout the paper, the heat capacity per spin is expressed in units of the Boltzmann constant k_B, while the entropy per spin is dimensionless. The authors state in Sec. S1 of the Supplementary Information that "Temperatures are thus quoted in units of k_B*T/J_{1nn}. In a similar way, the units of specific heat capacity and specific entropy are then 1/(k_B spin) and 1/spin, respectively." I don't quite understand why the units of entropy is different from heat capacity. For example, according to the integral in Eq. (S2), one should have [s]=[c_V]+[T]-[T]=[c_V] by dimensional analysis, regardless of what units are used for the temperature. Another way to see this is to consider the configurational entropy s=k_B*log(\Omega), where \Omega is the multiplicity and log(\Omega) is dimensionless. Can the authors clarify this?

3. In Sec. 5, the authors associate the residual entropy obtained numerically with the total number of ground state configurations resulting from symmetry permutations of the pair of topological defects. The author writes "...these topological defects can form in any six different locations around pairs of diametrically-opposite pentagons. By symmetry, it is possible to show that there are 480 permutations of such defect pairs..." There are in total six pairs of diametrically-opposite pentagons in the buckyball, and clearly 6*6=36 is far less than the 480 permutations stated by the authors. I would appreciate if the authors can provide a more detailed explanation for the counting e.g. in the Supplemental Information.

4. In line 181, the authors write "...are are not associated with..." Please remove the duplicate "are".

5. The authors should use a uniform notation k_B for the Boltzmann constant. In Fig. 1c and line 366, please change k_b (lowercase b) to k_B (uppercase B).

6. In Eqs. (3) and (S2) showing how the entropy is calculated from the heat capacity, shouldn't the upper/lower limit of the integral be infinity/T? Starting from c_V=T*ds/dT, one gets ds=(c_V/T)*dT and then integrates both sides from T'=T to T'=infinity, while ln(2) (possibly multiplied by k_B, see point #2) corresponds to the entropy at infinite temperature.

7. In line 158 of the Supplementary Information, the authors write "...where loop moves are used (blue circles) and where only single spin flips are used (red crosses)." Referring to Fig. S7a, the symbols in the parentheses should be blue crosses and red circles, respectively.

8. In the caption of Fig. S10, the authors write "...Type 2B vertices (orange curve)...". The typo "Type 2B" should be corrected to "Type 1B".

Version 1:

Reviewer comments:

Reviewer #1

(Remarks to the Author)
Thank you for taking our comments on board, I am confident that this will make a nice contribution to the community and the additional animations are wonderful.

Reviewer #3

(Remarks to the Author)
The authors have carefully considered the comments and clarified the issues raised by the referees. They have carried out relevant revisions which further improve the manuscript. The overall presentation is of very high quality. I thus recommend the publication of the manuscript in Communications Physics.

Open Access This Peer Review File is licensed under a Creative Commons Attribution 4.0 International License, which permits use, sharing, adaptation, distribution and reproduction in any medium or format, as long as you give appropriate credit to the original author(s) and the source, provide a link to the Creative Commons license, and indicate if changes were made.
In cases where reviewers are anonymous, credit should be given to 'Anonymous Referee' and the source.

The images or other third party material in this Peer Review File are included in the article's Creative Commons license, unless indicated otherwise in a credit line to the material. If material is not included in the article's Creative Commons license and your intended use is not permitted by statutory regulation or exceeds the permitted use, you will need to obtain permission directly from the copyright holder.
To view a copy of this license, visit https://creativecommons.org/licenses/by/4.0/

Dear Editor, dear Reviewers,

We are grateful to the Reviewers for their positive recommendations regarding our manuscript, “Five-stage ordering to a topological-defect-mediated ground state in a buckyball artificial spin ice”, and for providing extremely valuable feedback, which has helped to improve the clarity of both the text and the figures.

In this author response, we explain point-by-point how we have addressed the comments of the Reviewers in the revised version of the manuscript. We reproduce comments from the Reviewers in green; our responses are shown in black; and any changes to the manuscript are displayed in blue. This document should be read in conjunction with the “diff” version of the manuscript, where all additions and deletions are highlighted explicitly. Figure callouts, line numbers and reference numbers in this document refer to the revised versions of the main text and Supplementary Information. At the end of the of this Reviewer Response, we provide a list of some other minor changes to the text.

Yours sincerely,

Gavin M. Macauley, Luca Berchialla, Peter M. Derlet and Laura J. Heyderman

Reviewer #1 (Remarks to the Author):

The authors present a numerical study of magnetic nanowires arranged in a buckyball structure, and reveal a series of distinct phases characterised by flux closure loops and charge ordering. The work is timely considering recent publications on 3D artificial spin ice in this journal, and other high-impact articles on these structures published elsewhere. With the supplementary information, the manuscript is thorough and convincing, leaving only a few minor points for the author's consideration to improve clarity before publication.

We thank the Reviewer for their positive review of our manuscript.

Figure 4 appears to indicate that at the 'hexagon-I' phase, each pole may have three to five faces with some net moment. If my interpretation is correct, clarity could be improved by showing a configuration with either four or five grey faces at the poles to distinguish from the configuration currently shown in figure 2a, which is the same as the one shown in figure 2a but rotated by ~145 degrees.

Presenting 3D systems on a 2D medium is always a challenge, and the figures generally excel at overcoming this challenge. The bottom row of figure 2 shows a net representation of the buckyball, perhaps exploring either a polyhedral graph representation or an azimuthal equidistant projection may improve clarity.

The Reviewer raises an excellent point regarding the best way to make clear the difference between magnetic configurations in the Hexagon I and Hexagon II sectors. There is no difference in the number of faces with a net moment (coloured grey in figures throughout the main text and Supplementary Information). In both the Hexagon I and Hexagon II sectors, six of the faces have a net moment, as shown by the net representation in Fig. 2**a**,**b**. Rather, what separates these sectors is how the spins comprising the features we call the "poles" are correlated, or, equivalently, how the faces with a net moment on one pole are arranged with respect to the faces with a net moment on the other pole. To make this clear, we have added the following sentence to the main text,

> "The remaining two configurations (Fig. 2**b** and Fig. 2**a**) are taken from the two low-temperature sectors. These two sectors differ in how the hexagonal faces with a net moment are distributed across the surface of the solid, and we have thus named them Hexagon I and Hexagon II. In both of these sectors, six of the hexagonal faces have a net moment. We colour these faces grey according to the key in Fig. 2." [Lines 186 – 190]

The Reviewer suggests using either "a polyhedral graph representation or an azimuthal equidistant projection" to improve clarity compared with the net configurations we show in Fig. 2. The reason we have chosen not to display these in the main text is that both suggested representations distort the lengths of the edges and the size of the faces. This means that some spins appear larger than others, which tends to give them undue prominence. As regards the azimuthal equidistant projection, an extra complication

arises from the fact that the buckyball, with a sphericity of ~0.95, is not quite a perfect sphere. In fact, the midpoints of the edges of a regular buckyball are slightly closer to its centre than are the vertices, which further distorts the representation.

Instead, to aid with interpretation of the real-space configurations, we have now produced Supplementary Movies, which allow the viewer to appreciate the full three-dimensional (3D) magnetic configuration, by rotating the buckyball lattice about the *z*-axis. These have been produced for each of the example configurations in Fig. 2 of the main text. We hope these will help to enhance the clarity of the presentation.

There is some discussion around the use of loop moves in the MC simulation. It would be beneficial if the authors highlight the method used for identifying loops. Presumably, the authors used the Barkema-Newman algorithms as the relevant article appears in the references. However, there is no mention of this in the article or supplementary information.

We thank the Reviewer for bringing this omission to our attention. They are correct that we apply the algorithm of Barkema and Newman. We have now added an additional paragraph to the Methods where we describe in detail how closed loops of spins are constructed in our Monte Carlo simulations. This paragraph reads:

> "In our Monte Carlo simulations, closed loops of spins are constructed through an iterative process. We begin by initialising an empty list and selecting a random spin in the buckyball lattice. The magnetic moment of this spin points toward one vertex and away from another. We move to the vertex it points to and examine the two remaining neighbouring spins at this site. There are three scenarios:
>
> 1. If both spins point away from the vertex, one is chosen at random and added to the list.
> 2. If only one spin points away from the vertex, it is selected by default.
> 3. If neither spin points away from the vertex, the process terminates.
>
> This procedure is repeated iteratively for each newly added spin. A closed loop is identified when a spin is revisited within the list. The subset of spins forming the closed loop is then proposed as part of the Monte Carlo loop move. This algorithm, originally introduced by Barkema and Newman in Ref. [46] for square ice models, satisfies both detailed balance and ergodicity, ensuring accurate sampling of the thermal properties of the system." [Lines 391 – 402]

For completeness, Ref. 46 is:

[46] Barkema, G. T. & Newman, M. E. J. Monte Carlo simulation of ice models. Phys. Rev. E **57**,1155–1166 (1998)

Please see also those answers to Reviewer #2, which pertain to loop moves.

We thank the Reviewer for their assistance in improving our manuscript.

Reviewer #2 (Remarks to the Author):

The authors studied the Ising model defined on edges of a regular buckyball, where spins interact with each other through the dipolar interactions. They consider this system as a model of future three-dimensional artificial spin ice. The authors used Monte Carlo simulation supplemented by a global loop update to obtain the heat capacity and the entropy of the system. These thermodynamic quantities clearly show the presence of six different sectors of the spin states, depending on the temperature. In addition to the spin ice and the charge ice sectors, which have analogues in the standard two-dimensional artificial spin ice, they also found three further-constrained states, which they named Pentagon, Hexagon I and Hexagon II.

They characterized these new states with circulation of spins around polygons, inflection points of the spin/charge correlations and the relaxation of spins. In particular, they identified the lowest-temperature sector as the ground state, which is unique up to symmetry operation, and its degeneracy is consistent with the residual entropy obtained by the Monte Carlo simulation.

Artificial spin ice (ASI) is one of the active areas of spintronics, where lots of crucial scientific notions have been discovered and examined. While most systems have two-dimensional architecture, an extension to three dimension may be realized with further development of fabrication technique. The value of this work solely depends on whether three-dimensional ASI is actually realized in an architecture where the insight from this buckyball buckyball system can be useful. The authors should supply a convincing reason that the study of this system is likely to be beneficial in this light.

We feel this point is closely related to the Reviewer's remarks regarding the "Typicality of the model". Consequently, we answer it below.

The manuscript is very well written. The authors clarify all aspects of their analyses and give reasonable interpretation of the results. For this reason, I basically recommend the publication of this article in Communications Physics. However, before publication, I recommend addressing a few points listed below.

We thank the Reviewer for their recommendation of our manuscript.

[1] Typicality of the model
Three dimensional ASI is a quite promising field, but its standard architecture is not fixed. If the present system is actually realized as a three-dimensional ASI, this calculation will serve as a pioneering work, otherwise the present study will become just one special case study. The authors should give enough justification why this buckyball system will supply useful knowledge in this area, once 3D ASI is actually realized.

The Reviewer is correct that the field of 3D artificial spin ice is still in its infancy, and it remains unclear exactly what form experimental systems will take—whether in terms of the most interesting lattices, in terms of large arrays or finite structures, or in terms of connected or disconnected magnetic structures. This contrasts with two-

dimensional (2D) artificial spin ice, where large arrays of disconnected, stadium-shaped nanomagnets are the standard.

Nevertheless, we believe that the buckyball artificial spin ice should serve as a guiding model for research into 3D artificial spin ice for the following reasons:

1. **Natural extension of the artificial kagome spin ice.**
   The buckyball artificial spin ice is the natural 3D extension of the 2D artificial kagome spin ice. Since the inception of artificial spin ice, there has been a wealth of studies—many of which we reference in our manuscript—where nanomagnets are arranged on the edges of the kagome lattice. Accessing the low-temperature sectors of the phase diagram of the artificial kagome spin ice remains an area of active interest [see, for example, Hofhuis PRB (2020) or Hofhuis Nat. Phys. (2022)]. In our manuscript, we highlight the structural similarities between these two systems when we write,

   "The conventional 2D artificial spin ice geometry that most closely resembles our buckyball lattice is the artificial kagome spin ice. Indeed, the surface of a buckyball bears similarities to a kagome lattice that has been wrapped around on itself to leave no boundaries." [Lines 89 – 91]

   Given the strong interest in artificial kagome spin ice due to its rich phase diagram—and the fact that the thermal properties of the buckyball artificial spin ice are equally as, if not more, intriguing—it is natural to expect that buckyball-like structures will attract significant attention from the artificial spin ice community. This growing interest is already evident from several micromagnetic studies of the magnonic response of buckyball structures, which we cite as Refs. 19 and 20 in the main text.

2. **Magnetic buckyballs have been fabricated already using two-photon lithography.**
   There exist several reports demonstrating the fabrication of mesoscopic magnetic buckyballs by coating a magnetic material onto a polymer scaffold created using two-photon lithography. One of these appears already as Ref. 22 in our manuscript, where atomic layer deposition is used to coat the buckyball scaffold. We have now also added a reference to Pip *et al*. Small (2020), which appears as Ref. 31 and presents electroless deposition of Ni-Fe alloys onto various polymer scaffolds, including buckyballs. In neither Ref. 22 nor Ref. 31, however, is 3D magnetic imaging attempted. Such magnetic imaging should now be possible using soft X-ray magnetic laminography, as demonstrated for the case of tripods by Pip *et al*. APL (2022) (Ref. 27). Were magnetic imaging attempted—and we believe the time is now ripe for such an effort—our buckyball manuscript would serve as a valuable guide for understanding the magnetic state of the system.

To answer this comment of the Reviewer, we have inserted the following sentences into the Introduction to emphasise that the buckyball artificial spin ice should act as an important step in understanding 3D ASI:

> "Such difficulties notwithstanding, the buckyball artificial spin ice that we propose in this manuscript stands out as a particularly promising platform for advancing our understanding of 3D artificial spin ices. Its close relation to the well-studied 2D artificial kagome spin ice suggests that insights gained from 2D systems could provide a valuable foundation for interpreting the magnetic behaviour of buckyball structures. Furthermore, since buckyball lattices have already been fabricated[22,23,30-33] and the relevant imaging methods have been developed[27], successful magnetic imaging should soon be within reach. Imaging will be the crucial next step towards confirming our theoretical predictions about the new types of magnetic order that emerge in the buckyball artificial spin ice."
> [Lines 60 – 67]

We have added the following references to the paper:

- Donnelly, C. Hard X-ray Tomography of Three Dimensional Magnetic Structures. (ETH Zurich, 2017). doi:10.3929/ethz-b-000213985. (Ref. 30)
  Containing additional details to that included in the already-cited Donnelly PRL (2015) (Ref. 22), this thesis contains a chapter on atomic layer deposition of magnetic materials onto a buckyball scaffold, prepared using two-photon lithography.

- Pip, P., Donnelly, C., Döbeli M., Gunderson, C., Heyderman, L. J. & Philippe, L., Electroless Deposition of Ni–Fe Alloys on Scaffolds for 3D Nanomagnetism, *Small* **16**, 44 (2022). (Ref. 31)
  As we note above, this work presents electroless deposition of permalloy onto polymer scaffolds.

- Liu, X. *et al.*, High-resolution 3D nanoprinting based on two-step absorption via an integrated fiber-coupled laser diode. *Optics Letters* **48**, 16 (2023). (Ref. 32)
  This work showcases 3D structures, including buckyballs, made from *two-step* photon absorption (Fig. 3c). Here, the focus is on creating high-resolution scaffolds, rather than coating them with magnetic material.

- Wang, Y., Yi, C., Tian, W., Liu, F. & Cheng, G. J., Free-space direct nanoscale 3D printing of metals and alloys enabled by two-photon decomposition and ultrafast optical trapping. *Nat. Mater.* **23**, 1645–1653 (2024). (Ref. 33)
  This work, which appeared while our paper was under review, discusses two-photon decomposition of metal atoms from volatile precursors, enabling the direct printing of 3D metal structures without relying on polymer scaffolds. Notably, Fig. 3b shows SEM micrographs of buckyball-like structures. Although magnetic precursors were not used, this demonstrates a promising alternative route to fabricating to a buckyball artificial spin ice.

These additional references underscore the growing interest in creating artificial magnetic buckyballs, whose magnetic states can compared to those predicted by our work.

[2] Role of loop update

The authors insist on the importance of loop update on the equilibration below the temperature of charge ice. In the case of pyrochlore lattice Ising model, loop update accelerates the dynamics, since the single spin flip creates high-energy monopoles. In the present system, the origin of energy barrier is less clear.

The Reviewer raises an interesting point about the role of energy barriers in the buckyball artificial spin ice.

All vertices in buckyball artificial spin ice carry a magnetic charge: $Q = \pm1$ for both types of ice-rule vertices, and $Q = \pm3$ for high-energy vertices. We would emphasise that a high-energy $Q = \pm3$ monopole can be created by flipping a single spin in an ice-rule vertex, similar to the creation of high-energy monopoles from a charge-neutral background in pyrochlore lattices, as noted by the Reviewer. Like the pyrochlore lattice, this suggests that certain single spin flips in the buckyball artificial spin ice will become energetically unfavorable once the system undergoes the crossover from the Paramagnetic sector to the Spin-Ice sector, leading to a slowing down in the dynamics.

This spin freezing effect has already been observed in both experimental and numerical studies of the 2D artificial kagome spin ice, e.g. Refs. 51 and 60 in the main text. Please note that Ref. 60 has been newly-added since it contains a discussion of the fact that the artificial kagome spin ice requires loop moves precisely because it forms a densely-packed loop sector.

To respond to this comment of the Reviewer, we have added the following sentences to the Methods section of the main text:

> "In Section I of the Supplementary Information, we show that loop moves are essential to recover all five peaks in the heat capacity of the buckyball artificial spin ice. Below the ice-rule crossover, single spin flips that create high-energy $Q = \pm3$ monopoles become energetically unfavourable, causing a slowdown in the dynamics. This is similar to what has been observed in pyrochlore lattices and 2D artificial kagome spin ice, where loop moves are necessary to overcome spin freezing[16,51,60]." [Lines 384 – 388]

Here, the references are:

[16] Hofhuis, K. *et al.* Real-space imaging of phase transitions in bridged artificial kagome spin ice. Nat. Phys. **18**, 699–705 (2022).

[51] Hofhuis, K. *et al.* Thermally superactive artificial kagome spin ice structures obtained with the interfacial Dzyaloshinskii-Moriya interaction. Phys. Rev. B **102**, 180405 (2020).

[60] Schánilec, V. *et al*. Bypassing Dynamical Freezing in Artificial Kagome Ice. Phys. Rev. Lett. **125**, 057203 (2020).

For example, is the collective spin flip around a single hexagon enough to equilibrate the system ? Or, is it necessary to consider many different types of the loop ?

This is a fascinating question, which we had not considered. To address it, we have added a new figure to the Supplementary Information (Fig. S3), where we compare the specific heat capacity as a function of temperature for the following scenarios: (i) no loop moves (red); (ii) loop moves of any length (blue); (iii) loop moves involving only flux-closed pentagons (yellow); and (iv) loop moves involving only flux-closed hexagons (green).

Surprisingly, the most important loop moves are those involving spins around pentagonal faces. Only when these pentagonal loop moves are included do we recover all five peaks in the heat capacity. We suggest this is because all pentagons form flux-closed loops, including the central pentagons at the core of the topological defects. In contrast, performing only hexagonal loop moves does not allow transitions between charge states at the defect poles, as many hexagons carry a net moment and cannot be accessed by loop moves.

In the main text, we now point the reader to the results of this supplementary figure as part of a wider discussion about the importance of loop moves in recovering the five peaks in the specific heat capacity of the buckyball artificial spin ice:

> "Remarkably, also, we find that the most important loop moves involve the spins arranged along the pentagonal faces of the buckyball artificial spin ice (Supplementary Information, Section I)." [Lines 152 - 154]

We have reproduced this supplementary figure and its caption below, along with the additional text in the Supplementary Information that explains this feature.

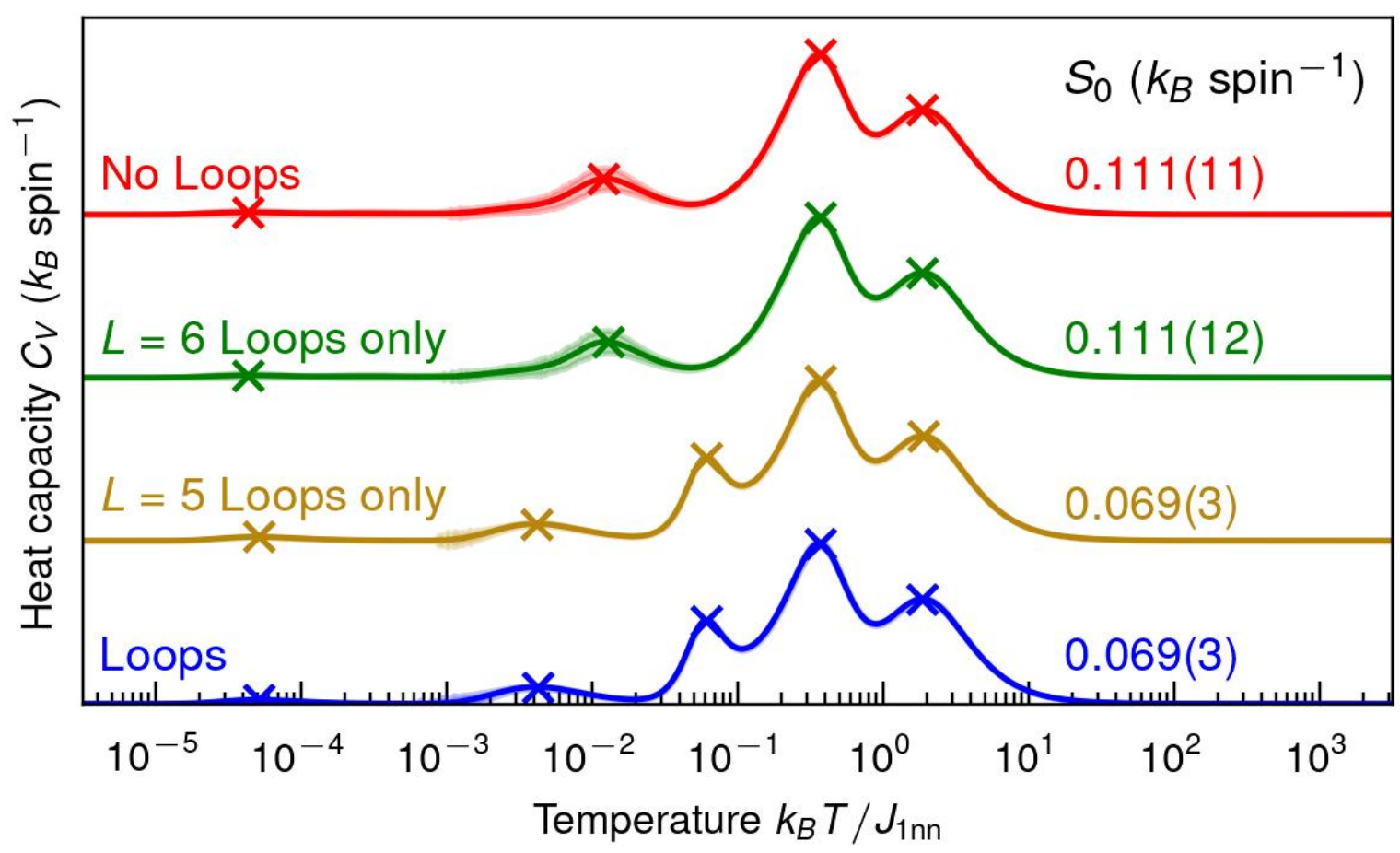

Figure S3 | Effect of allowing only loop moves of a certain length on the specific heat capacity of the buckyball artificial spin ice. The specific heat capacity as a function of temperature is shown for four cases: when loop moves of arbitrary length are allowed (blue); when only loop moves of length L = 5 are allowed, i.e., involving spins on

pentagonal faces (orange); when only loop moves of length $L = 6$ are allowed, i.e., involving spins on hexagonal faces (green); and when no loop moves are attempted (red). The residual entropy for each curve is indicated to the right. We find that it is necessary to include loop moves of length $L = 5$ to reproduce the characteristic five peaks seen in the unrestricted (blue) loop case. The residual entropy is also the same. This indicates that enabling collective flips of spins around flux-closed pentagonal loops is essential for recovering the correct thermal properties of the system.

To accompany this supplementary figure, we have added the following text to the Supplementary Information in Section S1.2:

> "In the main text, we impose no restriction on the loop length L, as long as the spins in the loop form a head-to-tail (flux-closed) configuration. In Fig. S3, we show the impact on the specific heat capacity when loop moves are restricted to a fixed length L, such as loops formed by spins around a single pentagonal face ($L = 5$) or hexagonal face ($L = 6$), together with the cases where either general loop moves are allowed or only spin flips are performed. We find that the characteristic five peaks in the specific heat of the buckyball artificial spin ice are only recovered when loop moves involving spins on pentagonal faces are allowed.
>
> This may seem surprising: we refer to the low-temperature sectors as Hexagon I and Hexagon II because their structure is governed by rearrangements of the hexagonal faces with a net a magnetic moment and, yet, it appears that the spins lining pentagonal faces are more important in accessing this ground state. We justify the importance of loop moves involving pentagonal faces for two reasons. First, among the hexagonal faces that make up each topological defect pole, six carry a net magnetic moment. This means that their spins do not form flux-closed configurations, and are therefore not valid candidates for loop moves. As a result, if only hexagonal loop moves are attempted, those hexagons whose associated spins have a net moment cannot be rearranged. Second, only by flipping the spins on the central pentagon of each topological defect can all five surrounding hexagons be influenced simultaneously. This makes pentagonal loops essential for enabling the collective dynamics that govern the low-temperature thermal behavior of the system. Nevertheless, we continue to refer to the low-temperature sectors as Hexagon I and Hexagon II because, in each case, what defines them is a redistribution of the hexagonal faces with a net moment."

[3] Characterization by dynamics
Compared with other analyses presented in this paper, the evaluation of spin freezing in Section 5 seems to have ambiguity. The expectation value of single spin depends on the number of Monte Carlo steps and more than that, the type of dynamics. How are the spin freezing patterns in Figure 4 changed by turning on/off the loop update, for example ?

The Reviewer has asked another excellent question, which encouraged us to return to the data. We find that, when only single spin flips are used, spin freezing occurs in **two steps** to reach the "No Loops" configuration shown in Fig. S4**b**.

Comparing the simulated anneals with and without loop moves:

- When both single spin flips and loop moves are used, the buckyball artificial spin ice exhibits five crossovers as a function of temperature, with a **three-stage** spin freezing. The residual entropy is then $S_0^{\mathrm{Loops}} = 0.069(3)$ k_B per spin. These are the results we present in the main text.
- When only single flips are used, the buckyball artificial spin ice exhibits four crossovers as a function of temperature, with a **two-stage** spin freezing. The residual entropy is higher, with $S_0^{\mathrm{No\ Loops}} = 0.111(11)$ k_B per spin.

To answer this comment of the Reviewer, we have added the following section to the Supplementary Information, **S6 Comparison of Spin Freezing with and without Loop Moves**. In this section, we include a new figure where we present real-space configurations of $\langle \mathbf{s}_i \rangle$ and the average vertex charge $\langle Q_v \rangle$ for each vertex *v* in this buckyball artificial spin ice for the case in which both loop moves and single spin flips are used during the simulated anneal, and for the case in which only single spin flips are used.

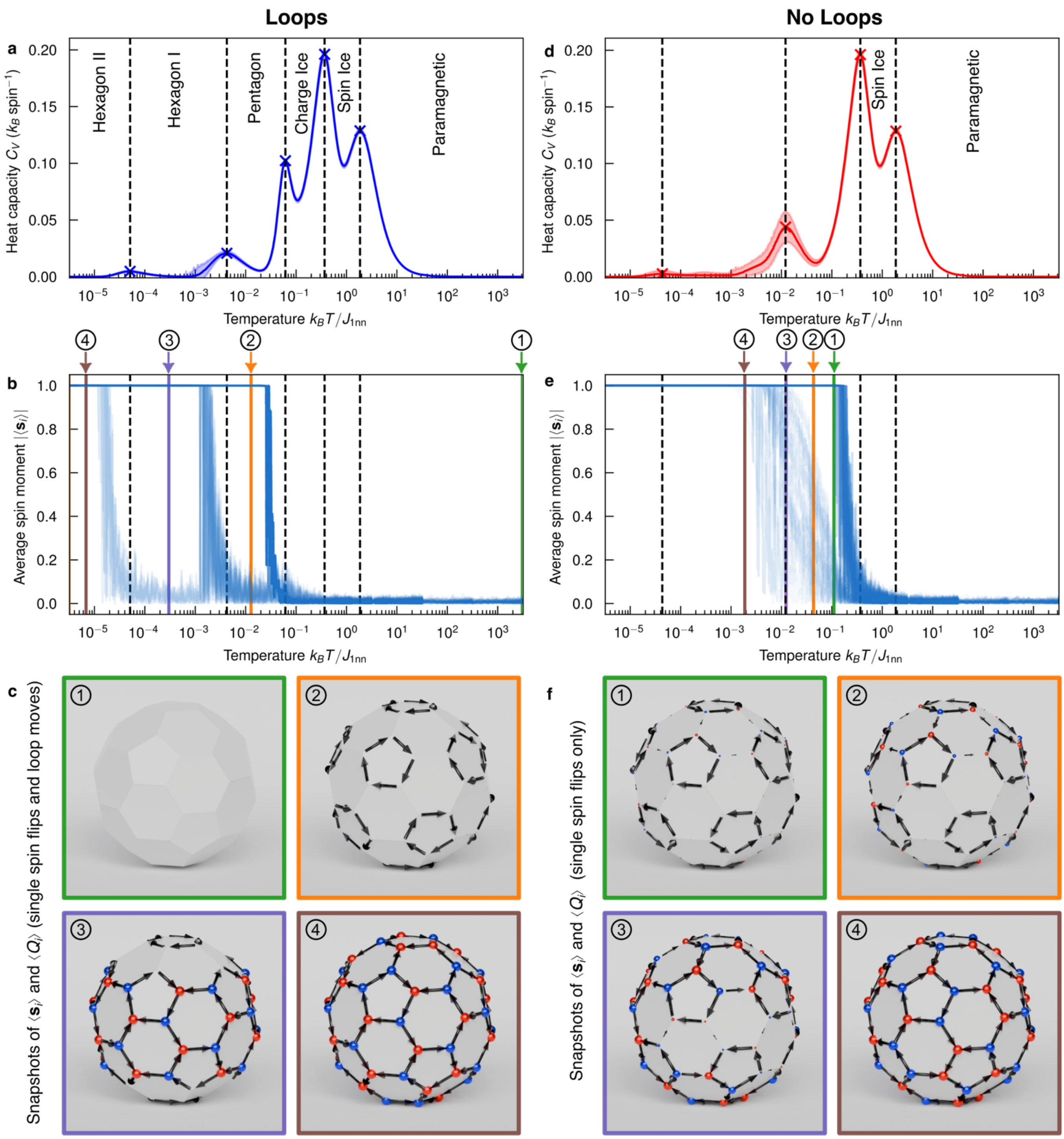

Figure S13 | Effect of loop moves on spin freezing in the buckyball artificial spin ice during simulated annealing. The left column shows results from simulated anneals where both single spin flips and loop moves are allowed (as in Fig. 2 and Fig. 4 of the main text). The right column shows results from anneals using only single spin flips, with loop moves disabled. The top and second rows show **a**,**d**, the temperature dependence of the specific heat capacity, c_V, and **b**,**e**, the average spin moment, $\langle \boldsymbol{s}_i \rangle$ for each spin in the lattice. Without loop moves, the characteristic three-stage spin ordering is no longer

observed (panel **e**). The 60 spins on the 12 pentagonal faces still freeze together. However, the remaining 30 spins freeze gradually over a broad temperature range, beginning around $k_B T/J_{1nn} \approx 10^{-1}$ and completing by $k_B T/J_{1nn} \approx 2 \times 10^{-3}$.

To accompany this figure, we have added the following text to the Supplementary Information:

" S6 Comparison of Spin Freezing with and without Loop Moves

In the main text, we discussed how partial spin order emerges in the buckyball artificial spin ice when certain subsets of spins freeze near to the three low-temperature crossovers. This conclusion was based on examining the expectation value of each spin $\langle \boldsymbol{s}_i \rangle$. This quantity depends on the specific dynamics employed for the Monte Carlo simulations, in particular, whether loop moves are included. This is because loop moves allow collective fluctuations to continue at low temperatures, by which point single spin flips alone are rejected. In Fig. S13, we compare simulations of the spin freezing behaviour reported in the main text—where both single spin flips and loop moves were used—to simulations in which only single spin flips are employed. The left column (labelled 'Loops') displays data from simulations using both single spin flips and loop moves, matching the results presented in the main text. The right column (labelled 'No Loops') displays data from simulations using single spin flips only.

S6.1 Including Single Spin Flips and Loop Moves ('Loops')

As discussed in the main text, when both single spin flips and loop moves are used in Monte Carlo simulations, the specific heat capacity of the buckyball artificial spin ice exhibits five crossovers, as shown in Fig. S13**a**. The temperatures corresponding to these five peaks are indicated by the dashed vertical lines. The curve of specific heat capacity against temperature in Fig. S13**a** is identical to that shown in the Fig. 2**c** of the main text and to the blue curve in Fig. S2**a**.

In this case, the spins in the buckyball artificial spin ice can be divided into three groups, based on the behaviour of $\langle \boldsymbol{s}_i \rangle$. This is illustrated in Fig. S13**b**, where we plot the temperature dependence of $\langle \boldsymbol{s}_i \rangle$ for each spin as a faint blue trace. When many spins behave similarly, the overlapping traces produce a darker colour. In Fig. S13**c**, we show 3D representations of $\langle \boldsymbol{s}_i \rangle$ and the average vertex charge $\langle Q_i \rangle$ at the four temperatures indicated in Fig. S13**b**. In these snapshots, the length of arrow '*i*' is proportional to $\langle \boldsymbol{s}_i \rangle$, and the size of the sphere representing vertex '*i*' is proportional to $\langle Q_i \rangle$. The colour of the sphere represents the sign of the average charge: red (blue) if $\langle Q_i \rangle > 0$ ($\langle Q_i \rangle < 0$). Because we have selected snapshots where some spins are frozen and others are still fluctuating, the spins and charges tend to appear either full-sized or very small. As a reminder, the quantities $\langle \boldsymbol{s}_i \rangle$ and $\langle Q_i \rangle$ denote the time-averaged spin moment and vertex charge, respectively, and are computed by averaging over the number of Monte Carlo steps performed at a given temperature.

In order of decreasing temperature, the four snapshots in Fig. S13**c** are:

- Panel ①, in the Paramagnetic sector, when none of the spins have ordered;
- Panel ②, below the crossover to the Pentagon sector, when the 60 spins around the 12 pentagonal faces freeze into closed loops with the same circulation;
- Panel ③, below the crossover to the Hexagon I sector, when a further 20 spins freeze on 10 hexagonal faces, forming a belt of flux-closed faces around the equator of the buckyball, which features the maximal number of flux-closed faces; and
- Panel ④, below the final crossover to the Hexagon II sector, when the remaining spins freeze in a loop around the central pentagon at each pole.

These snapshots of $\langle \boldsymbol{s}_i \rangle$ and $\langle Q_i \rangle$ are analogous to those presented in Fig. 4**b** of the main text, except that we do not colour the faces.

S6.2 Including Single Spin Flips only ('No Loops')

When the Monte Carlo simulations feature only single spin flips, the specific heat capacity of the buckyball artificial spin ice exhibits four distinct peaks, as seen in Fig. S13**d**. The temperatures corresponding to these four peaks are indicated by the dashed vertical lines. The behavior of the specific heat capacity in the 'No Loops' scenario is discussed in more detail in Section S1.2. Specifically, the curve of specific heat capacity against temperature in Fig. S13**d** is identical to the red curve in Fig. S2**a.** While we have not exhaustively classified the magnetic sectors that emerge with only single spin-flip dynamics, we expect that the two highest-temperature sectors correspond to a Paramagnetic sector and a Spin-Ice sector. This is supported by the fact that the temperature dependence of the specific heat capacity is identical for simulations with and without loop moves down to $k_B T/J_{1nn} \approx 2 \times 10^{-1}$, as shown in Fig. S2**a**.

The behavior of the average spin moment $\langle \boldsymbol{s}_i \rangle$ also differs from the case where loop moves are enabled (see Fig. S13**e**). In the absence of loop moves, the 90 spins in the system separate into two groups with distinct freezing behaviors:

- The first group consists of 60 spins located on the 12 pentagonal faces. These spins freeze over a narrow temperature window centered around $k_B T/J_{1nn} \approx 2 \times 10^{-1}$.
- In the second group, the remaining 30 spins freeze gradually over a broader temperature range, starting near $k_B T/J_{1nn} \approx 10^{-1}$ and finishing by $k_B T/J_{1nn} \approx 2 \times 10^{-3}$.

To illustrate this progressive freezing, we have highlighted four representative temperatures in Fig. S13**e**, with corresponding snapshots of $\langle \boldsymbol{s}_i \rangle$ and $\langle Q_i \rangle$ shown in Fig. S13**f**. In order of decreasing temperature, these snapshots correspond to:

- Panel ①, at $k_B T/J_{1nn} \approx 10^{-1}$, when the 60 spins on the pentagonal faces freeze into closed loops with uniform circulation;
- Panel ②, at $k_B T/J_{1nn} \approx 4 \times 10^{-2}$, where a subset of the remaining 30 spins begin to freeze, resulting in smaller sized arrows in the snapshot;
- Panel ③, at $k_B T/J_{1nn} \approx 1 \times 10^{-2}$, by which point an additional 10 spins have fully frozen; and
- Panel ④, at $k_B T/J_{1nn} \approx 1 \times 10^{-3}$, where all 90 spins have frozen, yielding the configuration shown in Fig. S4**a**, where the faces are coloured to indicate the circulation or net moment of each face.

Similar to the results of the simulations with loop moves, we observe that all spins on the pentagonal faces freeze simultaneously at a common temperature. However, this freezing occurs at a higher temperature when only single flips are used."

We thank the Reviewer for their help in clarifying aspects of our manuscript.

Reviewer #3 (Remarks to the Author):

The manuscript by Macauley et al studies the possible phases in a buckyball artificial spin ice, where Ising degrees of freedom on the edges of the buckyball interact with each other through a dipolar Hamiltonian. Using extensive Monte Carlo simulations, the authors identify six distinct phases (sectors), which are separated by five thermal crossovers as seen in the heat capacity and the entropy, ranging from the high-temperature paramagnet to an ordered ground state with a pair of topological defects. Detailed descriptions of each sector are given in terms of the spins on the edges, the charges on the vertices, the circulations and the net moments around the faces of the buckyball. Potential experimental realization and technological application are also discussed. The subject under study is novel and nontrivial, and the results, backed by rigorous numerical calculations and careful analyses of data, are scientifically sound. The presentation is clear, logical and well organized, which makes the manuscript not only accessible but also pleasant to read. I especially appreciate the efforts spent on creating the graphics, which greatly aid the readers in visualizing and understanding the physics of the buckyball artificial spin ice. I strongly believe the manuscript is of interest to physicists working on artificial spin ices as well as the larger frustrated magnetism community, and it will motivate future studies on similar models defined on finite systems beyond the planar geometry. I thus recommend the publication of the manuscript in Communication Physics, after the authors address several minor issues listed below.

We thank the Reviewer for their positive recommendation of our work, and for their compliments regarding our efforts at visualization. We hope they will also approve of our inclusion of Supplementary Movies showing the full 3D magnetic configuration of the states in Fig. 2.

1. When I read the main text, I was expecting to see the Hamiltonian somewhere in the first few sections. It only appears as Eq. (1) under 'Methods', which is after 'Conclusion' in Sec. 6. For this paper, I think there is no problem for not showing the Hamiltonian in the main text, but perhaps the authors can explicitly refer to it e.g. in Sec. 2? A good place to do this is line 109: "The Hamiltonian and further details concerning the Monte Carlo simulations are given in the Methods section and in the Supplementary Information." This will be useful for non-experts who are not familiar with artificial spin ice.

We agree with the Reviewer that the dipolar Hamiltonian is better placed in the main text itself, since it will make the manuscript more accessible to those unfamiliar with artificial spin ice. Consequently, we have moved the following paragraph from the Methods and inserted it at the position suggested by the Reviewer:

> "This Hamiltonian is
>
> $$\mathcal{H} = D\sum_{i\neq j}^{N}\left[\frac{\hat{s}_i\cdot\hat{s}_j}{r_{ij}^3} - \frac{3(\hat{s}_i\cdot r_{ij})(\hat{s}_j\cdot r_{ij})}{r_{ij}^5}\right]$$
>
> such that spin i, located at position r_i, has a normalised moment $\hat{s}_i$. The vector $r_{ij} \equiv r_j - r_i$ points from spin i to spin j. The energy scale of the interaction is set

through the dipolar constant, $D = \mu_0(M_S V)^2/(4\pi a^3)$, where M_S and V are the saturation magnetisation and volume of each nanomagnet, respectively. The symbol μ_0 denotes the magnetic permeability of free space. The parameter V, which is the lattice constant, controls the scale of the distances between spins. Here, we set *a* = 1 since the spin and vertex positions have been calculated for a buckyball with unit edge length." [Line 117-124]

2. Throughout the paper, the heat capacity per spin is expressed in units of the Boltzmann constant k_B, while the entropy per spin is dimensionless. The authors state in Sec. S1 of the Supplementary Information that "Temperatures are thus quoted in units of k_B*T/J_{1nn}. In a similar way, the units of specific heat capacity and specific entropy are then 1/(k_B spin) and 1/spin, respectively." I don't quite understand why the units of entropy is different from heat capacity. For example, according to the integral in Eq. (S2), one should have [s]=[c_V]+[T]-[T]=[c_V] by dimensional analysis, regardless of what units are used for the temperature. Another way to see this is to consider the configurational entropy s=k_B*log(\Omega), where \Omega is the multiplicity and log(\Omega) is dimensionless. Can the authors clarify this?

The Reviewer is correct that we had mixed up our units and we are very grateful for this comment. As the Reviewer notes, from $S = (k_B/N)\log(\Omega)$, the units of specific entropy must be $(k_B \text{ spin}^{-1})$.

During our Monte Carlo simulations, the energy is quoted in units of D, the dipolar constant. The reciprocal temperature $\beta = 1/(k_B T)$ is quoted in units of D^{-1}. This ensures that the argument of the Boltzmann factor $\exp(-\beta E_{\text{Cost}})$ is dimensionless when attempting a Monte Carlo move with energy cost E_{Cost}. From the definition of the specific heat as given in the main text,

$$c_V = \frac{1}{N}\frac{\langle (E - \langle E \rangle)^2 \rangle}{k_B T^2}, \quad \text{(R1)}$$

it follows that the units of c_V are $(k_B \text{ spin}^{-1})$. These are the same as the units of specific entropy, as discussed above. The following changes have been made to correct this:

- All figure labels have been changed to ensure that the units of specific heat capacity and specific entropy are $(k_B \text{ spin}^{-1})$.
- The relevant sentence in the Supplementary Information, Section I, now reads, "The units of specific heat capacity and specific entropy are both $(k_B \text{ spin}^{-1})$."

Finally, we also note that we have improved the precision of our wording regarding the definition of the units of temperature. The temperatures used during the Monte Carlo simulations are thermal energy values $k_B T$, which have units of D. The quantity $k_B T$ is thus drawn from the range [10^{-5}, 10^4], and not $k_B T/J_{1\text{nn}}$, as previously written.

Before we had:

“For all of the simulations presented in the main text, and the majority of those in the Supplementary Information, we use a common set of temperatures, which sample the range $k_B T / J_{1\mathrm{nn}} \in [10^{-5}, 10^{4}]$.”

Now, we have:

“For all of the simulations presented in the main text, and the majority of those in the Supplementary Information, we use a common set of temperatures, which sample the range $k_B T \in [10^{-5}, 10^{4}]$. ... As we emphasise above, before plotting any quantity as a function of temperature, we normalise the temperatures, $k_B T$, by $J_{1\mathrm{nn}}$ to obtain a dimensionless temperature scale.”

3. In Sec. 5, the authors associate the residual entropy obtained numerically with the total number of ground state configurations resulting from symmetry permutations of the pair of topological defects. The author writes "...these topological defects can form in any six different locations around pairs of diametrically-opposite pentagons. By symmetry, it is possible to show that there are 480 permutations of such defect pairs..." There are in total six pairs of diametrically-opposite pentagons in the buckyball, and clearly 6*6=36 is far less than the 480 permutations stated by the authors. I would appreciate if the authors can provide a more detailed explanation for the counting e.g. in the Supplemental Information.

We are grateful to the Reviewer for noticing this. Our original formulation—“six different locations around pairs of diametrically-opposite pentagons”—did not convey the correct meaning. We intended the following: the pair of topological defects can form in any of six locations in the buckyball artificial spin ice. These six locations each feature two diametrically-opposite pentagons. Each topological defect pole forms around one of the pentagons. We have updated the wording in this section to,

“Each of the pair of topological defects forms around a flux-closed pentagonal face. The locations of the topological defects are not independent from each other. In fact, they are arranged on diametrically-opposite pentagons. There are six possible ways to choose two diametrically-opposite pentagons in the buckyball. ” [Line 303 – 306]

Before addressing the rest of the Reviewer's question regarding the counting, we note a change in how we depict spin motifs in the ground state. Specifically, for faces that exhibit a net moment (shown in grey), we now include a flat arrow at the centre of each face to indicate the direction of the net moment. This update has been applied to the ground state motifs in Fig. 4**c**, which now appears as:

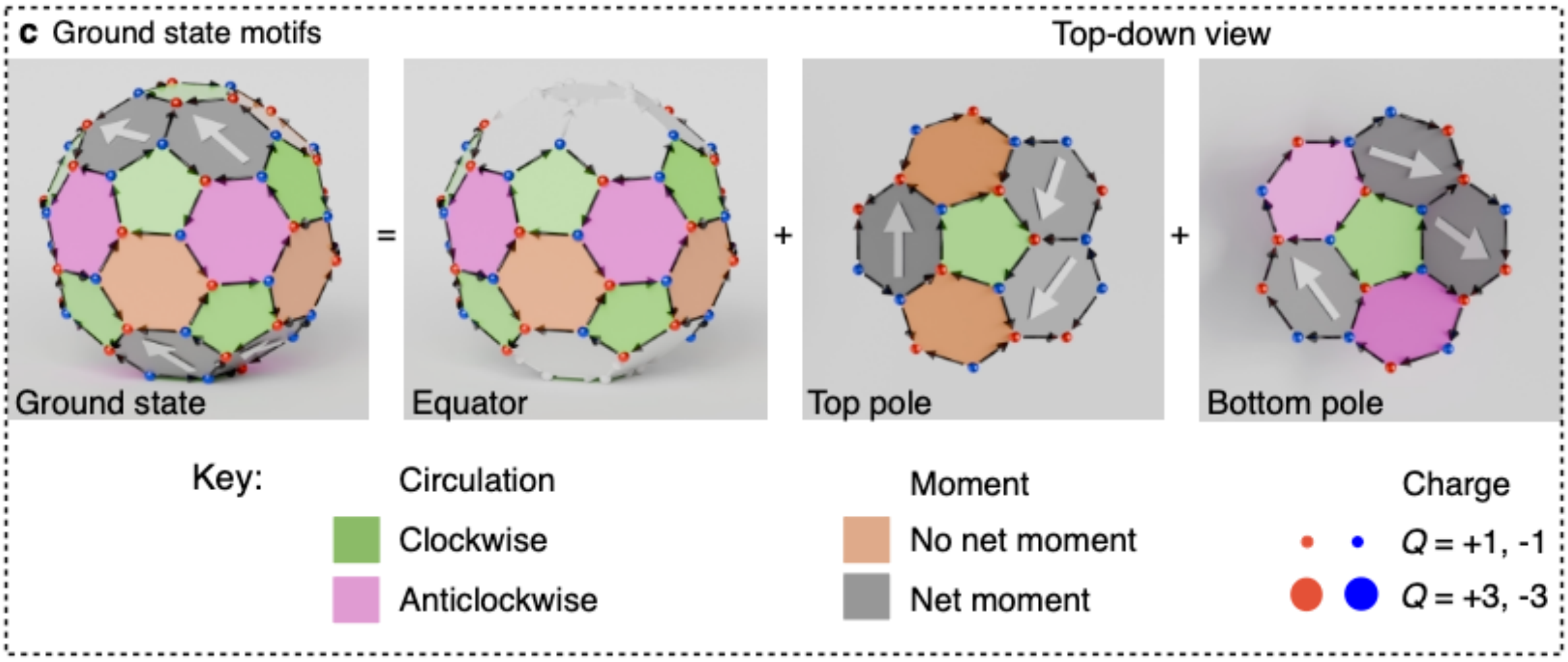

Figure 4 | Three-stage spin ordering in the buckyball artificial spin ice. c, For those hexagons with a net moment, coloured in grey, a flat arrow is additionally included to indicate the direction of the moment.

Furthermore, we have added a new section to the Supplementary Information, S6 Symmetries and Degeneracy of the Buckyball Artificial Spin Ice Ground State, which explains how the figure of 480 ground states is obtained. This section includes two new figures:

- Fig. S14 | Symmetries of the buckyball ground state; and
- Fig. S15 | Degenerate ground state configurations.

To answer the question of the Reviewer, we reproduce these figures together with the accompanying text from Section S7.

"S7 Symmetries and Degeneracy of the Buckyball Artificial Spin Ice Ground State

In the main text, we remarked that the buckyball artificial spin ice has 480 ground states, resulting in a residual entropy of $S_0 \approx 0.0695\ k_B$ per spin. In this section, we discuss how these ground states are related to each other. To help our discussion, we note some features of the ground state motifs depicted in main text, Fig. 4:

- All 12 pentagonal faces are flux-closed in the same sense (in this example, clockwise) and are coloured green according to the key.
- Of the 20 hexagonal faces, seven are flux-closed, all in the opposite sense, i.e. anticlockwise. Five of these anticlockwise hexagons are located around the equator, while the remaining two occur at the bottom pole. These faces are coloured purple.
- Considering just the equatorial motif, the five flux-closed hexagons occupy the upper row of hexagons along the equator. The five hexagons in which the constituent spins have neither net moment nor circulation are arranged along the lower row; these faces are coloured orange.
- Considering the poles, the top pole consists of hexagons with no net moment, situated on the same side of the equator that hosts the anticlockwise hexagons.

Conversely, the bottom pole contains the two anticlockwise flux-closed hexagons, sitting on the same side of the equator that hosts the two hexagons with no net moment.

As we shall see, the last point is generally true for all ground states of the buckyball artificial spin ice: when a pole contains flux-closed hexagons, it lies on the same side of the equator that contains hexagons with no net moment; and, vice versa, when a pole contains hexagons with no net moment, it lies on the side of the equator that contains flux-closed hexagons.

The 480 distinct ground states are related by a combination of global symmetries of the buckyball and local spin rearrangements that map one ground state onto another without changing the energy of the system.

Symmetries of the Ground State

There are four separate symmetries of the ground state of the buckyball artificial spin ice, which we show in Fig. S14. Here, a symmetry is an operation in which every spin and vertex charge in the buckyball artificial spin ice can be mapped to a new spin and vertex charge to produce a new ground state.

1. Orientational Symmetry (Fig. S14**a**)
   There are six distinct orientations of the buckyball, each defined by the axis that passes through the centres of a pair of diametrically-opposite pentagons (**×6**). This is a symmetry of the underlying buckyball solid, which is respected also by the magnetic ground states of the buckyball artificial spin ice.

2. Rotational Symmetry (Fig. S14**b**)
   Any magnetic ground state exhibits five-fold rotational symmetry about this axis (**×5**).

3. Global Ising Symmetry (Fig. S14**c**):
   The system also exhibits an overall Ising symmetry: globally reversing all spins in the buckyball inverts the sense of circulation around all flux-closed faces and the net moment of all polarized hexagons (**×2**).

4. Polar-Exchange Symmetry (Fig. S14**d**)
   The top and bottom poles can be interchanged, provided that the flux-closed hexagons and the hexagons with no net moment in the equator are also exchanged (**×2**). While we refer to this as a *polar-exchange symmetry*, it is important to note that the buckyball is not symmetric about its *z*-axis, and this operation does not simply correspond to swapping the top and bottom halves. Rather, to connect this to a genuine geometric symmetry of the underlying buckyball solid, it should be viewed as a half-turn rotation about an axis passing through the midpoints of two spins located opposite each other along the equator, as shown in the sub-panel of Fig. S15**d**, labelled “Example axis”.

Together, these symmetries contribute a factor of 6 × 5 × 2 × 2 = 120 to the ground state degeneracy.

Degenerate Spin Configurations

For a given combination of the symmetries described above, there exist four equivalent ground-state configurations, shown in Fig. S15. The left-most panel, Fig. S15**a**, corresponds to the configuration presented in the Figs. 2 and 4 of the main text. In the remaining three panels, the spins that are reversed relative to Fig. S15**a** are highlighted in yellow. Below, we briefly describe how each configuration differs from that in Fig. S15**a**.

Fig. S15**b**:	A new ground state is obtained by flipping four spins located in the bottom pole, while keeping both the top pole and the equator unchanged. This transformation is equivalent to rotating the bottom pole by 72° counterclockwise, leaving the remainder of the structure unaltered.
Fig. S15**c**:	This configuration is produced by flipping two specific spins, one in each pole. In the top pole, the arrangement of the hexagons with net moment remains the same; however, the direction of the net moment on one face is reversed and now points away from a vertex of the central pentagon, whereas in Fig. S15**a** it pointed towards that vertex of the central pentagon. In the bottom pole, the positions of the hexagons with net moment are shifted relative to Fig. S15**a**, and one of these faces now has its moment directed inward toward the central pentagon, rather than outward.
Fig. S15**d**:	This ground state is obtained by flipping six spins: one in the top pole (the same spin flipped in Fig. S15**c**) and five in the lower pole. In the lower pole, these are the five spins whose edges adjoin the central pentagonal face but are not themselves part of it. The flipped spin in the top pole produces the same change observed as in Fig. S15**c**. Reversing the five spins in the bottom pole modifies the arrangement of the hexagons carrying net moment, such that one of them now points inward toward a vertex of the central pentagon where it previously pointed out from that vertex of the central pentagon.

From these configurations, a clear relationship emerges:

- The configurations in Figs. S15**a** and S15**b** share the same top-pole and equator motifs, with the bottom pole in S15**b** rotated 72° anticlockwise relative to S15**a**.
- Similarly, the configurations in Figs. S15**c** and S15**d** share the same top-pole and equator motifs, with the bottom pole in Fig. S15**d** rotated 72° anticlockwise relative to S15**c**.

Multiplying these contributions yields a total ground state degeneracy of:

$$4 \text{ degenerate states} \times (120 \text{ symmetries}) = 480 \text{ configurations.} \qquad \text{(S3)}$$

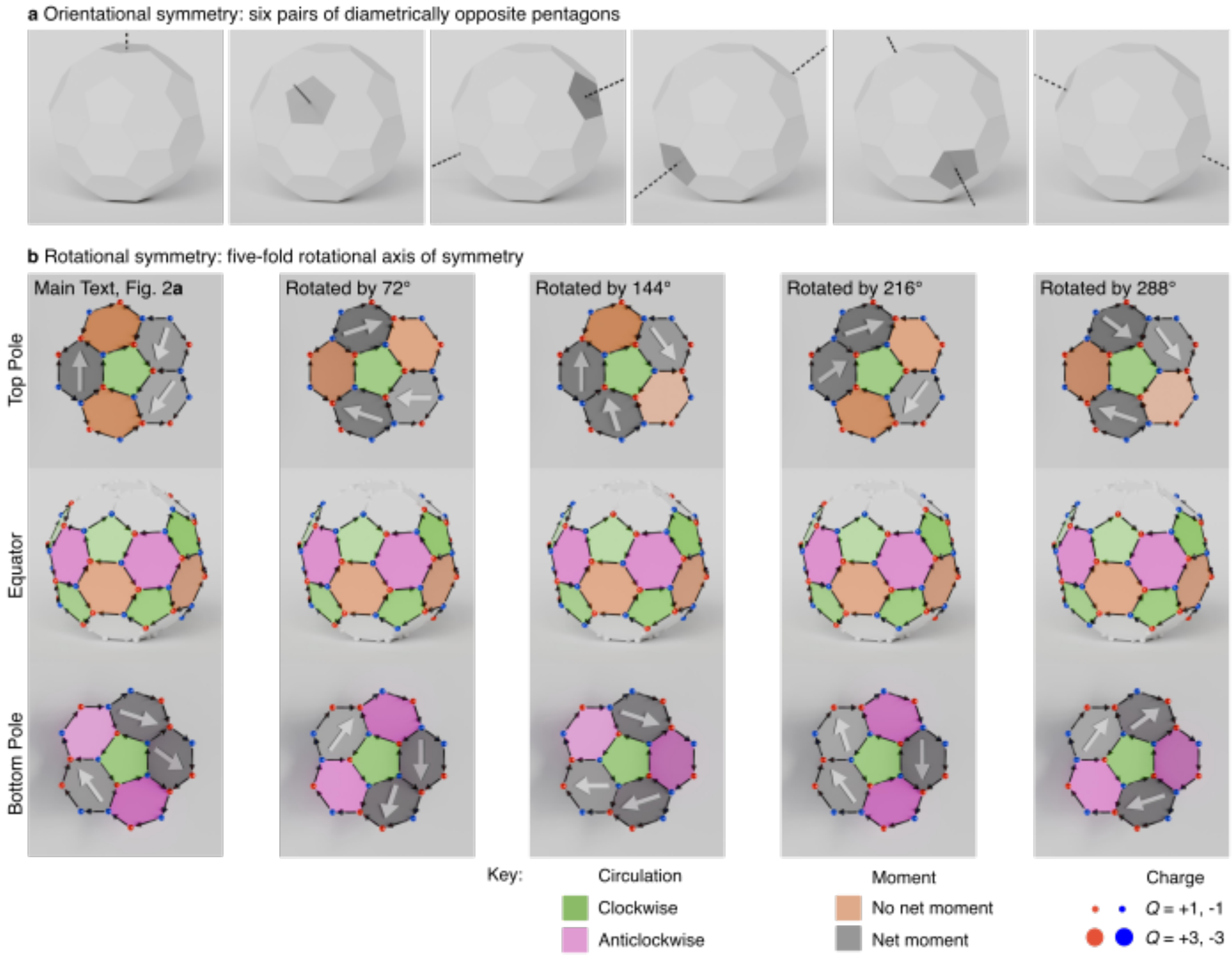

Figure S14. Symmetries of the buckyball artificial spin ice ground state. a, The buckyball contains six pairs of diametrically-opposite pentagons. In the corresponding artificial spin ice lattice, pairs of topological defect poles can form at any of these six locations. A line connecting each pair of diametrically-opposite pentagons defines a symmetry axis. Each pair of diametrically opposite pentagons is coloured in dark grey in a separate sub-panel. **b**, Around each such axis, the magnetic ground state of the buckyball artificial spin ice exhibits five-fold rotational symmetry. The leftmost panel is the ground state configuration shown in main text, Fig. 2**a**. We use this configuration throughout this figure to illustrate how new ground states can be obtained by performing certain operations on this one. Additional degenerate configurations can be generated by rotating the spin configuration by 72° about the axis joining the centres of the two diametrically-opposite pentagons. Faces are coloured according to the configuration of their associated spins, following the same colour scheme used in the main text. Figure continued on next page.

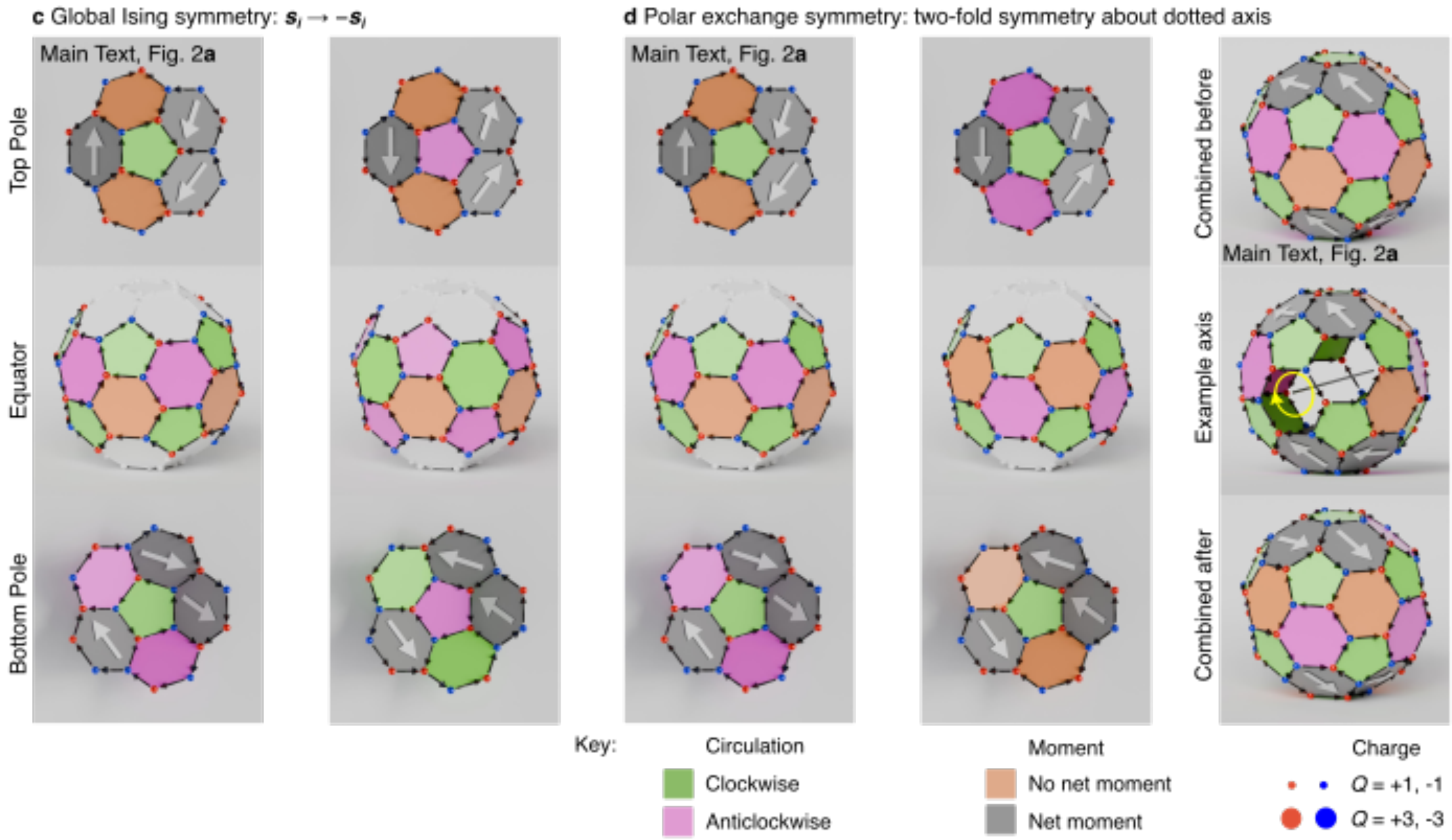

Fig. S14 (cont.). Symmetries of the buckyball artificial spin ice ground state. c, A global two-fold Ising symmetry, $\mathbf{s}_i \rightarrow -\mathbf{s}_i$, in which flipping all spins reverses the circulation of every flux-closed face and inverts the net moment of each face with a non-zero moment. **d**, Two-fold polar-exchange symmetry, in which the top and bottom poles are interchanged together with the positions of the flux-closed hexagons and the hexagons with no net moment in the equator. This symmetry is not a simple inversion about the *z*-axis. Rather, it corresponds to a half-turn rotation about an axis passing through the centres of two spins located opposite each other along the equator. The third column illustrates this construction: the top sub-panel shows the combined motifs of the north pole, equator, and south pole; the middle sub-panel presents the same configuration from a slightly different angle with the coloured faces removed and an example rotation axis indicated; and the bottom sub-panel shows the resulting configuration after performing the half-turn about this axis.

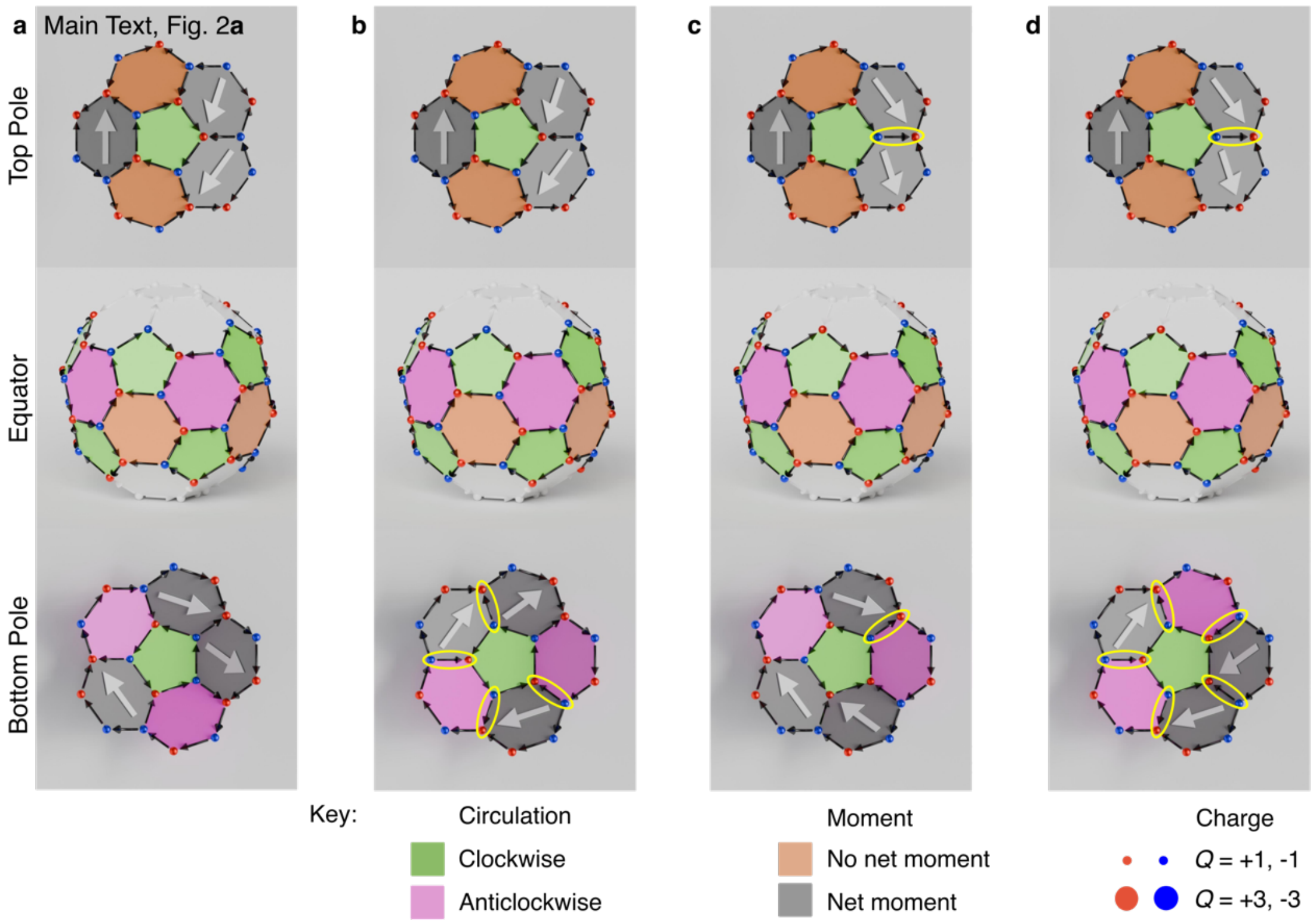

Figure S15 | Degenerate ground state configurations. **a**, Top-pole, equator, and bottom-pole motifs corresponding to the configuration shown in the main text, Fig. 2**a**. In the subsequent panels, spins that are flipped relative to panel **a** are highlighted with yellow circles. **b**, Equivalent ground state obtained by reversing four spins in the bottom pole, with the top-pole and equator motifs unchanged. **c**, Equivalent ground state obtained by reversing two specific spins, one in each pole. **d**, Equivalent ground state obtained by reversing the same top-pole spin as in panel **c**, together with the five spins in the bottom pole that abut the central pentagonal face. All remaining ground states can be generated from these four configurations by applying the symmetry operations described in Fig. S14.

We hope this additional information satisfies the Reviewer's interest in how the ground states are constructed. We thank them for raising it, since we feel it has filled in a gap that was missing from our manuscript!

4. In line 181, the authors write "...are are not associated with..." Please remove the duplicate "are".

Many thanks for spotting this typo. The relevant sentence now reads,

> "Similarly, the low-temperature crossovers are also not associated with any change in the loop structure on the buckyball or in the number of closed-loop faces (Supplementary Information, Sections I and V)." [199-200]

5. The authors should use a uniform notation k_B for the Boltzmann constant. In Fig. 1c and line 366, please change k_b (lowercase b) to k_B (uppercase B).

Many thanks for spotting this. We have corrected them in both Fig. 1c and in the relevant line of text. Additionally, we have reviewed all other figures in the main text and Supplementary Information to ensure consistent notation. During this process, we noticed that the same typo appeared in Fig. S6, which we have also corrected.

6. In Eqs. (3) and (S2) showing how the entropy is calculated from the heat capacity, shouldn't the upper/lower limit of the integral be infinity/T? Starting from c_V=T*ds/dT, one gets ds=(c_V/T)*dT and then integrates both sides from T'=T to T'=infinity, while ln(2) (possibly multiplied by k_B, see point #2) corresponds to the entropy at infinite temperature.

The Reviewer is correct: the limits of the integration must be reversed, and it is necessary to include the factor of k_B to ensure that the specific entropy has the correct units. The expression for the specific entropy should read

$$s(T) = k_B \ln(2) - \int_T^{\infty} \frac{c_V}{T'} \mathrm{d}T' \, . \qquad \text{(R3)}$$

Both eq. (3) and eq. (S2) have been corrected. Many thanks for pointing this out.

7. In line 158 of the Supplementary Information, the authors write "...where loop moves are used (blue circles) and where only single spin flips are used (red crosses)." Referring to Fig. S7a, the symbols in the parentheses should be blue crosses and red circles, respectively.

Many thanks for pointing this out. This has been corrected so that the sentence fragment reads,

> “for the case where loop moves are used (blue crosses) and where only single spin flips are used (red circles).”

8. In the caption of Fig. S10, the authors write "...Type 2B vertices (orange curve)...". The typo "Type 2B" should be corrected to "Type 1B".

Many thanks for spotting this. This typo has been corrected so that the sentence fragment reads,

> “... Type 1B vertices (orange curve)...”.

We thank the Reviewer for their thorough reading of our manuscript.

Other minor changes:

1. Affiliations 2 and 3 have been updated. The present address and an updated email have been added for GMM.

2. "By symmetry, it is possible to show that there are 480 permutations of such defect~~s~~ pairs, which results in a residual entropy of $S_0 \approx 0.0685\,k_B$ per spin." [Line 306]
   The typo "defects pairs" has been corrected to "defect pairs".

3. "Low energy vertices are those that obey an ice-rule: $Q = \pm 1$ with either a 2-in-1-out or 2-out-1-in spin configuration."
   The sentence has been changed to,
   "Low energy vertices are those that obey an ice-rule: $Q = \pm 1$ with either a two-in-one-out or two-out-one-in spin configuration." [Line 103]

4. A misalignment of the dotted box in Figure 4 has been corrected.
