# Supplementary material for: Five-stage ordering to a topological-defect-mediated ground state in a buckyball artificial spin ice
Source: Commun Phys. 2026 May 26;9(1):288. doi: 10.1038/s42005-026-02663-y (PMC13549942; doi:10.1038/s42005-026-02663-y)
Supplement: Supplementary file 2 [file 42005_2026_2663_MOESM2_ESM.pdf]

Supplementary Information: Five-stage ordering to a topological-defect-mediated ground state in a buckyball artificial spin ice

Gavin M. Macauley[1,2,*,+], **Luca Berchialla**[1,2], **Peter M. Derlet**[2,3,*], **and Laura J. Heyderman**[1,2,*]

[1]Laboratory for Mesoscopic Systems, Department of Materials, ETH Zurich, 8093 Zurich, Switzerland
[2]PSI Center for Neutron and Muon Sciences, 5232 Villigen PSI, Switzerland
[3]PSI Center for Scientific Computing, Theory and Data, 5232 Villigen PSI, Switzerland
[*]Corresponding authors: gm3291@princeton.edu, peter.derlet@psi.ch, laura.heyderman@psi.ch
[+]Present address: Department of Physics, Princeton University, Princeton, NJ 08540 USA

Supplementary Note 1 Further details about the Monte Carlo Simulations

The buckyball artificial spin ice is approximated by placing a point dipole at the midpoint of each edge of the solid. Similar to two-dimensional (2D) artificial spin ices, we assume that each point dipole is constrained to point along one of two directions, parallel to its associated edge. Each point dipole then represents the moment in an isolated nanomagnet. To simulate the behaviour of the buckyball artificial spin ice, we perform extensive Monte Carlo simulations using the Metropolis-Hastings Algorithm with (i) single spin flips and (ii) loop moves. As described in the main text, the Hamiltonian of our buckyball artificial spin ice is

$$\mathscr{H} = D\sum_{i\neq j}^{N}\left[\frac{\hat{\mathbf{s}}_i\cdot\hat{\mathbf{s}}_j}{r_{ij}^3} - \frac{3(\hat{\mathbf{s}}_i\cdot\mathbf{r}_{ij})(\hat{\mathbf{s}}_j\cdot\mathbf{r}_{ij})}{r_{ij}^5}\right], \tag{S1}$$

such that spin i, located at position $\mathbf{r}_i$, has a normalised moment $\hat{\mathbf{s}}_i$. The displacement vector $\mathbf{r}_{ij} \equiv \mathbf{r}_j - \mathbf{r}_i$ points from spin i to spin j. The energy scale of the interaction is set through the dipolar constant, $D = \mu_0(M_S V)^2/(4\pi a^3)$, where M_S and V are the saturation magnetisation and volume of each nanomagnet, respectively, and μ_0 is the vacuum permeability. We assume a regular buckyball, which has unit edge length and so the lattice constant $a = 1$.

For the results presented in the main text, the full-dipolar sum in Eq. (S1) is implemented. In Supplementary Note 2, we discuss the effect of applying a cutoff to this Hamiltonian, beyond which interactions are neglected.

As noted in the Methods section of the main text, it is convenient to work in reduced units and to rescale temperatures to the largest energy in the system, which is the first-nearest-neighbour interaction strength $J_{1\text{nn}} \approx 3.1424\,D$. Temperatures are thus quoted in units of $k_BT/J_{1\text{nn}}$. The units of specific heat capacity and specific entropy are both (k_B spin^{-1}).

For all of the simulations presented in the main text, and the majority of those in the Supplementary Information, we use a common set of temperatures, which sample the range $k_BT \in \left[10^{-5}, 10^4\right]$. At high temperatures (in the range 10^4 to 10^1), these points are distributed linearly, while at low temperatures (in the range 10^1 to 10^{-5}), they are logarithmically spaced. The change between the two sampling regimes occurs at $k_BT = 1$. This specific temperature set was chosen to ensure that all peaks in observables (e.g. heat capacity, correlation functions and susceptibilities) can be well discriminated. The starting state

at a particular temperature is the final configuration from the previous temperature, i.e. the whole process is a simulated anneal with 909 temperature points. As we emphasise above, before plotting any quantity as a function of temperature, we normalise the temperatures, k_BT, by $J_{1\mathrm{nn}}$ to obtain a dimensionless temperature scale.

The definition of the specific heat capacity $c_\mathrm{V}(T)$ was given in the main text. The specific entropy $s(T)$ is obtained through numerical integration of c_V as follows

$$s(T) = k_B \ln(2) - \int_T^\infty \frac{c_\mathrm{V}}{T'} \mathrm{d}T'. \tag{S2}$$

Since each spin has an Ising degree of freedom, the entropy per spin in the high temperature limit is $k_B \ln(2)$. The residual entropy S_0, which is a measure of the degeneracy of the ground state, is given by evaluating Eq. (S2) for $T \to 0$.

The buckyball artificial spin ice has 90 edges so that, with a spin placed on the midpoint of every edge, the number of spins in the lattice is $N_\mathrm{spins} = 90$. For the results presented in the main text, a single Monte Carlo step (MCS) involves N_spins attempted single spin flips, followed by N_spins attempted loop moves. At each temperature, the buckyball artificial spin ice is equilibrated for 10^4 Monte Carlo steps (referred to as the 'burn-in' period), before 10^4 Monte Carlo steps are used for the averaging of relevant thermodynamic quantities.

The remainder of this section is structured as follows:

- In Supplementary Note 1.1, we verify that 10^4 Monte Carlo steps are sufficient to ensure the buckyball artificial spin ice is well-equilibrated, and that the averages we show in the main text have sufficiently small statistical errors.

- In Supplementary Note 1.2, we present results on the importance of loop moves for obtaining the ground state of the buckyball artificial spin ice. We examine how the permitted length of a loop move influences the ability of the buckyball artificial spin ice to reach its ground state following a simulated anneal. Interestingly, our findings reveal that accurately recovering the thermal properties necessitates including loop moves involving spins on pentagonal faces. We comment on the spin configuration obtained when only single spin flips are used.

- In Supplementary Note 1.3, we discuss the number of loop moves that must be performed at each Monte Carlo step to obtain the equilibrium thermal properties.

Supplementary Note 1.1 Equilibration

In general, it is difficult to ascertain the appropriate number of Monte Carlo steps N_MCS to ensure that a spin system is well-equilibrated and, consequently, that the averages of the observables reflect the Boltzmann distribution at that temperature. For the graphs presented in the main text, we have used 10^4 Monte Carlo steps. To provide evidence that this is a sufficient number of Monte Carlo steps, we repeat our calculation of the specific heat capacity for a number of different burn-in and averaging Monte Carlo steps. This is displayed in Fig. S1**a**. Beyond 10^3 Monte Carlo steps, the curves for each heat capacity overlap, and no shift is seen in either the position or height of the peaks—at least within the resolution given by the set of temperatures. In Fig. S1**b**, the residual entropy is displayed as a function of N_MCS. Similarly, above 10^3 Monte Carlo steps, the residual entropy S_0 does not significantly change with the values for 10^3 Monte Carlo steps and 10^4 Monte Carlo steps being the same to within error bounds: $0.071(3)k_B$ per spin and $0.069(3)k_B$ per spin, respectively. The inset in Fig. S1**b** shows the same data but plotted as a function of the inverse number of Monte Carlo steps. Fitting a straight line to this data and

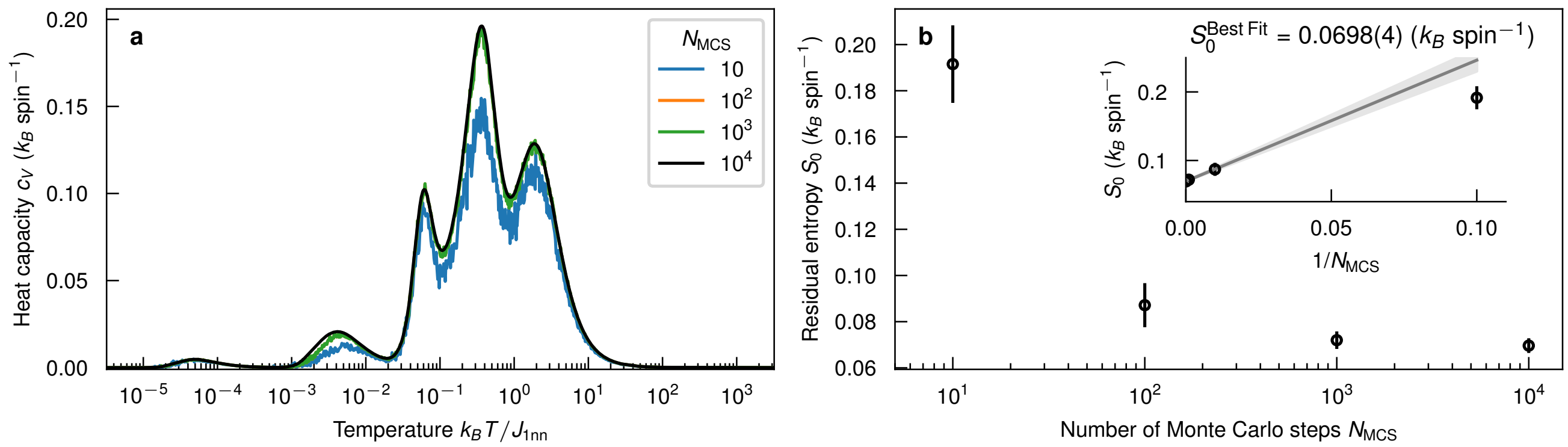

Figure S1. Effect of increasing the number of Monte Carlo Steps. a, The specific heat capacity is recorded for different numbers of Monte Carlo steps N_{MCS}. The legend refers to the number of Monte Carlo steps used during both the burn-in and averaging portions of the simulated anneal. A common set of temperature points is used for all of the simulations. The black reference line, i.e. for 10^4 Monte Carlo steps, is the heat capacity curve that appears in Figure 1**c** of the main text. For the case with 10^4 Monte Carlo steps, 5000 independent runs are averaged; all the other curves reflect the average of 1000 independent runs. To better discriminate the curves, the standard deviation is not shown. **b**, Corresponding residual entropies S_0 for the curves in panel **a**, plotted as a function of the number of Monte Carlo steps performed at each temperature point in each simulation. The residual entropy is obtained by numerical integration of Eq. (S2) for each curve in panel **a**. Error bars reflect $\pm\, 1\sigma$ about the mean. The value of the residual entropy when 10^3 Monte Carlo steps are used, $S_0 = 0.071(3)k_B$ per spin, is within the error bounds of that obtained when 10^4 Monte Carlo steps are used, $S_0 = 0.069(3)k_B$ per spin. Inset: same data, but as a function of $1/N_{\mathrm{MCS}}$. Using a linear fit and extrapolating to find the y-intercept gives the best fit S_0 of $0.0698(4)k_B$ per spin.

extrapolating to find its y-intercept, we obtain a value for the residual entropy in the limit $N_{\mathrm{MCS}} \to \infty$, namely $S_0^{\mathrm{Best\,Fit}} = 0.0698(4)k_B$ per spin. Here, as in the main text, the number in brackets indicates the $\pm 1\sigma$ uncertainty in the last digit, where σ is the standard deviation.

Supplementary Note 1.2 Effect of including loop moves

As discussed in the main text, a loop is any closed chain of head-to-tail spins in the lattice. Flipping all of the spins in such a loop preserves the charge at every vertex through which the loop passes. Such loop moves are useful when simulating frustrated systems since they allow access to the low-temperature sectors of the phase diagram, where either the waiting time to access the new state is prohibitively long, or single spin flips are frozen out completely. For the buckyball artificial spin ice, these loops may involve the spins around a single hexagonal or pentagonal face, or the loops may occur across a number of faces. We quantify some of the properties of such loops in Supplementary Note 5. For the data displayed in all figures in the main text, loop moves are used during both the burn-in and averaging sections of the simulated anneal.

In Fig. S2, we present the effect of including loop moves on the heat capacity. Fig. S2**a** displays c_{V} as a function of temperature for the case where loop moves are used in both the burn-in and averaging steps as in the main text (blue curve), and where only single spin flips are used (red curve). When only single spin flips are used in the Monte Carlo anneal, only four peaks are present in the heat capacity, rather than the five peaks seen when loop moves are incorporated. For the case of single spin flips only, a broad peak occurs

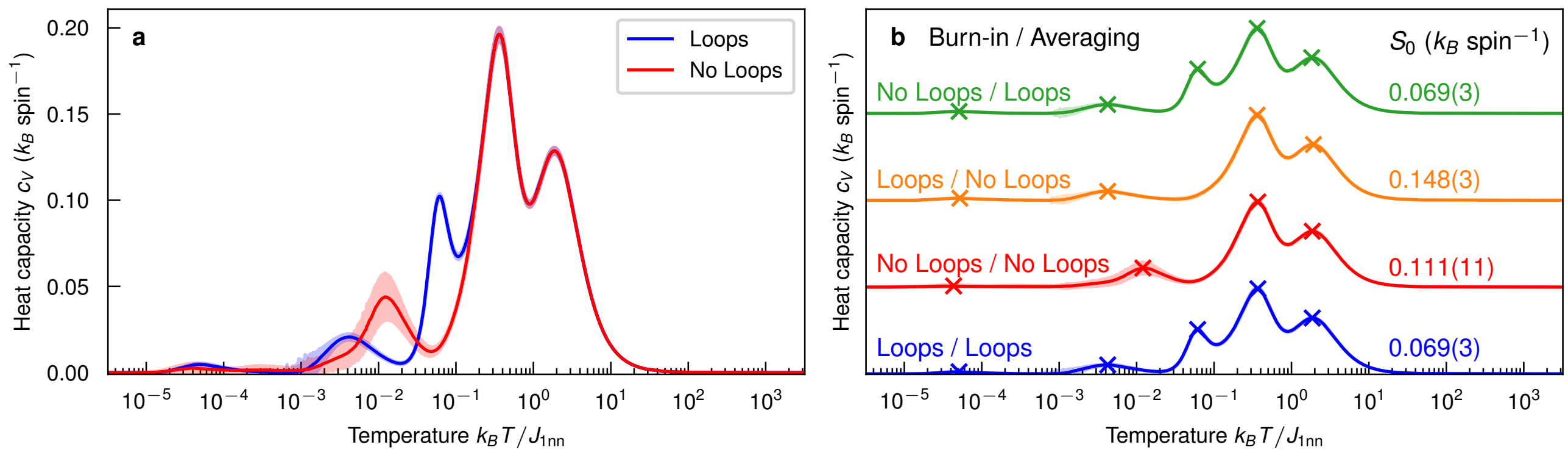

Figure S2. Effect of including loop moves on the heat capacity of the buckyball artificial spin ice. **a**, Heat capacity as a function of temperature obtained when loop moves are used during both burn-in and averaging as in the main text (blue curve), and the heat capacity obtained when only single spin flips are used (red curve). When only single spin flips are used, only four peaks appear in the heat capacity, in contrast to the five that are present when loop moves are used. **b**, Waterfall plot of the specific heat capacity as a function of temperature, where loop moves are introduced separately during both burn-in and averaging. Peaks are indicated in each case by crosses. Lines are labelled explicitly according to whether loop moves are used during 'Burn-in' or 'Averaging'. The residual entropy for each curve is indicated to the right. Only when loop moves are used during the averaging portion of the anneal are all five peaks recovered. For the case of 'Loops/Loops' (blue line, as in the main text), 5000 independent runs are averaged; all other curves reflect the average of 2000 independent runs.

at $k_B T/J_{1\mathrm{nn}} \approx 10^{-2}$, which is associated with the onset of limited spin order in the buckyball artificial spin ice. Concomitantly, the residual entropy is also higher (not shown), $S_0^{\mathrm{No\,Loops}} = 0.111(11)k_B$ per spin, which is consistent with the notion that the lowest-energy states remain inaccessible.

In Fig. S2**b**, we show the remaining cases where loop moves are used *only* during the burn-in or *only* during the averaging sections of the simulated annealing (orange and green curves). The five peaks shown in the main text occur only when loop moves are used during thermal averaging. We conclude that the three low-temperature peaks can only be efficiently sampled through loop moves. We answer exactly how many loop moves are required to obtain the full set of five peaks in Supplementary Note 1.3.

In the main text, we impose no restriction on the loop length L, as long as the spins in the loop form a head-to-tail (flux-closed) configuration. In Fig. S3, we show the impact on the specific heat capacity when loop moves are restricted to a fixed length L, such as loops formed by spins around a single pentagonal face ($L = 5$) or hexagonal face ($L = 6$), together with the cases where either general loop moves are allowed or only spin flips are performed.

We find that the characteristic five peaks in the specific heat of the buckyball artificial spin ice are only recovered when loop moves involving spins on pentagonal faces are allowed. This may seem surprising: we refer to the low-temperature sectors as Hexagon I and Hexagon II because their structure is governed by the freezing of those spins lining hexagonal faces and, yet, it appears that the spins lining pentagonal faces are more important in accessing this ground state. Nonetheless, we can provide a plausible justification for the importance of the pentagon loop moves. In the Hexagon I and Hexagon II sectors, 13 of the 20 hexagons in the buckyball artificial spin ice either carry a net moment, or have neither a net moment nor a net circulation. This means that their spins do not form flux-closed configurations, and are therefore not valid candidates for loop moves. As a result, if only hexagonal loop moves are attempted,

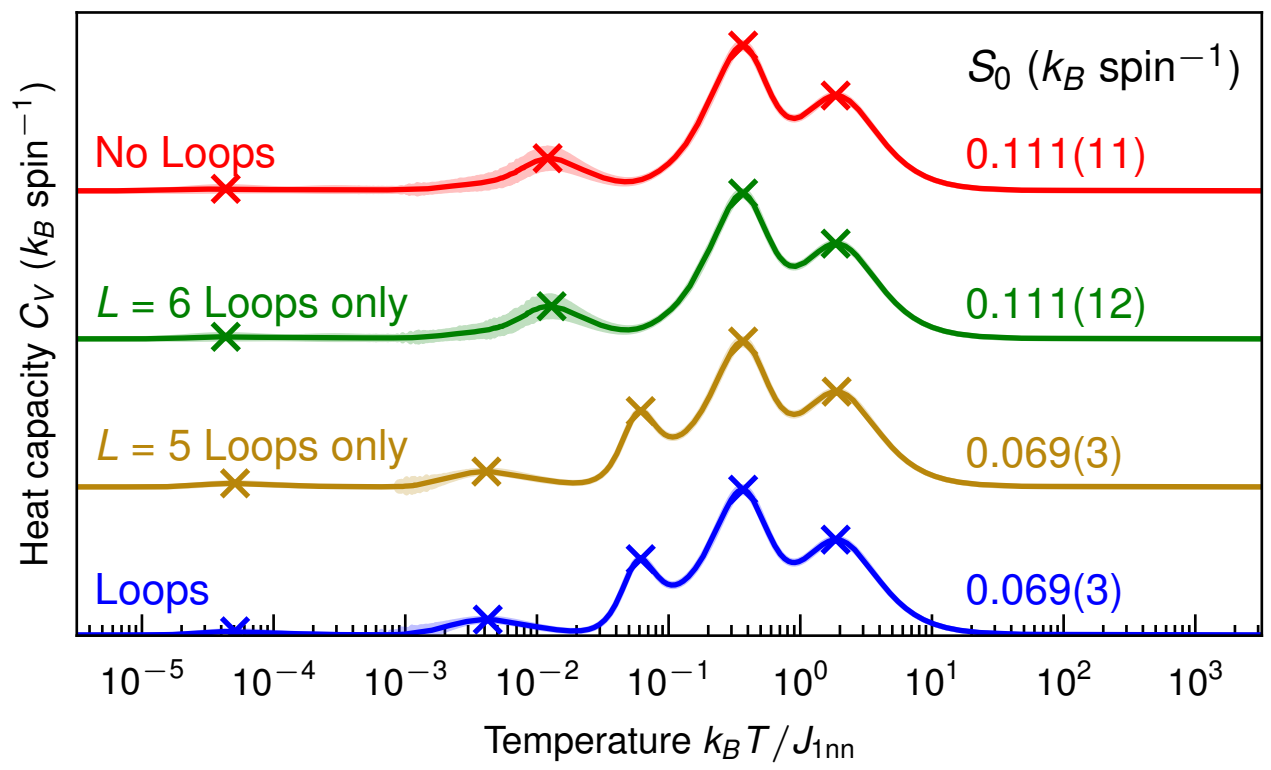

Figure S3. Effect of allowing only loop moves of a certain length on the specific heat capacity of the buckyball artificial spin ice. The specific heat capacity as a function of temperature is shown for four cases: when loop moves of arbitrary length are allowed (blue); when only loop moves of length $L = 5$ are allowed, i.e., involving spins on pentagonal faces (orange); when only loop moves of length $L = 6$ are allowed, i.e., involving spins on hexagonal faces (green); and when no loop moves are attempted (red). The residual entropy for each curve is indicated to the right. We find that it is necessary to include loop moves of length $L = 5$ to reproduce the characteristic five peaks seen in the unrestricted (blue) loop case. In addition, the residual entropy for the $L = 5$ simulations is the same as that for the unrestricted case. This indicates that enabling collective flips of spins around flux-closed pentagonal loops is essential for recovering the correct thermal properties of the system.

the spin configurations of these hexagons cannot be rearranged. It is only by flipping the spins on the central flux-closed pentagon of each topological defect that all five surrounding hexagons be influenced simultaneously. This makes pentagonal loops essential for enabling the collective dynamics that govern the low-temperature thermal behavior of the system. As we show later in Supplementary Note 7, the ground states of the buckyball artificial spin ice obey specific rules governing the arrangement of hexagons with a net moment and those with neither a net moment nor net circulation.

We now briefly describe the main features of configurations obtained when only single spin flips are used. Since the two c_V curves in Fig. S2**a** agree down to $k_B T/J_{1\text{nn}} \approx 10^{-1}$, we can infer that the system still enters a Spin-Ice sector at $k_B T/J_{1\text{nn}} \approx 1.85$, followed by a crossover to an imperfect charge crystal at $k_B T/J_{1\text{nn}} \approx 0.360$. Below this, the two curves diverge. With only single spin flips, fewer hexagons flux close in the low-temperature regime compared with the case when loop moves are included (Fig. S4**a**). This means that no large, flux-closed motif forms. This is very different to the case when loop moves are included (Fig. 4, main text) where a belt of flux-closed pentagons and hexagons of opposite spin circulation forms, which encircles the buckyball. A typical $T = 0$ configuration obtained with only single spin flips is shown in Fig. S4**b**. This configuration is higher in energy than that obtained when loop moves are applied. This is because, while all the spins associated with the pentagonal faces are in a head-to-tail arrangement, they do not all have the same sense of circulation and, as a result, the ground state with the maximal number of flux-closed faces does not form.

We have therefore demonstrated that loop moves are essential for accessing the low-temperature crossovers in the buckyball artificial spin ice, with pentagonal loop moves being particularly important.

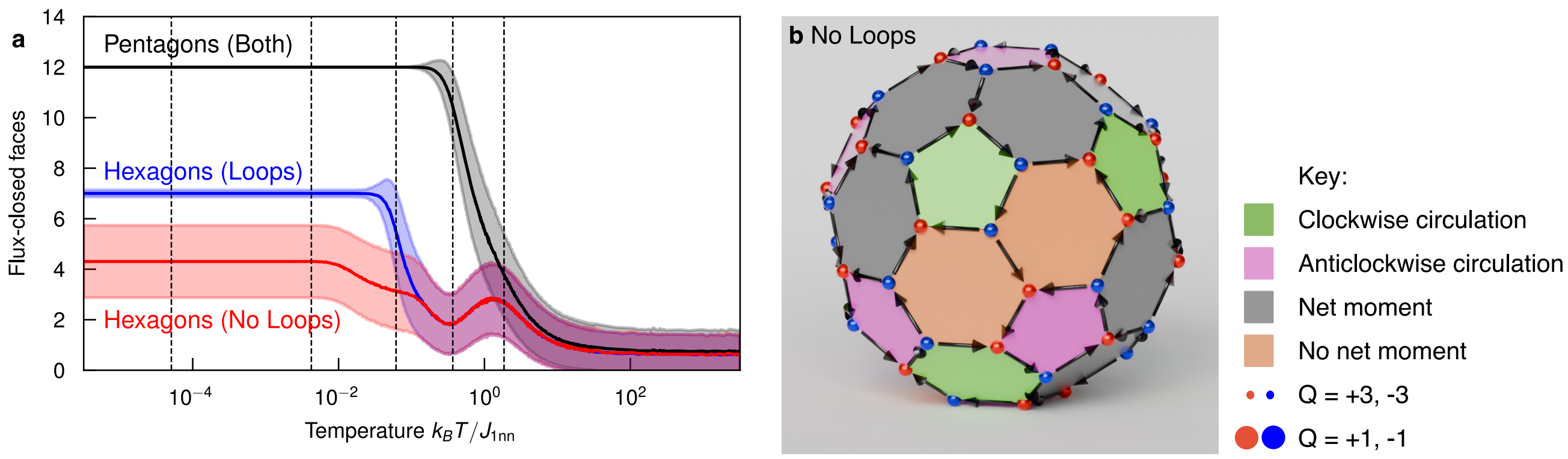

Figure S4. Simulated annealing of the buckyball artificial spin ice without loop moves. a, Number of flux-closed hexagonal faces when loop moves are used (blue) and when only single spin flips are used (red). Irrespective of whether loop moves or only single spins flips are used, the spins associated with all 12 pentagonal faces order into a head-to-tail arrangement. As such, only a single curve (black) is used to represent the behaviour of the pentagonal faces. The data for 'Loops' and 'No Loops' are averaged over 5000 and 2000 independent runs, respectively. **b**, Typical configuration obtained at $T = 0$ when only single spin flips are used. The direction of each spin and the charge at each vertex are shown. Faces are coloured according to the circulation of their associated spins as indicated by the key, using the same colour scheme as that used in Fig. 2 of the main text. While the spins associated with each pentagon point head-to-tail, system-spanning order is not established without loop moves.

Supplementary Note 1.3 Required number of attempted loop moves

For the results presented in the main text, each Monte Carlo step involved $N_{\mathrm{Spins}} = 90$ attempted loop moves. Much as with the discussion on the best number of Monte Carlo steps, it is not immediately apparent how many loop moves should be attempted during each sweep, although it is typical to choose a value on the same order as the number of proposed single spin flips, as in Refs. 1, 2.

We repeat our calculation of the heat capacity, varying the number of attempted loop moves during each Monte Carlo step. Since a Monte Carlo step plays a role analogous to time, the number of attempted loop moves per step is a measure of the frequency with which loop moves are attempted. In Fig. S5**a**, we show the change in residual entropy as a function of the average number of attempted loop moves per Monte Carlo step. A common temperature set is used for each simulation, and the same number of single spin flips is used during each Monte Carlo step. The data points are the average of 100 independent runs, except for the limiting case of 'No Loops' and 'Loops', which are averaged over 1000 and 5000 runs, respectively. The red and blue bands reflect the $\pm 1\sigma$ for the averages of the two limiting cases, $S_0^{\mathrm{No\,Loops}}$ and S_0^{Loops}, respectively. The uncertainty bands and the average residual entropies for these two cases are explicitly labelled in a colour scheme consistent with that of the curves in Fig. S2**a**. Rather than a monotonic decrease from the 'No Loops' value of $0.111(11)k_B$ per spin, S_0 first increases on increasing the number of attempted loop moves per Monte Carlo step, attaining a maximum when approximately 2×10^{-4} loops moves are performed per Monte Carlo step. The residual entropy then decreases beyond this point as more loop moves are attempted per Monte Carlo step, until it plateaus within error bounds of the limiting case $S_0^{\mathrm{Loops}} = 0.069k_B$ per spin when approximately 10^{-1} loop moves are performed per Monte Carlo step.

The non-trivial behaviour of S_0 can be explained by considering c_{V} calculated for different numbers of attempted loop moves per Monte Carlo step (Fig. S5**b**). In the limiting case when no loop moves are

performed (i.e. only single spin flips), there is a broad peak at $k_B T/J_{1\mathrm{nn}} \approx 10^{-2}$. This represents the onset of the limited spin order in the buckyball artificial spin ice where all pentagonal faces have flux-closed though not necessarily in the same sense (c.f. Fig. S4**b**). When only a few loop moves are performed (greater than zero but $\leq 10^{-4}$ per Monte Carlo step), this broad peak shifts to lower temperatures, leading to an overall increase in residual entropy. When more than 10^{-3} loop moves are attempted per Monte Carlo step, the peak associated with the crossover to the Pentagon sector begins to form. Increasing the average number of attempted loop moves per Monte Carlo step above 0.05 produces little change in the curves of specific heat capacity. Given this behaviour, we confirm that the choice used in the main text—90 attempted loop moves per Monte Carlo step—is more than sufficient to recover the equilibrium thermal properties.

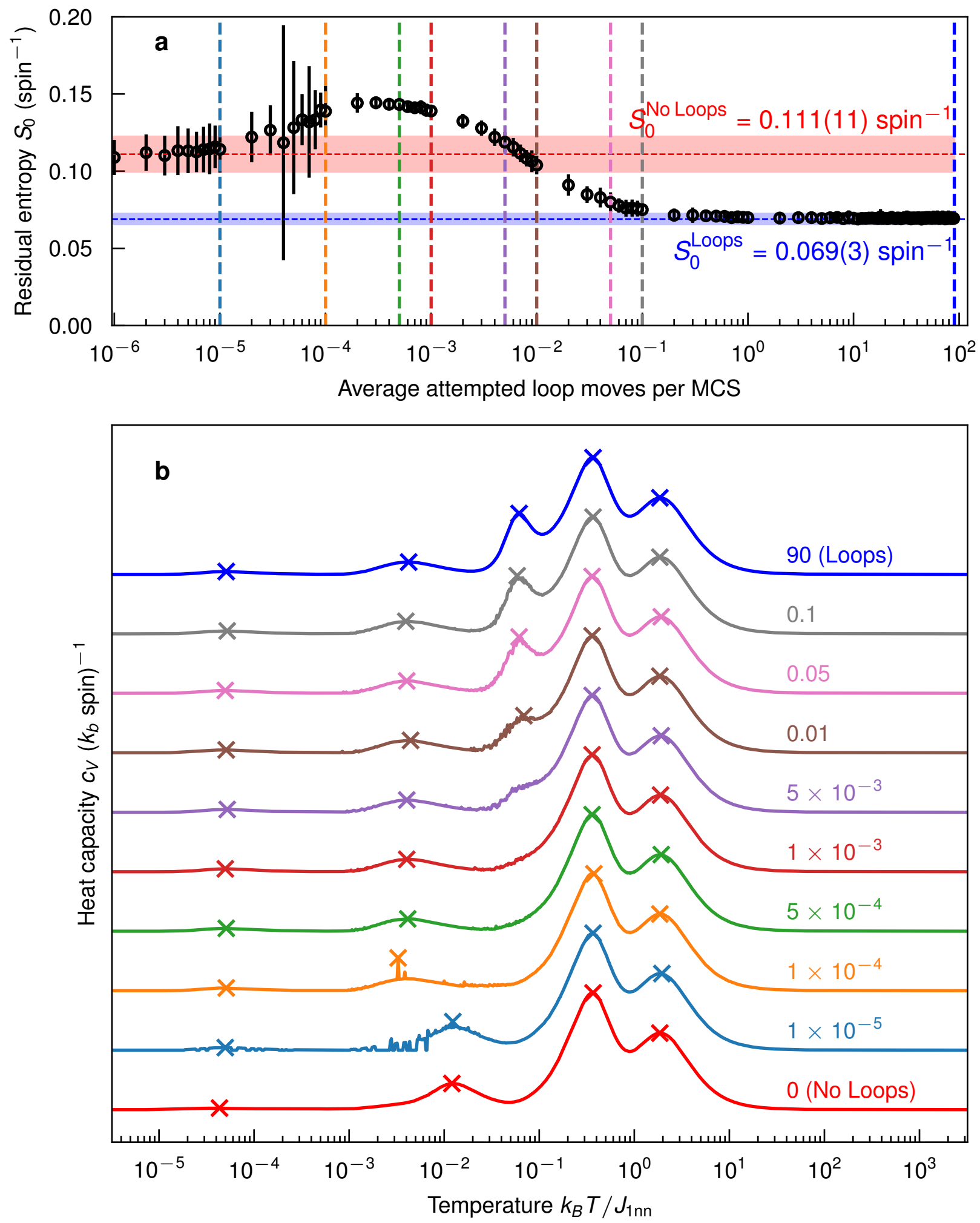

Figure S5. Required average number of attempted loop moves per Monte Carlo step to recover the thermal properties of the buckyball artificial spin ice. a, Dependence of the residual entropy on the average number of attempted loop moves per Monte Carlo step (MCS). The blue and red bands indicate the uncertainty in the average value (continuous line) for the two limiting cases of 'No Loops' and 'Loops', respectively. The average values of these limiting cases are noted explicitly. The position and error bars for each marker indicate the average and $\pm 1\sigma$ of 100 independent runs, respectively. **b**, Waterfall plot of the specific heat capacity as a function of temperature for different numbers of attempted loop moves per Monte Carlo step. The individual c_V curves are coloured according to the average number of attempted loop moves per Monte Carlo step, which correspond to the vertical lines in panel **a**. As the average number of attempted loop moves increases from 0 to 10^{-4} per Monte Carlo step, the peak that is initially near to $k_BT/J_{1nn} \approx 10^{-2}$ shifts to lower temperatures. Then, when more than 10^{-3} loop moves are attempted per Monte Carlo step, the peak associated with the crossover to the Pentagon sector forms at a temperature $k_BT/J_{1nn} \approx 0.061$. By 0.05 attempted loop moves per Monte Carlo step, sufficient loop moves are performed on average per Monte Carlo step to recover the expected five crossovers in the heat capacity.

Supplementary Note 2 Introduction of a cutoff in the dipolar interaction

As we alluded to in the main text, the five peaks in the specific heat capacity of the buckyball artificial spin ice are recovered only when all possible interactions are included. At this stage, it is instructive to consider what effect introducing a cutoff radius r_{cut} to the dipolar Hamiltonian, Eq. (S1), has on the thermal properties of the buckyball artificial spin ice.

There are 42 classes of nearest-neighbour spins in the buckyball artificial spin ice. These range in separation from $r_{1\text{nn}} \approx 0.81a$ (the distance between adjacent spins in a pentagonal face) to $r_{42\text{nn}} \approx 4.85a$ (by which point, all interactions are accounted for). In our simulations, we have set the edge length of our buckyball lattice equal to one, i.e. $a = 1$. For each possible cutoff, we have performed 100 independent simulations, with and without loop moves.

By changing the cutoff, we are able to tune the number, height and position of the peaks in c_{V}, as shown in Fig. S6 where the data for six representative cutoffs are displayed: three near to the spin in question (1nn, 2nn, 3nn) and three far-away (10nn, 20nn, 30nn). Ground state configurations for the nearby cutoffs are given in Fig. S7. These configurations show that both Spin-Ice and Charge-Ice sectors are driven by the short-range interactions. We know this because only ice-rule vertices are observed when first-nearest-neighbour interactions are included (Fig. S7**a**), and the same imperfect arrangement of charges is obtained as in the infinite case as soon as the cutoff is greater than or equal to the distance to the second-nearest-neighbours (Fig. S7**b**). Increasing the cutoff to the third–nearest-neighbour distance does not appear to qualitatively alter the ground-state configurations, compared with the case where only interactions up to the second–nearest neighbours are included (compare Fig. S7**c** with Fig. S7**b**). The similarity of the ground-state configurations in these two cases is consistent with the specific heat data: both the second- and third–nearest-neighbour cutoff cases exhibit two peaks in c_{V} as a function of temperature, although in the case of the third–nearest-neighbour, the two peaks almost overlap (Fig. S6**c**). By $r_{\text{cut}} = r_{10\text{nn}}$ (Fig. S6**d**), two peaks in the specific heat capacity are still present. However, the second peak has become clearly more pronounced.

While we do not show the behaviour of the charge-charge correlations in the ground state as a function of temperature, we have found that, once second-nearest-neighbour correlations are included, $\langle Q_i Q_j \rangle_{1\text{nn}}$ reaches the same value of $-11/15$ in the ground state as in the infinite cutoff case. For models with small r_{cut}, it appears that loop moves do not make much difference to the behaviour of the specific heat capacity since there is practically no difference between the blue ('Loops') and red ('No Loops') curves in any of the panels Fig. S6**a**-**d**. Indeed, only the $r_{\text{cut}} = r_{20\text{nn}}$ and $r_{\text{cut}} = r_{30\text{nn}}$ curves feature different low-temperature behaviour in the absence of loop moves (Fig. S6**e** and Fig. S6**f**).

This point is made explicit by plotting the residual entropy as a function of cutoff radius, as shown in Fig. S8**a**, for the case where loop moves are used (blue crosses) and where only single spin flips are used (red circles). The difference between these two residual entropies, $S^{\text{No Loops}} - S^{\text{Loops}}$, is given in Fig. S8**b**. Once the cutoff radius is greater than or equal to the distance to the sixth-nearest neighbours (vertical dashed line in Fig. S8**a**,**b**), the residual entropy of the 'No Loops' scenario is always higher than the residual entropy of the 'Loops' scenario, i.e. the difference in entropies is always greater than zero for $r_{\text{cut}} \geq r_{6\text{nn}}$. This means that loop moves are usually required to efficiently access the ground state of the buckyball artificial spin ice when the cutoff radius is greater than or equal to the distance to the sixth-nearest-neighbours, since some states can clearly not be accessed with only single spin flips. This behaviour is *unlike* that seen for in-plane artificial spin ices, where usually a small number of nearest-neighbour interactions are sufficient to recover the key aspects of the phase diagram.[3,4]

There remain a small number of cutoff radii beyond $r_{6\text{nn}}$ for which the curves of specific heat capacity obtained using loop moves and single spin flips, and using only single spin flips, nearly overlap. Figure S6**d**,

corresponding to $r_{\text{cut}} = r_{10\text{nn}}$, provides a representative example. Nevertheless, even in this case, the residual entropy in the 'No Loops' scenario is slightly higher than that in the 'Loops' scenario.

Finally, in Fig. S9, we show the locus of crossover temperatures as a function of the cutoff radius for simulations with loop moves (Fig. S9**a**) and without loop moves (Fig. S9**b**). As in the main text, the crossovers are located by identifying prominent peaks in c_{V}. This may mean that some features—shoulders or closely spaced maxima—are missed (c.f. Fig. S6**c**, where only the rightmost peak is easily identified). In each panel, the rightmost points correspond to the crossover temperatures of the full-dipolar model, i.e. when all interactions are included. In Fig. S9**a**, this results in five crossover temperatures at $r_{42\text{nn}} \approx 4.85a$ when both single–spin flips and loop moves are allowed, whereas in Fig. S9**b** only three crossover temperatures are observed at the same cutoff when only single spin flips are used. From inspection of the plots in Fig. S9, four features are readily apparent:

1. The crossovers to the Spin-Ice and Charge-Ice sectors are relatively insensitive to the choice of cutoff radius, once sixth-nearest-neighbours are included. We see this because the position of the two highest temperature peaks (upper two sets of blue crosses in Fig. S9**a** and upper two sets of red circles in Fig. S9**b**) do not change position once $r_{\text{cut}} \geq r_{6\text{nn}}$, so to the right of the vertical dashed line in both panels.

2. The temperatures at which the buckyball artificial spin ice crosses into the Spin-Ice and Charge-Ice sectors agree in both the 'Loop' and 'No Loops' cases. Comparing Fig. S9**a** and Fig. S9**b**, we see that, for each cutoff, the two highest crossovers occur at the same temperature in both panels. This reinforces claims made in the main text that the Spin-Ice and Charge-Ice sectors are driven by short-range interactions, since they appear at the same temperatures irrespective of whether loop moves or only single spin flips are included.

3. In the absence of loop moves, normally only one further crossover appears in the vicinity of $k_B T / J_{1\text{nn}} \approx 10^{-2}$ when $r_{\text{cut}} > 3a$ (lower set of red circles at low temperatures in Fig. S9**b**). This is consistent with our claim that the low-temperature sectors can only be accessed via collective rearrangements of the spins.

4. When loop moves are used, the exact position of the low-temperature crossovers does not tend towards a universal value as more neighbours are included. This contrasts with conventional 2D artificial spin ices where increasing the interaction cutoff in the dipolar Hamiltonian leads to crossover temperatures that approach well-defined values. Consequently, only a finite number of nearest-neighbour interactions is typically required to capture the salient features of the phase diagram for 2D artificial spin ices.

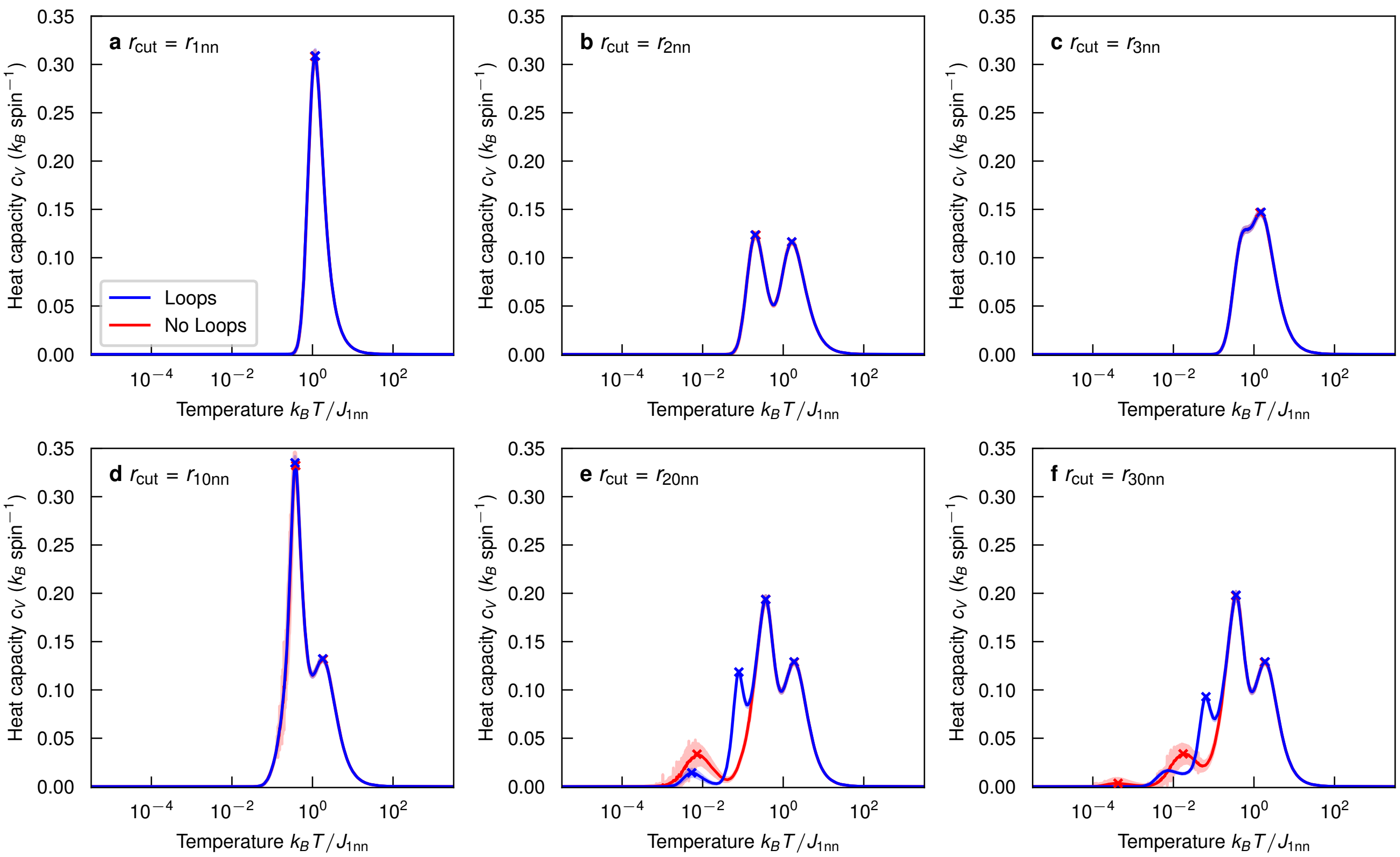

Figure S6. Effect on the heat capacity of dipolar interaction cutoffs. The number, position and height of peaks in the heat capacity differ as the cutoff r_{cut} for the dipolar interaction is varied. Examples include: **a**, a single peak when $r_{\text{cut}} = r_{1\text{nn}}$; **b**, two peaks of approximately equal height when $r_{\text{cut}} = r_{2\text{nn}}$; **c**, a broad peak with shoulder when $r_{\text{cut}} = r_{3\text{nn}}$; and **d**, two peaks of unequal height and width when $r_{\text{cut}} = r_{10\text{nn}}$. When further-out neighbours are included, as in **e**, $r_{\text{cut}} = r_{20\text{nn}}$ and **f**, $r_{\text{cut}} = r_{30\text{nn}}$, the effect of loop moves becomes noticeable. All curves reflect the average of 100 independent runs. The blue and red curves correspond to the average heat capacity taken from simulations with and without loop moves, respectively. Shaded regions demarcate $\pm 1\sigma$ around the average of 100 independent runs in each case.

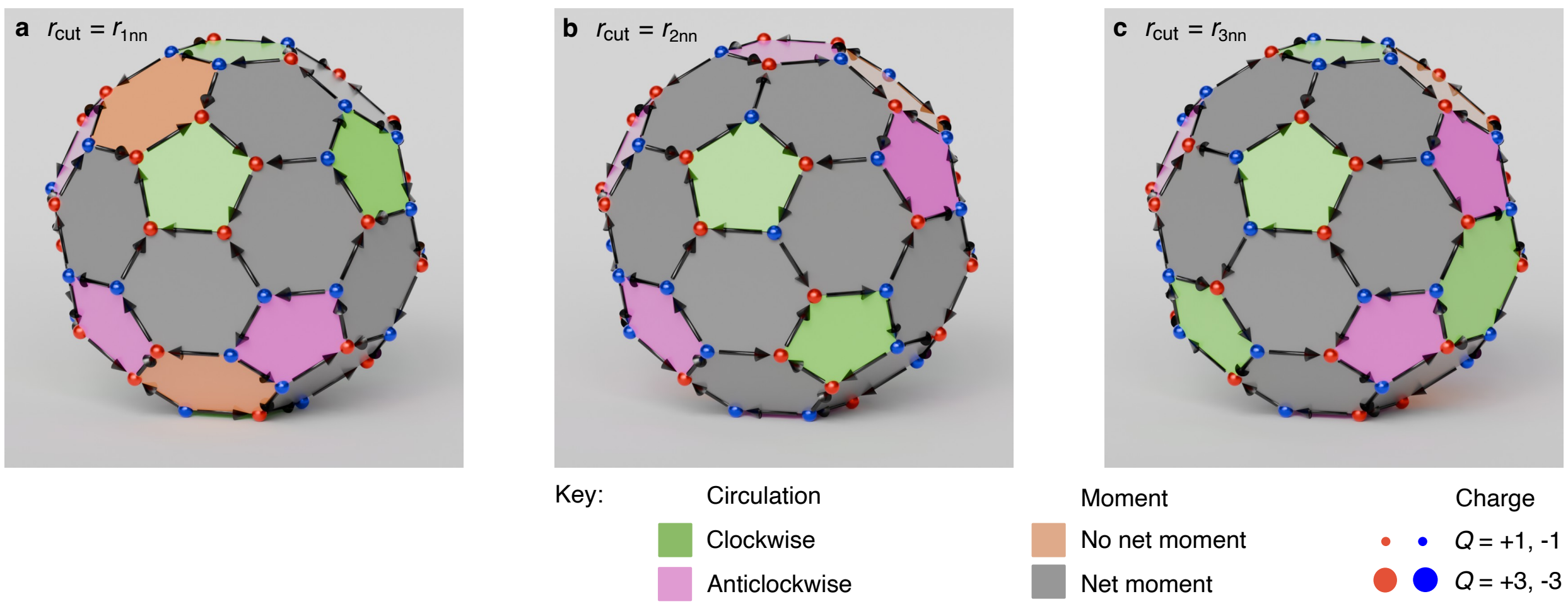

Figure S7. Example spin configurations at $T = 0$ for small cutoff radii. **a**, $r_{\text{cut}} = r_{1\text{nn}}$, where all the pentagons have flux-closed, but not necessarily in the same sense. **b**, $r_{\text{cut}} = r_{2\text{nn}}$, where a semblance of charge order has begun to develop, so that each $Q = +1$ vertex tries to surround itself with three $Q = -1$ vertices, and vice versa. As in the main text, we emphasise that perfect charge order cannot be accommodated on the surface of the buckyball. However, for a cutoff $r_{\text{cut}} = r_{2\text{nn}}$, the value of the nearest-neighbour charge correlator $\langle Q_i Q_j \rangle_{1\text{nn}}$ reaches in the ground state the same value of $-11/15$ as for the infinite cutoff. **c**, $r_{\text{cut}} = r_{3\text{nn}}$, where some hexagons also flux-close, but long-range spin order is still not present. The directions of each spin as well as the charge at each vertex are shown. Faces are coloured according to the circulation and net moment of their associated spins, as indicated by the key, which matches that used in main text, Fig. 2. Both loop moves and single spin flips were used during the simulated anneals to access these configurations.

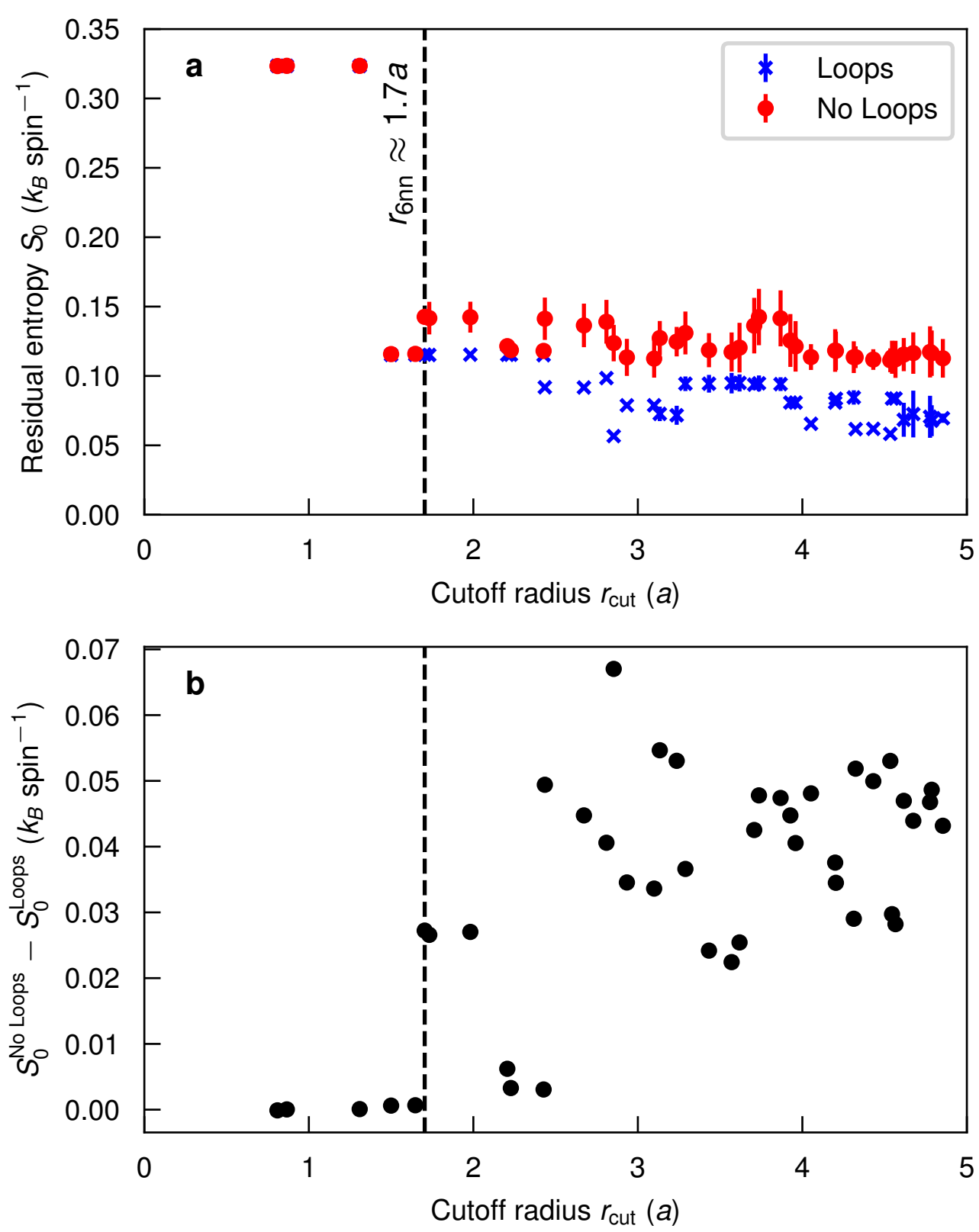

Figure S8. Effect on the residual entropy S_0 of applying different cutoffs to the dipolar interaction. **a**, Change of residual entropy for simulations with and without loop moves (blue crosses and red circles, respectively). Error bars reflect $\pm\,1\sigma$, when the quantity is averaged over 100 independent runs. **b**, Difference between these two cases, i.e. $S_0^{\rm No\,Loops} - S_0^{\rm Loops}$. Loop moves make a difference to the magnetic configuration only when the cutoff radius is greater than or equal to the distance to the sixth-nearest neighbour $r_{\rm 6nn} \approx 1.7a$ (indicated by the dashed black vertical line in both panels).

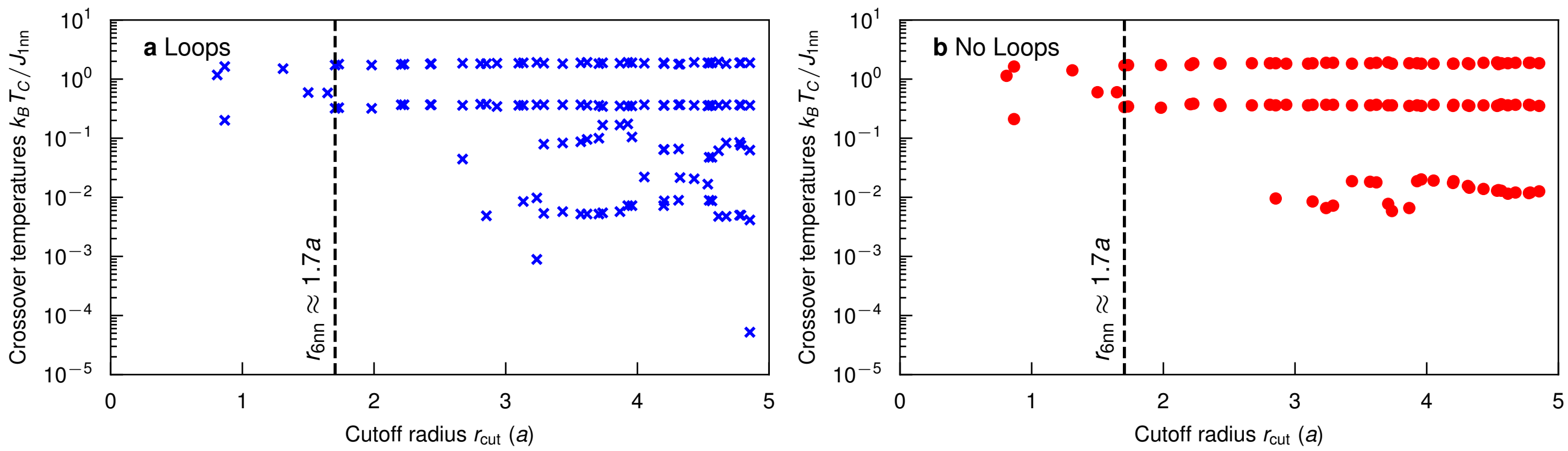

Figure S9. Effect on crossover temperatures of applying a cutoff to the dipolar interaction for simulated anneals with and without loop moves. The locus of system-wide crossover temperatures as a function of the cutoff radius for simulated anneals: **a**, with loop moves, blue crosses; and **b**, without loop moves, red circles. The crossover temperatures are extracted from peaks in the heat capacity, as in the main text. The positions of the high-temperature crossovers, i.e. those associated with the crossovers to the Spin-Ice and Charge-Ice sectors (upper two sets of markers), appear largely insensitive to changes in the cutoff radius once sixth-nearest-neighbours are included (indicated by the dashed black vertical line in both panels). The number and positions of the low-temperature peaks, which mark the onset of some form of spin ordering, do change with cutoff radius, however. For the case of simulated anneals with loop moves, spin ordering can happen in one, two or three steps as more interactions are considered. The rightmost set of markers in **a**, located at $r_{42\text{nn}} \approx 4.85a$, corresponds to the situation used in the main text, where all dipolar interactions are included, resulting in a five-stage ordering to the ground state. For the case of simulated anneals without loop moves, there tends to be only one further crossover after the transition to the charge-ice regime, which occurs at $k_B T / J_{1\text{nn}} \approx 10^{-2}$. Its position does not vary much once $r_{\text{cut}} \geq 4a$. All data points reflect the average position of a peak for 100 independent measurements of the heat capacity.

Supplementary Note 3 Dipolar Needle Approximation

In the main text and elsewhere in the Supplementary Information, we have approximated the spins in our buckyball artificial spin ice as point dipoles, which are coupled through the dipolar interaction [Eq. (S1)]. In a real artificial spin ice, the nanomagnets are not points, but rather extended objects that sit close to each other. This suggests that the point dipolar approximation is modified, principally in the near-field. In this section, we examine to what extent a dipolar needle approximation, which better mimics a mesoscopic magnet, affects the thermal properties of the buckyball artificial spin ice.

The point dipolar approximation provides good agreement with experiment in a range of two-dimensional artificial spin ices; see Refs. 5–7 for its application to the square, kagome and pinwheel systems. There exist various other schemes to more realistically model the finite extent of nanomagnets in an artificial spin ice, including a full micromagnetic treatment based on either a finite difference or finite element approach[8,9]. An intermediate approach between this micromagnetic treatment and the dipolar approximation is to treat the spins as 'dipolar needles', so including the notion of length.[10] In this framework, the nanomagnets are approximated as two equal but opposite magnetic charges, separated by a length l, such that the energetics are governed by their mutual Couloumb interaction. This approach is sometimes referred to as the 'charge dumbbell' approximation and the length of the dipolar needle is a parameter by which the interactions in the system can be varied. Increasing this length l—in effect pushing the charges associated with adjacent spins closer together—significantly increases the first-nearest-neighbour interaction, for example.

We now repeat our Monte Carlo simulations, approximating the spins as dipolar needles with lengths l/a in the range $[0.001, 0.999]$. The dependence of the residual entropy S_0 on the needle length is shown in Fig. S10. Error bars reflect $\pm 1\sigma$ about the mean of 100 independent runs. The blue band indicates the $\pm 1\sigma$ about the average point dipolar value of $0.069(3)k_B$ per spin. Most of the values for the residual entropy still lie within this band. Only in the extreme cases ($l/a < 0.05$ or $l/a > 0.95$), shaded in grey, does S_0 lie significantly outside the $\pm 1\sigma$ band. In some cases, this is because the chosen temperature set was insufficient to capture all the peaks in the heat capacity. As l/a is varied, the locations of the crossovers also shift (not shown), since any crossover temperature T_{Cross} is set indirectly through the hierarchy of interactions in the system. To achieve strongly coupled systems, typical experimental values of l/a for 2D artificial spin ices are close to 1, e.g. 0.86[6] to 0.94[11]. Given that the dipolar needle simulations predict the same residual entropy as for the point dipole simulations, it appears we reach the same type of magnetic configuration in both cases. As a result, we conclude that the ground state, with its ensuing topological defects, is the same irrespective of the model used. Provided that a sufficiently good thermal anneal can be performed, the success of the dipolar needle approximation leads us to think that such a ground state incorporating topological defects should form in real samples, assuming that dynamical freezing can be overcome.[12]

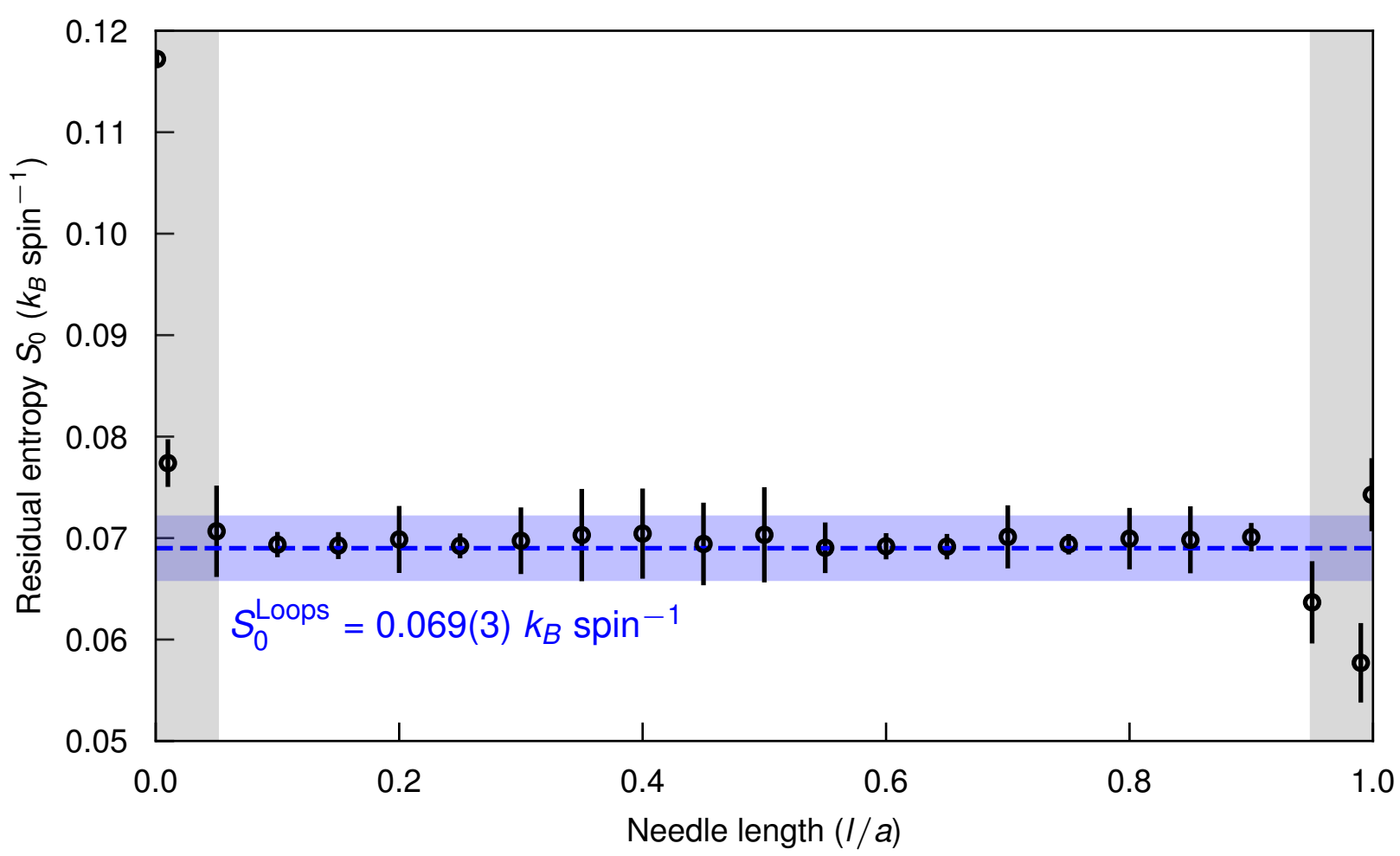

Figure S10. Residual entropy as a function of needle length l/a using the dipolar needle approximation. The shaded-grey regions ($l/a < 0.05$ or $l/a > 0.95$) highlight the extreme cases for which the chosen temperature set in the Monte Carlo simulations was insufficient to record all peaks in the heat capacity. As a result, our estimates of the residual entropy in those regions are unlikely to be accurate. The blue dashed line indicates the average value of $S_0 = 0.069(3)k_B$ per spin and the blue horizontal band indicates its $\pm 1\sigma$ uncertainty obtained with the point dipolar Hamiltonian. Most of the values of the residual entropy lie within this blue horizontal band, which suggests that the magnetic configurations reached at $T = 0$ using the more nuanced dipolar needle approximation are the same as those in the point dipolar framework. This suggests, also, that the broad features of the thermal properties themselves are the same when calculated in both models. For each marker, its position and associated error bar indicate the mean and $\pm 1\sigma$ of 100 independent runs.

Supplementary Note 4 Temperature Dependence of Vertex Populations

In the main text, we remarked that the highest temperature crossover, at $k_B T/J_{1\text{nn}} \approx 1.85$, represents a crossover to a Spin-Ice sector, where only $Q = \pm 1$ Type 1A and 1B vertices are observed. Fig. S11, which shows the change in fractional vertex populations with temperature, confirms this. The fractional population of a given vertex type is defined as the number of vertices of that type divided by the total number of vertices in the buckyball (60). Three curves corresponding to the three vertex types are plotted: high-energy $Q = \pm 3$ Type 2 vertices, in green; $Q = \pm 1$ Type 1B vertices, in orange; and the lowest energy $Q = \pm 1$ Type 1A vertices in blue. As a reminder, both Type 1A and Type 1B vertices are ice-rule-obeying configurations, but differ in whether the spins at a vertex associated with the pentagonal face are in a low-energy or high-energy arrangement, respectively.

At high temperatures, the fractional populations correspond to the degeneracy of each vertex, so that half of the vertices are $Q = \pm 1$ Type 1A, one quarter are $Q = \pm 1$ Type 1B, and the remaining quarter are $Q = \pm 3$ Type 2 vertices. On decreasing the temperature, high-energy $Q = \pm 3$ Type 2 vertices disappear in the vicinity of the crossover to the Spin-Ice sector. Both types of ice-rule vertices initially increase in number with decreasing temperature to compensate. However, as the temperature is further decreased, the population of Type 1B vertices rapidly decreases, vanishing at the crossover to the Charge-Ice sector. Below the crossover to the Charge-Ice sector, $k_B T/J_{1\text{nn}} \approx 0.360$, only Type 1A vertices are present in the buckyball artificial spin ice.

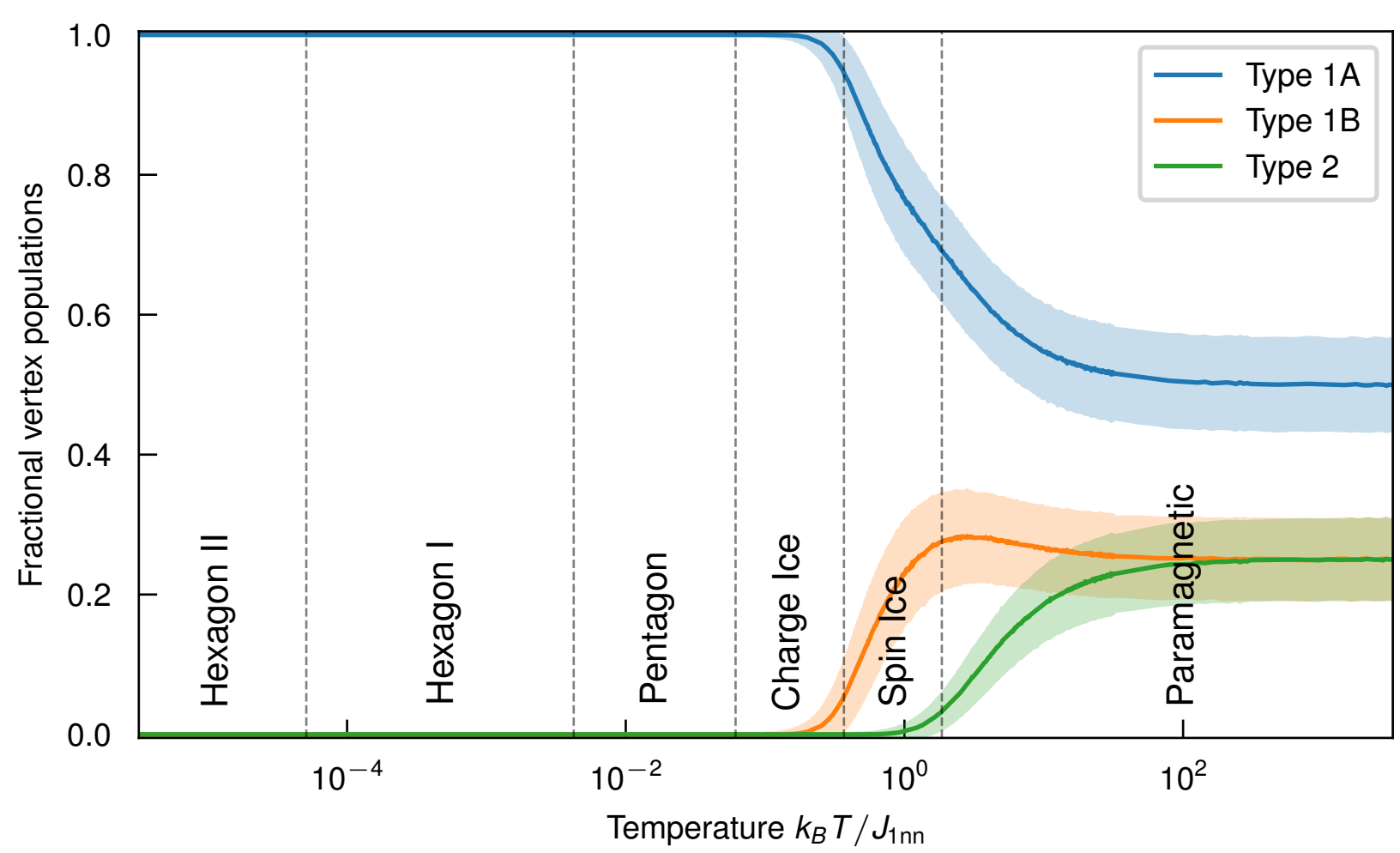

Figure S11. Temperature dependence of ice-rule-obeying and high-energy vertex populations. The behaviour of the three vertex types as a function of temperature is displayed: on cooling, high-energy $Q = \pm 3$ Type 2 vertices (green curve) disappear in the vicinity of the crossover to the Spin-Ice sector; $Q = \pm 1$ Type 1B vertices (orange curve) disappear at the crossover to the Charge-Ice sector; and $Q = \pm 1$ Type 1A vertices populate the entire lattice in the Charge-Ice and lower-temperature sectors. The five vertical lines indicate the temperature of the five crossovers for the buckyball artificial spin ice. Shaded regions demarcate $\pm 1\sigma$ around the average of 5000 independent runs.

Supplementary Note 5 Loop Structure in the Low-Temperature Sectors

In the main text, we noted that the crossovers to the low-temperature sectors are not associated with changes in the loop structure of the spins of the buckyball artificial spin ice. By this, we mean that these sectors do not result from the formation of different types of closed loops (e.g. longer or shorter loops, or changes in the overall loop population) in the buckyball artificial spin ice, although the specific loop structure will, of course, vary from configuration to configuration.

In Fig. S12, we provide evidence for this by displaying the temperature dependence of four quantities that characterise the loop population: in **a**, the probability that a randomly-chosen spin is a member of at least one closed loop; in **b**, the average number of loops; and, in **c**, both the average length and maximum length of the loops. For these purposes, a loop is defined by its constituent set of head-to-tail spins, irrespective of the starting spin as defined in the Monte Carlo routine. In each panel of Fig. S12, the five dashed vertical lines indicate the five system-wide crossovers between the different sectors in which the buckyball artificial spin ice can be found, which are labelled explicitly in Fig. S12**a**.

In the Paramagnetic sector, the probability that a spin belongs to at least one closed loop is small, as can be seen in Fig. S12**a**. More loops start to form at a temperature slightly above the crossover to the Spin-Ice sector. Consequently, the probability that a randomly chosen spin is a member of at least one loop $\mathrm{P(Loop)}$ increases. This increase in $\mathrm{P(Loop)}$ continues as the temperature decreases and, after the crossover to the Charge-Ice sector, every spin belongs to at least one closed loop (Fig. S12**a**), i.e. $\mathrm{P(Loop)} = 1$.

The behaviour of the average number of loops is more nuanced (Fig. S12**b**): on decreasing the temperature, they increase in number, reaching a maximum in the middle of the Charge-Ice sector, before falling back to a lower value at the crossover to the Pentagon sector. There is little change in the number of loops below this temperature, with the ground state featuring on average 519 ± 5 loops. The decrease in the number of loops beginning in the middle of Charge-Ice sector may be similar to the effect observed in computational studies of dipolar spin ice, where the acceptance rate of loop moves decreases as long-range spin order develops[2]. In the buckyball artificial spin ice, the crossover to the Pentagon sector marks the first temperature at which there is some form of system-spanning spin order corresponding to the flux closure of all the spins associated with pentagonal faces. This cooperative effect will naturally place some constraints on the loop population. Therefore, analogous to the decrease in the acceptance rate of loop moves in dipolar spin ice noted in Ref. 2, we would expect the number of loops to decrease near to the crossover to the Pentagon sector—exactly as we observe in Fig. S12**b**. Similarly, as can be seen in Fig. S12**c**, neither the average loop length nor the maximum loop length changes much below the crossover to the Pentagon sector. Interestingly, the maximum loop length in the ground state of 57.4 ± 1.2 spins involves approximately two-thirds of the 90 spins of the buckyball artificial spin ice.

We have thus established that the low-temperature peaks in the heat capacity of the buckyball artificial spin ice are not associated with any change in aggregate quantities describing the loop structure in the lattice. Rather, as we demonstrated in the main text, the three low-temperature crossovers correspond to the ordering of three separate subsets of spins: those surrounding the pentagonal faces, those on the hexagons forming an equatorial belt around the buckyball, and those on the hexagonal faces on either side of this belt.

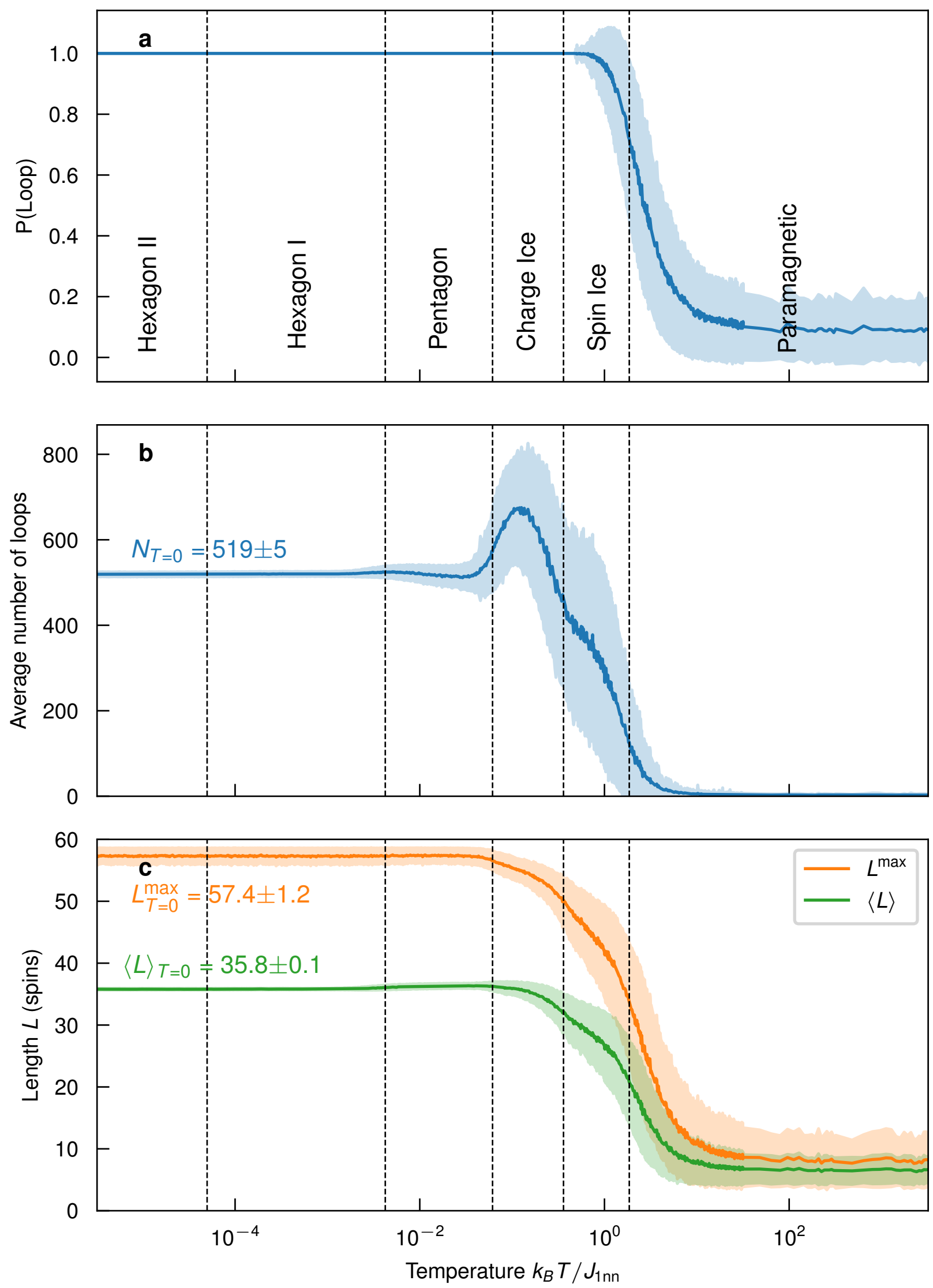

Figure S12. Loop statistics as a function of temperature. a, Probability that a randomly chosen spin belongs to at least one loop of head-to-tail spins as a function of temperature. As the temperature goes below the crossover to the Charge-Ice sector, each spin appears to belong to at least one loop since $P(\text{Loop}) = 1$. **b**, Number of loops as a function of temperature. On decreasing the temperature, the number of loops peaks after the crossover to the Charge-Ice sector, before decreasing. On decreasing the temperature further, the number of loops remain approximately constant when passing through each of the Pentagon, Hexagon I, and Hexagon II sectors. The $T = 0$ configuration hosts 519 ± 5 loops, as labelled. **c**, The maximum loop length L^{max} (orange) and average loop length $\langle L \rangle$ (green) as a function of temperature. The average values in the ground state and their standard deviation are given. In all panels, the lines and shaded regions indicate the average and $\pm 1\sigma$ of 500 independent simulations, respectively. For each simulated anneal, at each temperature point, the loop population is sampled by attempting 10^8 loop moves and recording the relevant quantities if the move is accepted.

Supplementary Note 6 Comparison of Spin Freezing with and without Loop Moves

In the main text, we discussed how partial spin order emerges in the buckyball artificial spin ice when certain subsets of spins freeze near to the three low-temperature crossovers. This conclusion was based on examining the expectation value of each spin $\langle \mathbf{s}_i \rangle$. This quantity depends on the specific dynamics employed for the Monte Carlo simulations, in particular, whether loop moves are included. This is because loop moves allow collective fluctuations to continue at low temperatures where single spin flips alone would be rejected. In Fig. S13, we compare simulations of the spin freezing behaviour reported in the main text—where both single spin flips and loop moves were used—to simulations in which only single spin flips are employed. The left column (labelled 'Loops') displays data from simulations using both single spin flips and loop moves, matching the results presented in the main text. The right column (labelled 'No Loops') displays data from simulations using single spin flips only.

Supplementary Note 6.1 Single Spin Flips and Loop Moves ('Loops')

As discussed in the main text, when both single spin flips and loop moves are used in Monte Carlo simulations, the specific heat capacity of the buckyball artificial spin ice exhibits five crossovers, as shown in Fig. S13**a**. The temperatures corresponding to these five peaks are indicated by the dashed vertical lines. The curve of specific heat capacity against temperature in Fig. S13**a** is identical to that shown in the Fig. 2**c** of the main text and to the blue curve in Fig. S2**a**.

In this case, the spins in the buckyball artificial spin ice can be divided into three groups, based on the behaviour of $\langle \mathbf{s}_i \rangle$. This is illustrated in Fig. S13**b**, where we plot the temperature dependence of $\langle \mathbf{s}_i \rangle$ for each spin as a faint blue trace. When many spins behave similarly, the overlapping traces produce a darker colour. In Fig. S13**c**, we show 3D representations of $\langle \mathbf{s}_i \rangle$ and the average vertex charge $\langle Q_i \rangle$ at the four temperatures indicated in Fig. S13**b**. In these snapshots, the length of arrow 'i' is proportional to $\langle \mathbf{s}_i \rangle$, and the size of the sphere representing vertex 'i' is proportional to $\langle Q_i \rangle$. The colour of the sphere represents the sign of the average charge: red (blue) if $\langle Q_i \rangle > 0$ ($\langle Q_i \rangle < 0$). Because we have selected snapshots where some spins are frozen and others are still fluctuating, the spins and charges tend to appear either full-sized or very small. As a reminder, the quantities $\langle \mathbf{s}_i \rangle$ and $\langle Q_i \rangle$ denote the time-averaged spin moment and vertex charge, respectively, and are computed by averaging over the number of Monte Carlo steps performed at a given temperature.

In order of decreasing temperature, the four snapshots in Fig. S13**c** are:

- Panel ①, in the Paramagnetic sector, when none of the spins have ordered;
- Panel ②, below the crossover to the Pentagon sector, when the 60 spins around the 12 pentagonal faces freeze into closed loops with the same circulation;
- Panel ③, below the crossover to the Hexagon I sector, when a further 20 spins freeze on 10 hexagonal faces, forming a belt of flux-closed faces around the equator of the buckyball, which features the maximal number of flux-closed faces; and
- Panel ④, below the final crossover to the Hexagon II sector, when the remaining spins freeze in the hexagons surrounding the central flux-closed pentagon at each pole.

These snapshots of $\langle \mathbf{s}_i \rangle$ and $\langle Q_i \rangle$ are analogous to those presented in Fig. 4**b** of the main text, except we do not colour the faces.

Supplementary Note 6.2 Only Single Spin Flips ('No Loops')

When the Monte Carlo simulations feature only single spin flips, the specific heat capacity of the buckyball artificial spin ice exhibits four distinct peaks, as seen in Fig. S13**d**. The temperatures corresponding to these four peaks are indicated by the dashed vertical lines. The behavior of the specific heat capacity in the 'No Loops' scenario is discussed in more detail in Supplementary Note 1.2. Specifically, the curve of specific heat capacity against temperature in Fig. S13**d** is identical to the red curve in Fig. S2**a**. While we have not exhaustively classified the magnetic sectors that emerge with only single spin-flip dynamics, we expect that the two highest-temperature sectors correspond to a Paramagnetic sector and a Spin-Ice sector. This is supported by the fact that the temperature dependence of the specific heat capacity is identical for simulations with and without loop moves down to $k_B T/J_{1\text{nn}} \approx 2\times10^{-1}$, as shown in Fig. S2**a**.

The behavior of the average spin moment $\langle \mathbf{s}_i \rangle$ also differs from the case where loop moves are enabled (see Fig. S13**e**). In the absence of loop moves, the 90 spins in the system separate into two groups with distinct freezing behaviors:

- The first group consists of 60 spins located on the 12 pentagonal faces. These spins freeze over a narrow temperature window centered around $k_B T/J1\text{nn} \approx 2\times10^{-1}$.
- In the second group, the remaining 30 spins freeze gradually over a broader temperature range, starting near $k_B T/J_{1\text{nn}} \approx 10^{-1}$ and finishing by $k_B T/J_{1\text{nn}} \approx 2\times10^{-3}$.

To illustrate this progressive freezing, we have highlighted four representative temperatures in Fig.S13**e**, with corresponding snapshots of $\langle \mathbf{s}_i \rangle$ and $\langle Q_i \rangle$ shown in Fig.S13**f**. In order of decreasing temperature, these snapshots correspond to:

- Panel ①, at $k_B T/J_{1\text{nn}} \approx 10^{-1}$, when the 60 spins on the pentagonal faces freeze into closed loops with uniform circulation;
- Panel ②, at $k_B T/J_{1\text{nn}} \approx 4\times10^{-2}$, where a subset of the remaining 30 spins begin to freeze, resulting in smaller sized arrows in the snapshot;
- Panel ③, at $k_B T/J_{1\text{nn}} \approx 1\times10^{-2}$, by which point an additional 10 spins have fully frozen; and
- Panel ④, at $k_B T/J_{1\text{nn}} \approx 1\times10^{-3}$, where all 90 spins have frozen, yielding the configuration shown in more detail in Fig. S4**a**, where the faces are coloured to indicate the circulation or net moment of each face.

Similar to the simulations with loop moves, we observe that all spins on the pentagonal faces freeze simultaneously at a common temperature. However, this freezing occurs at a higher temperature when only single spin flips are used.

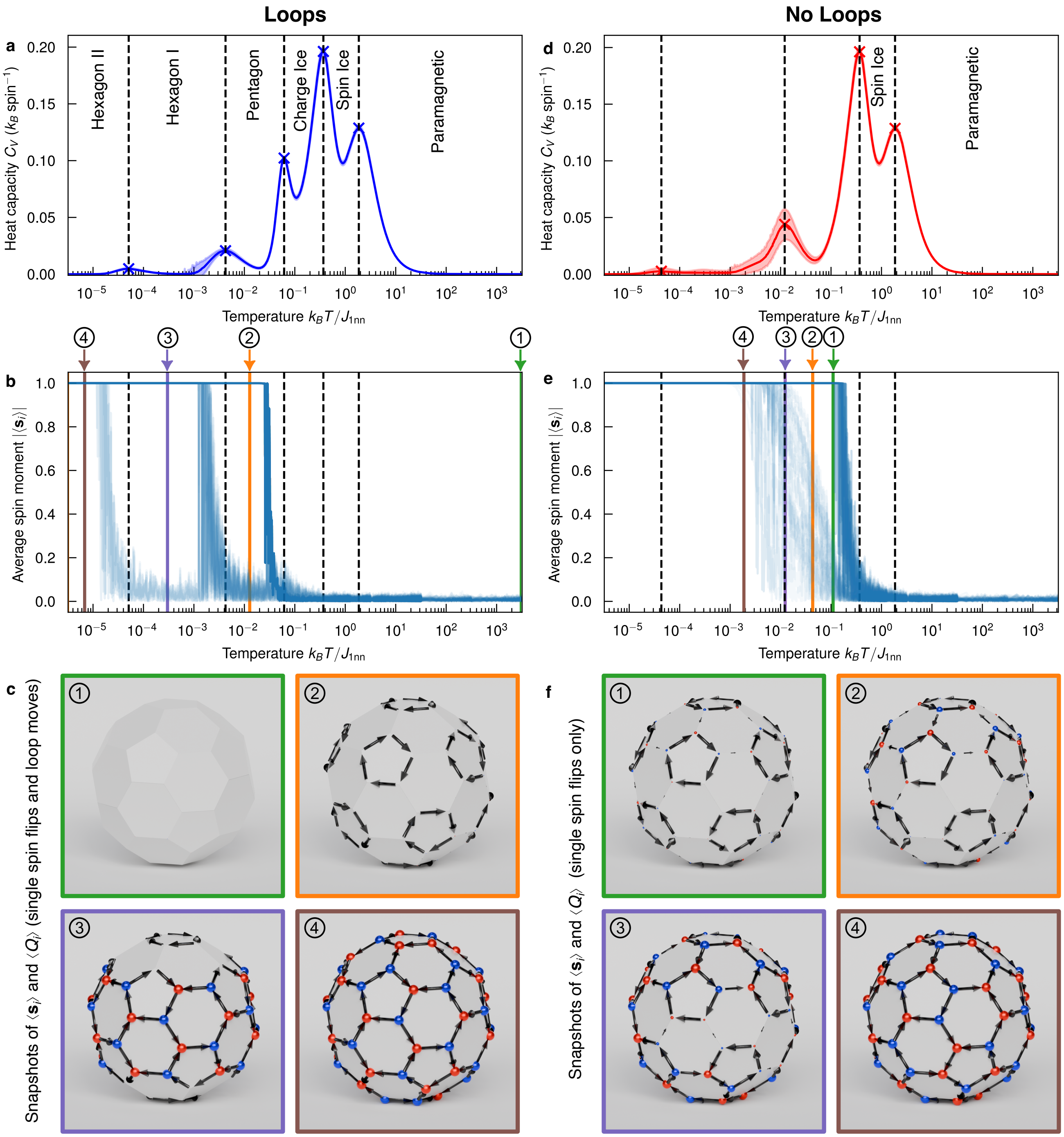

Figure S13. Effect of loop moves on spin freezing in the buckyball artificial spin ice during simulated annealing. The left column shows results from simulated anneals where both single spin flips and loop moves are allowed (as in Fig. 2 and Fig. 4 of the main text). The right column shows results from simulated anneals using only single spin flips, with loop moves disabled. The top and second rows show **a**,**d** the temperature dependence of the specific heat capacity, c_V, and **b**,**e** average spin moment, $\langle \mathbf{s}_i \rangle$, for each spin in the lattice. Without loop moves, the characteristic low-temperature three-stage spin ordering is no longer observed (panel **e**). The 60 spins on the 12 pentagonal faces still freeze just below the crossover to the Pentagon sector. However, the remaining 30 spins freeze gradually over a broad temperature range, beginning around $k_BT/J_{1nn} \approx 10^{-1}$ and completing by $k_BT/J_{1nn} \approx 2 \times 10^{-3}$.

Supplementary Note 7 Degeneracies of the Buckyball Artificial Spin Ice

In the main text, we remarked that the buckyball artificial spin ice has 480 ground states, resulting in a residual entropy of $S_0 \approx 0.0695\,k_B$ per spin. In this section, we discuss how this number of ground states is obtained and how their spin configurations can be related to each other. To help our discussion, we note some features of the ground-state motifs depicted in the main text, Fig. 4c:

1. All 12 pentagonal faces are flux-closed in the same sense, in this example clockwise and are therefore coloured green according to the key.
2. Of the 20 hexagonal faces, seven are flux-closed, all in the opposite sense to the pentagons, i.e., anticlockwise in this example and are therefore coloured pink. Five of these anticlockwise flux-closed hexagons are located around the equator, while the remaining two occur at the bottom pole.
3. Considering just the equatorial motif, the five flux-closed hexagons occupy the upper row of hexagons along the equator. The five hexagons in which the constituent spins have neither net moment nor circulation are arranged along the lower row; these faces are coloured orange.
4. Of the hexagons belonging to the top pole, three have a net moment (shown in grey) and two have no net moment nor circulation. The top pole is situated on the same side of the equator that hosts the anticlockwise hexagons.
5. Of the hexagons belonging to the bottom pole, three have a net moment and two are flux-closed. We colour the flux-closed faces pink, which is used to indicate anticlockwise spin circulation when viewing the buckyball from the outside. In the sub-panel of Fig. 4**c**, the spins circulate clockwise because we are viewing the bottom pole from the centre of the buckyball, looking down. The bottom pole sits on the same side of the equator as the equator hexagons that exhibit neither a net moment nor circulation.

To generalise the last two points: when a pole contains flux-closed hexagons, it lies on the same side of the equator that contains hexagons with no net moment; and, vice versa, when a pole contains hexagons with no net moment, it lies on the side of the equator that contains flux-closed hexagons.

The 480 distinct ground states are related by a combination of global and local spin rearrangements that map one ground state onto another as described below.

In Figs. S14 and S15, the black arrow in the bottom right of the equator sub-panels points from the centre of the bottom polar pentagon to the centre of the top polar pentagon. The circled dots in the pole sub-panels then indicate this same direction when viewed from above.

Global Transformations Connecting Ground States

There are four global transformations, shown in Fig. S14, that connect ground states in the buckyball artificial spin ice. By global transformation, we mean an operation in which every spin and vertex charge in the buckyball artificial spin ice can be mapped to a new spin and vertex charge, resulting in a new ground state. We describe these transformations in turn, indicating in brackets the factor by which each contributes to the ground-state degeneracy.

1. Orientational Transformation (Fig. S14a): There are six distinct orientations of the buckyball, each defined by the axis that passes through the centres of two diametrically opposite pentagons ($\times 6$). A given ground state, which has two poles, can therefore be aligned such that the axis connecting the polar pentagons coincides with any one of these six axes.

2. Rotational Transformation (Fig. S14b): For a fixed orientation, additional magnetic ground states can be generated from a given one by applying discrete rotations about the symmetry axis. In particular, rotations by integer multiples of 72°, which corresponds to the central angle of a pentagon, map one ground state onto another ($\times 5$).

3. Time-Reversal Transformation (Fig. S14c): The system also exhibits an overall Ising symmetry: globally reversing all spins in the buckyball inverts the sense of spin circulation around all flux-closed faces and the net moment of all polarized hexagons ($\times 2$). This transformation, $\mathbf{s}_i \rightarrow -\mathbf{s}_i$, generates a new ground state from an existing one because the Hamiltonian has time-reversal symmetry.

4. Polar-Exchange Transformation (Fig. S14d): Finally, ground states can be connected by an operation that exchanges the top and bottom poles, provided that the flux-closed hexagons and the hexagons with no net moment in the equator are also exchanged ($\times 2$). While we refer to this as a *polar-exchange* transformation, it is important to note that the buckyball solid does not have mirror symmetry about the equatorial plane. As a result, this operation does not simply correspond to swapping spins on the top and bottom halves. Rather, to connect this to a genuine geometric symmetry of the underlying buckyball solid, it should be viewed as a half-turn rotation about an axis passing through the midpoints of two spins located opposite each other along the equator, as shown in the sub-panel of Fig. S14**d**, labelled "Example axis". In this sub-panel, the rotation is indicated by the yellow circular arrow, while the spins through which the axis passes are highlighted in red.

Together, these four transformations contribute a factor of $6 \times 5 \times 2 \times 2 = 120$ to the ground-state degeneracy.

Local Transformations Connecting Ground States

There exist four equivalent ground-state configurations, shown in Fig. S15. The leftmost panel, Fig. S15**a**, corresponds to the configuration presented in Figs. 2 and 4 of the main text. In the remaining three panels, the spins that are reversed relative to Fig. S15**a** are highlighted in yellow. Below, we briefly describe how each configuration differs from Fig. S15**a**.

Fig. S15b: A new ground state is obtained by reversing four spins located in the bottom pole, while keeping both the top pole and the equator unchanged. This transformation is equivalent to rotating the bottom pole by 72° counterclockwise, leaving the remainder of the structure unaltered.

Fig. S15c: This configuration is produced by reversing two specific spins, one in each pole. In the top pole, the reversed spin is that spin shared by the two hexagons that carry a net moment; reversing it changes the direction of the net moments in these two faces. In the bottom pole, the reversed spin is a spin shared between a flux-closed hexagonal face and a hexagonal face with a net moment. Reversing this spin exchanges the flux-closed hexagonal face with the net-moment hexagonal face.

Fig. S15d: This ground state is obtained by reversing six spins: one in the top pole (the same spin flipped in Fig. S15**c**) and five in the lower pole. In the lower pole, these are the five spins whose edges adjoin the central flux-closed pentagonal face but are not themselves part of it. The reversal of the spin in the top pole is the same as the transformation of the top pole shown in Fig. S15**c**. Reversing the five spins in the bottom pole modifies the arrangement of the hexagons carrying a net moment.

From these configurations, a clear relationship emerges:

- The configurations in Figs. S15**a** and S15**b** share the same top-pole and equator motifs, with the bottom pole in Fig. S15**b** rotated 72° anticlockwise relative to Fig. S15**a**.

- Similarly, the configurations in Figs. S15**c** and S15**d** share the same top-pole and equator motifs, with the bottom pole in Fig. S15**d** rotated 72° anticlockwise relative to Fig. S15**c**.

Multiplying these contributions yields a total ground-state degeneracy of:

$$4 \text{ configurations} \times (120 \text{ transformations}) = 480 \text{ configurations.} \tag{S3}$$

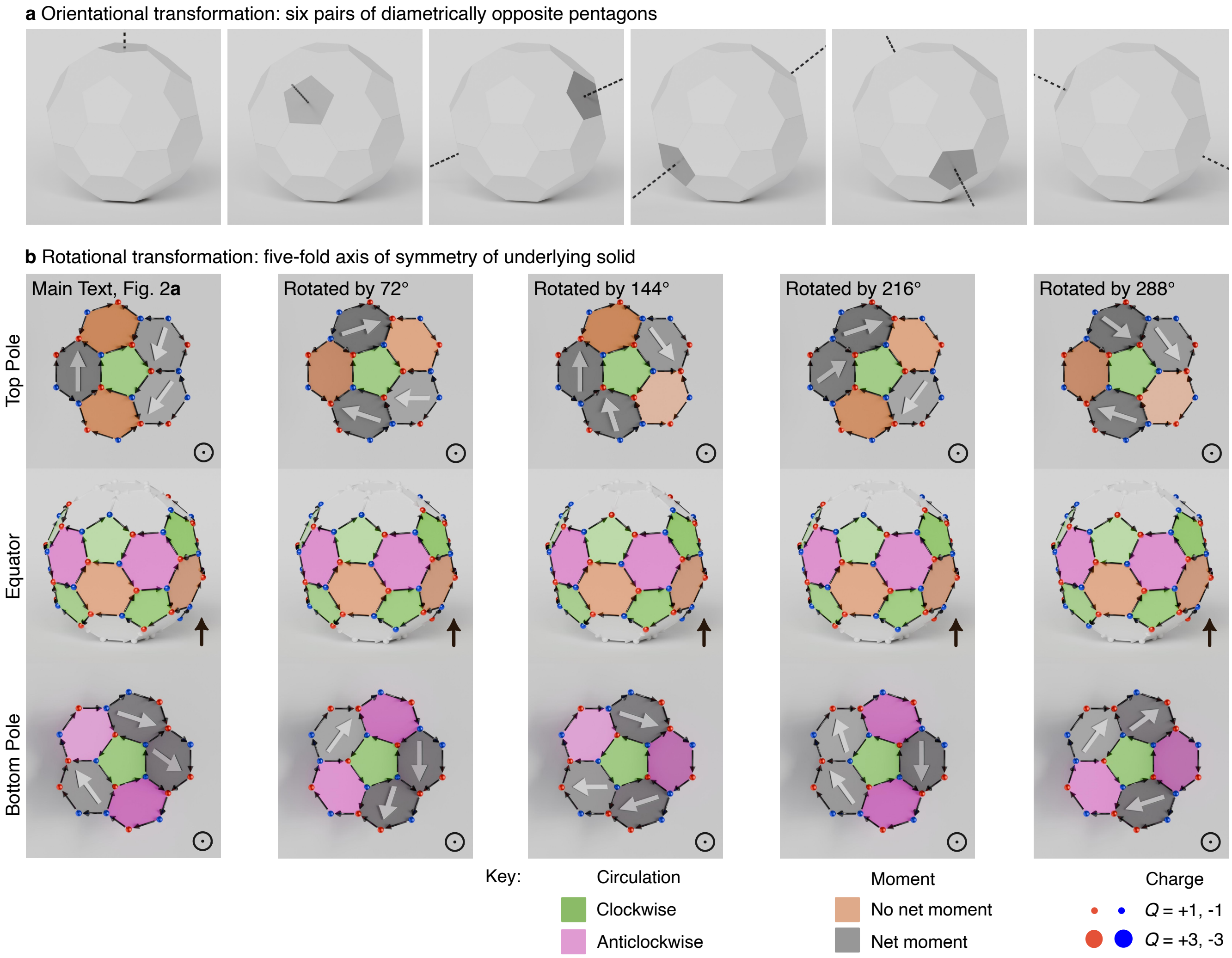

Figure S14. Global transformations connecting ground states of the buckyball artificial spin ice. **a**, The buckyball contains diametrically-opposite pentagons at six different locations. In the corresponding artificial spin ice lattice, pairs of topological defect poles can form at any of these six locations. A line connecting each pair of diametrically-opposite pentagons defines a symmetry axis. Each pair of diametrically-opposite pentagons is coloured in dark grey in a separate sub-panel. **b**, Around each symmetry axis, a new magnetic ground state can be generated from an existing one by a rotation through an integer multiple of 72°. The leftmost panel is the ground state configuration shown in main text, Fig. 2**a** and 4**c**. We use this configuration throughout this figure to illustrate how new ground states can be obtained by performing certain operations on this one. Faces are coloured according to the configuration of their associated spins, following the same colour scheme used in the main text. Figure continued on next page.

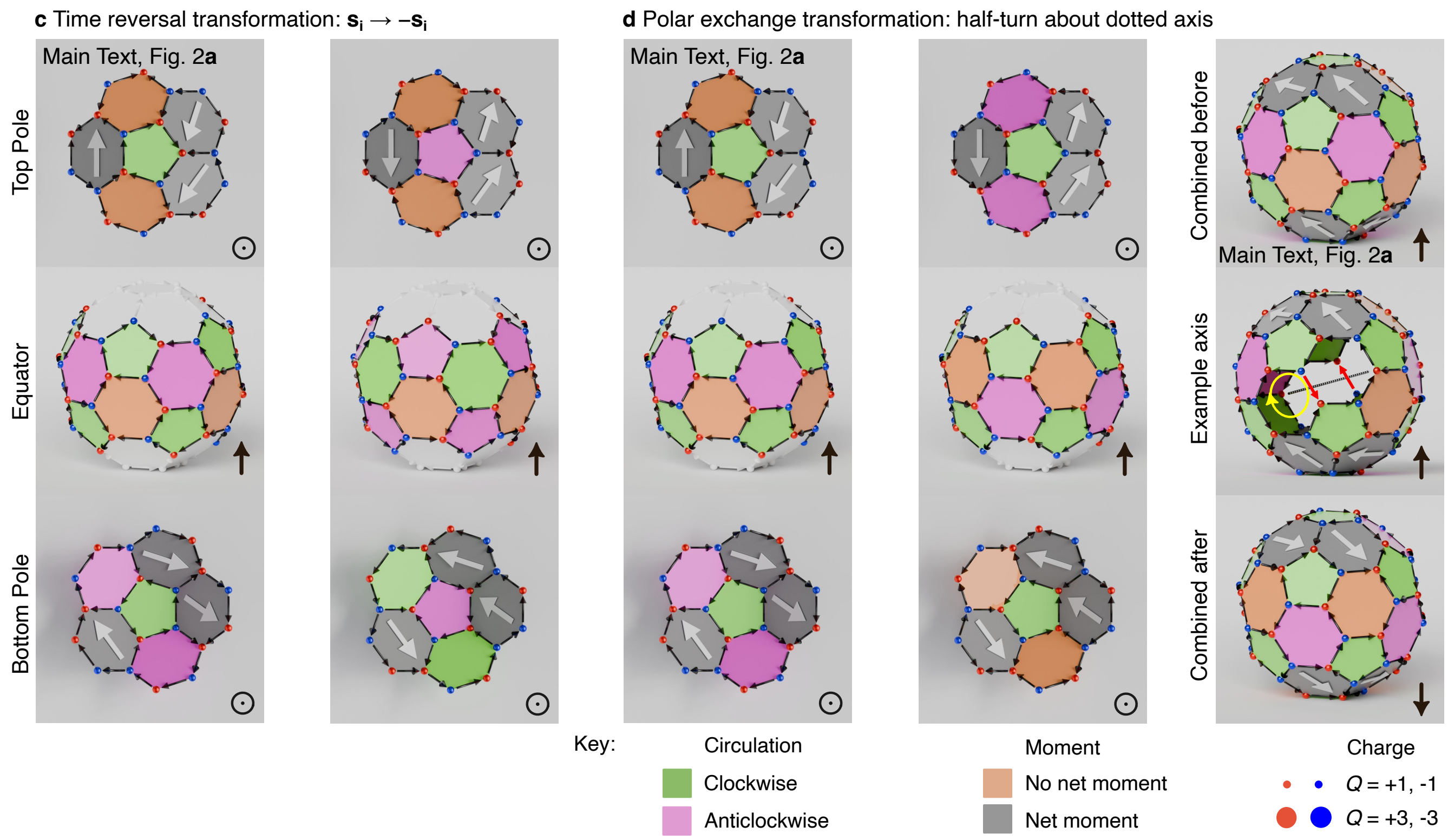

Fig. S14 (cont.). Global transformations connecting ground states of the buckyball artificial spin ice. c, Time reversal transformation, $\mathbf{s}_i \rightarrow -\mathbf{s}_i$, in which flipping all spins reverses the circulation of every flux-closed face and inverts the net moment of those faces that have one. **d**, Polar-exchange transformation, corresponding to a half-turn rotation about an axis passing through the centres of two spins in the equator located on opposite sides of the buckyball lattice. The third column illustrates this transformation: the top sub-panel shows the combined motifs of the north pole, equator, and south pole; the middle sub-panel presents the same configuration from a slightly different angle with the coloured faces removed and an example rotation axis, which passes through the two highlighted red spins; and the bottom sub-panel shows the resulting configuration after performing the half-turn about this axis. This transformation effectively swaps the spin configurations of the poles without changing the spin configuration of the equator. In the equator sub-panels, the black arrow points from the bottom pole to the top pole, along the line connecting the polar pentagons. In the pole sub-panels, we adopt a viewpoint looking down on the buckyball artificial spin ice; accordingly, the direction of the black arrow is indicated by a circled dot. In the 'Combined after' sub-panel in **d**, the arrow is inverted to indicate that what was previously the top pole is now at the bottom of the buckyball lattice.

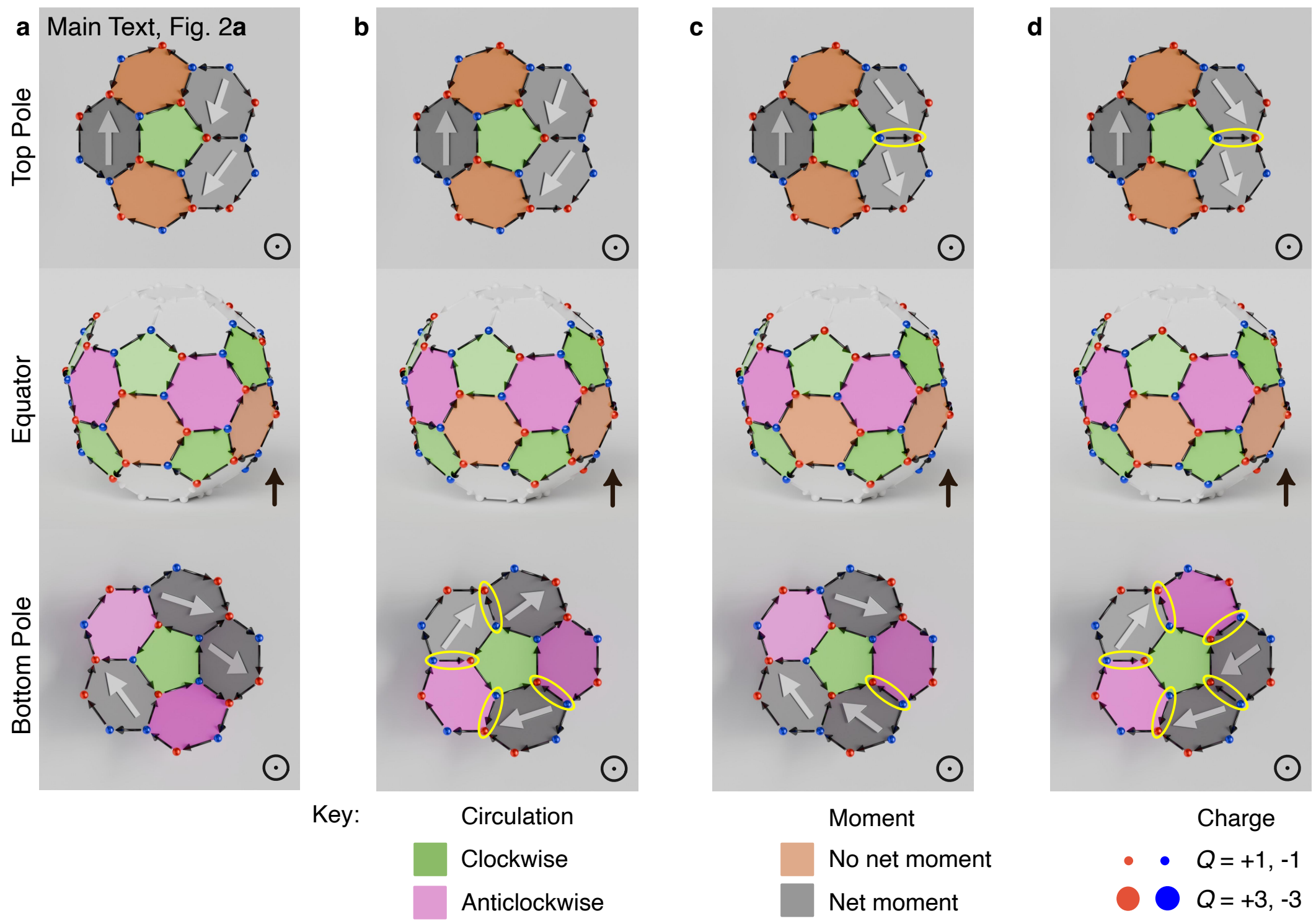

Figure S15. Degenerate ground state configurations obtained on performing local transformations. **a**, Top-pole, equator, and bottom-pole motifs corresponding to the configuration shown in the main text (Fig. 2**a**). In the subsequent panels, spins that are reversed relative to panel **a** are highlighted with yellow ellipses. **b**, Ground state obtained by reversing four spins in the bottom pole, with the top-pole and equator motifs unchanged. **c**, Ground state obtained by reversing two specific spins, one in each pole. **d**, Ground state obtained by reversing the same top-pole spin as in panel **c**, but this time together with the five spins in the bottom pole that adjoin the central flux-closed pentagonal face. All remaining ground states can be generated from these four configurations by applying the transformations shown in Fig. S14. In the equator sub-panels, the black arrow points from the bottom pole to the top pole, along the line connecting the polar pentagons. In the pole sub-panels, we adopt a viewpoint looking down on the buckyball artificial spin ice; accordingly, the direction of the black arrow is indicated by a circled dot.

Supplementary Note 8 Vertex-Based Buckyball Artificial Spin Ice

In the main text, we were careful to emphasise that our construction of the buckyball artificial spin ice—with nanomagnets arranged on the midpoints of the *edges* of the solid—was the form most likely to be realised using current fabrication methods. However, there exist other ways in which to construct an artificial spin lattice based on the buckyball. Indeed, existing computational work has treated the cases in which spins are placed on the *vertices* of a regular buckyball. These spins can either be Ising-like[13] or Heisenberg XYZ spins[14,15], coupled through a nearest-neighbour exchange. As we noted in the main text, a lattice in which spins are located on the vertices bears the most resemblance to 'molecular magnets', in which magnetic transition metals are seeded into a larger non-magnetic superstructure.

To determine whether such a model would provide as rich a spectrum of behaviour as the buckyball artificial spin ice discussed in the main text, we simulated a buckyball lattice in which Ising spins are located on the vertices and coupled through a dipolar interaction. For convenience, we will refer to this as the *vertex-based* buckyball artificial spin ice, to distinguish it from the *edge-based* construction, which is the central focus of our work. The spins in the vertex-based lattice are constrained to point either towards or away from the centre of the solid, and are coupled through the dipolar interaction [Eq. (S1)]. We perform a simulated anneal with single spin flips only, using the approach described in Supplementary Note 1. Loop moves cannot be used for such a system since they are no longer relevant for spins that do not point head-to-tail. The magnetic configuration after a simulated anneal is shown in Fig. S16 where spins that point into (out of) the centre of the solid are coloured red (blue). While the dipolar interaction between nearest-neighbours favours their antiparallel alignment, such an arrangement cannot be achieved everywhere on the surface of the vertex-based buckyball artificial spin ice. As a result, the system is highly frustrated and no system-spanning order emerges.

We now explore the thermal properties of the vertex-based buckyball artificial spin ice, which are shown in Fig. S17. In contrast to the edge-based buckyball artificial spin ice, the specific heat capacity exhibits only one peak at a temperature $k_B T/J_{1\text{nn}} \approx 0.9$ (black dashed line in Fig. S17**a**). It turns out that the vertex-based buckyball lattice is more frustrated than the edge-based buckyball artificial spin ice, which can be deduced from the higher residual entropy, $S_0 = 0.161(2)k_B$ per spin (c.f. $S_0 = 0.069(3)k_B$ per spin for the edge-based lattice). Nevertheless, just like the edge-based buckyball artificial spin ice, the vertex-based buckyball retains a net moment M down to zero temperature (Fig. S17**b**). Spin-spin correlations for the first three nearest neighbours are displayed in Fig. S17**c**; the behaviour of these is similar to that of the first three charge-charge correlations shown for the edge-based artificial spin ice in the main text, Fig. 3**c**, although the correspondence is not exact.

It is surprising that the temperature dependence of the heat capacity of the vertex-based lattice is so simple compared with that of the edge-based buckyball artificial spin ice. However, this can be explained by the fact that, in our edge-based buckyball artificial spin ice, there are several ways in which the system can lower its energy. Going from high temperature to low temperature, these are:

1. through the creation of ice-rule vertices;

2. by establishing charge order among those vertices;

3. by the flux closure of spins associated with individual faces; and

4. by tiling flux-closed motifs across the surface of the lattice.

These effects are all a result of cooperative phenomena of many spins, with important interactions between spins on opposite sides of the buckyball. Each of these effects occur at different temperatures since they

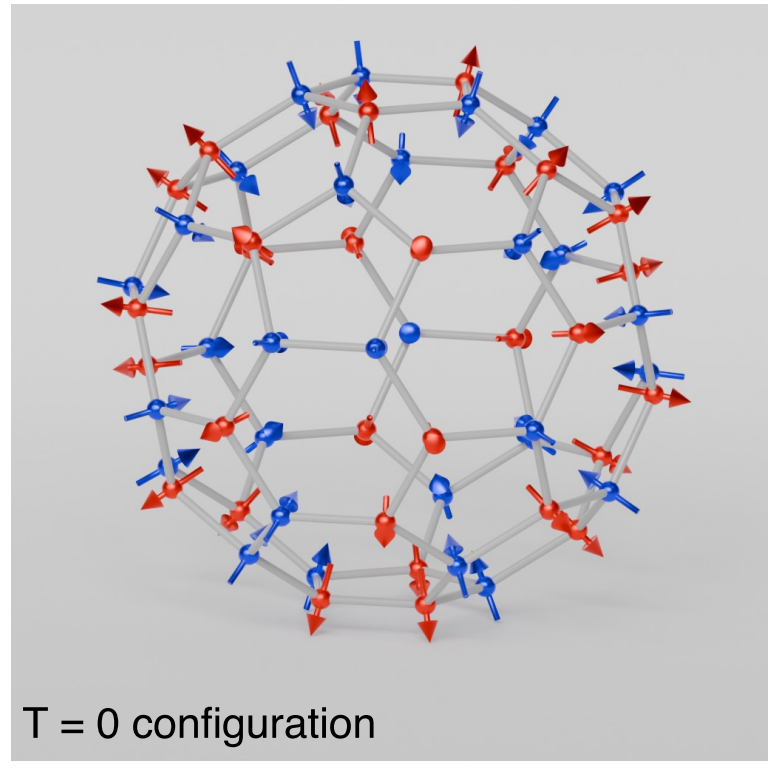

Figure S16. Typical ground state of the vertex-based buckyball artificial spin ice after a simulated anneal. Ising spins are arranged on the vertices of a regular buckyball ($N_{\text{spins}} = 60$), and are coupled through the dipolar interaction. Spins are constrained to point either towards (blue) or away from (red) the centre of the buckyball. The dipolar interaction promotes antiparallel alignment of nearest-neighbour spins. However, not all first-nearest neighbour interactions can be satisfied, leading to frustration.

are governed by different interaction scales, and a combination of them leads to the five crossovers as shown in Fig. 1**c** of the main text.

In the vertex-based buckyball, the analogue of the positive and negative charges in the edge-based buckyball are spins that point towards and away from the centre of the buckyball. Thus, as in the edge-based buckyball artificial spin ice, which hosts an imperfect charge crystal, the vertex-based buckyball exhibits an imperfect antiparallel alignment of nearest-neighbour spins. However, it does not support the formation of large, low-energy spin motifs. This contrasts with the the edge-based lattice where the placement of the spins and their mutual dipolar coupling explicitly lead to the formation of head-to-tail loops that, in combination with the curved, closed topology, lead to topological defects in the ground state.

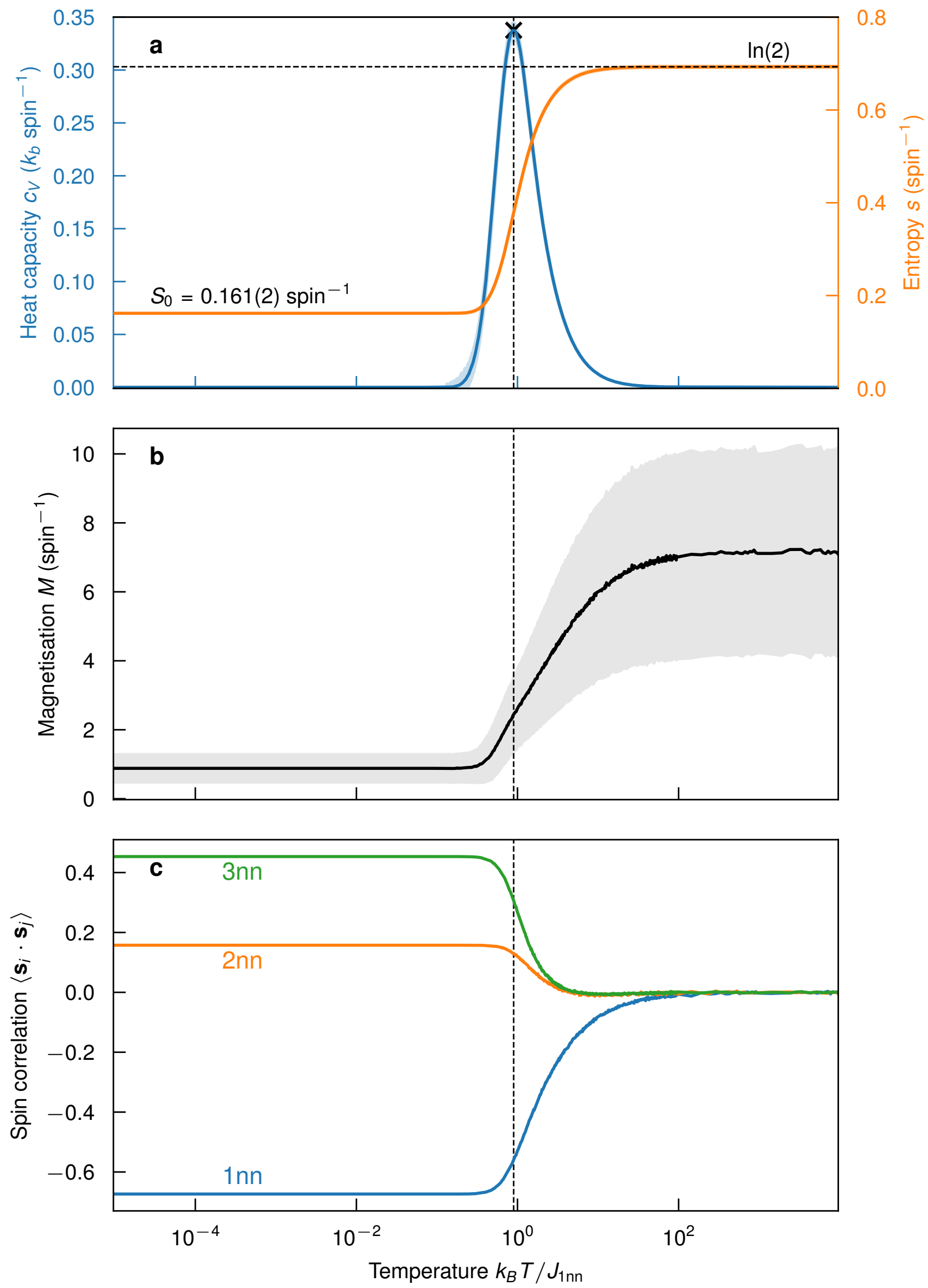

Figure S17. Thermodynamics of a vertex-based buckyball artificial spin ice. a, Specific heat capacity c_V (blue curve, left axis) and entropy s (orange curve, right axis) as a function of temperature when Ising spins are placed on the vertices of a regular buckyball (see Fig. S16). The vertex-based system orders in a single step, with a crossover at $k_BT/J_{1nn} \approx 0.90$, and has a residual entropy, $S_0 = 0.161(2)k_B$ per spin. The residual entropy S_0 is related to the number of possible ground states Ω through $S_0 = (1/N)\log(\Omega)$, where N is the number of spins. From this, it follows that that there are around 15000 possible ground states in the vertex-based lattice. **b**, Magnetisation per spin as a function of temperature. The system retains a small net moment down to zero temperature. **c**, Spin-spin correlations $\langle \mathbf{s}_i \cdot \mathbf{s}_j \rangle$ as a function of temperature for first-, second- and third-nearest neighbours (blue, orange and green curves, respectively). The behaviour of these correlations is reminiscent of the charge correlators for the buckyball artificial spin ice, c.f. main text, Fig. 3**b**. The key similarity is that the spins in the vertex-based buckyball (charges in the edge-based buckyball) try to be surrounded by spins pointing in the opposite direction (opposite charges), leading to an imperfect spin (charge) crystal. Because the spins in the vertex-based buckyball do not form head-to-tail loops, the thermal properties are not as rich, with only one crossover compared with the five for the edge-based buckyball artificial spin ice explored in the main text. The data are averaged over 5000 independent runs; shaded regions indicate $\pm 1\sigma$ around the mean value.

Supplementary References

1. Melko, R. G., den Hertog, B. C. & Gingras, M. J. P. Long-Range Order at Low Temperatures in Dipolar Spin Ice. *Phys. Rev. Lett.* **87**, 067203 (2001).
2. Melko, R. G. & Gingras, M. J. Monte Carlo studies of the dipolar spin ice model. *J. Physics: Condens. Matter* **16**, R1277 (2004).
3. Macêdo, R., Macauley, G. M., Nascimento, F. S. & Stamps, R. L. Apparent ferromagnetism in the pinwheel artificial spin ice. *Phys. Rev. B* **98**, 014437 (2018).
4. Andriushchenko, P. Influence of cutoff dipole interaction radius and dilution on phase transition in kagome artificial spin ice. *J. Magn. Magn. Mater.* **476**, 284–288 (2019).
5. Sendetskyi, O. *et al.* Continuous magnetic phase transition in artificial square ice. *Phys. Rev. B* **99**, 214430 (2019).
6. Hofhuis, K. *et al.* Thermally superactive artificial kagome spin ice structures obtained with the interfacial Dzyaloshinskii-Moriya interaction. *Phys. Rev. B* **102**, 180405 (2020).
7. Macauley, G. M. *et al.* Tuning magnetic order with geometry: Thermalization and defects in two-dimensional artificial spin ices. *Phys. Rev. B* **101**, 144403 (2020).
8. Cheenikundil, R. & Hertel, R. Switchable magnetic frustration in buckyball nanoarchitectures. *Appl. Phys. Lett.* **118** (2021).
9. Cheenikundil, R., Bauer, J., Goharyan, M., d'Aquino, M. & Hertel, R. High-frequency modes in a magnetic buckyball nanoarchitecture. *APL Mater.* **10** (2022).
10. Nisoli, C., Moessner, R. & Schiffer, P. Colloquium: Artificial spin ice: Designing and imaging magnetic frustration. *Rev. Mod. Phys.* **85**, 1473–1490 (2013).
11. Farhan, A. *et al.* Exploring hyper-cubic energy landscapes in thermally active finite artificial spin-ice systems. *Nat. Phys.* **9**, 375–382 (2013).
12. Schánilec, V. *et al.* Bypassing Dynamical Freezing in Artificial Kagome Ice. *Phys. Rev. Lett.* **125**, 057203 (2020).
13. Mhirech, A., Aouini, S., Alaoui-Ismaili, A. & Bahmad, L. Monte Carlo Study of the Magnetic Properties in a Fullerene-Like Structure: X20, X60, or X70. *J. Supercond. Nov. Magn.* **30**, 925–930 (2017).
14. Coffey, D. & Trugman, S. A. Magnetic properties of undoped C_{60}. *Phys. Rev. Lett.* **69**, 176–179 (1992).
15. Szabó, A., Capponi, S. & Alet, F. Noncoplanar and chiral spin states on the way towards Néel ordering in fullerene Heisenberg models. *Phys. Rev. B* **109**, 054410 (2024).
