# Supplementary material for: Five-stage ordering to a topological-defect-mediated ground state in a buckyball artificial spin ice
Source: Commun Phys. 2026 May 26;9(1):288. doi: 10.1038/s42005-026-02663-y (PMC13549942; doi:10.1038/s42005-026-02663-y)
Supplement: Supplementary file 3 [file 42005_2026_2663_MOESM3_ESM.pdf]

Description of Additional Supplementary Files

File name- Supplementary Movie 1

File description- Magnetic configuration of the buckyball artificial spin ice in the Hexagon II sector, corresponding to Fig. 2a.

File name- Supplementary Movie 2

File description- Magnetic configuration of the buckyball artificial spin ice in the Hexagon I sector, corresponding to Fig. 2b

File name- Supplementary Movie 3

File description- Magnetic configuration of the buckyball artificial spin ice in the Pentagon sector, corresponding to Fig. 2c.

File name- Supplementary Movie 4

File description- Magnetic configuration of the buckyball artificial spin ice in the Charge-Ice sector, corresponding to Fig. 2d.

File name- Supplementary Movie 5

File description- Magnetic configuration of the buckyball artificial spin ice in the Spin-Ice sector, corresponding to Fig. 2e.

File name- Supplementary Movie 6

File description- Magnetic configuration of the buckyball artificial spin ice in the Paramagnetic sector, corresponding to Fig. 2f.
